# Toward standardized iPSC testing: Insights from a multi-year international Quality Assessment Round

**DOI:** 10.1016/j.stemcr.2026.102857

**Published:** 2026-03-19

**Authors:** Alice Hägg, Rachel Wood, Ayako L. Mochizuki, Keren Abberton, Elsa Abranches, Belén Alvarez-Palomo, Ricardo Baptista, Raiana Andrade Quintanilha Barbosa, Jacqueline Barry, Adriana Bastos Carvalho, Annelise Bennaceur Griscelli, Antonio Carlos Campos de Carvalho, Diana Chaker, Hong Chang, Hye Young Choi, Margarita Codinach, Begoña Arán Corbella, Scott Cowan, Sarah Jane Dickerson, Ngaire Elwood, Xueling Fan, Maxime Feyeux, Maddy Forrester, Andrew Gaffney, Solenn M. Guilbert, Hye-Yeong Ha, Adam J. Hirst, Arwen L. Hunter, Leanne G. Jamieson, Robert N. Judson, Yonehiro Kanemura, Tais Hanae Kasai-Brunswick, Jung-Hyun Kim, Howard Kim, Manisha Kintali, Siddharth Krishnan, Bernd Kuebler, Chui Yu Lau, Wilson Li, Amanda Mack, Michael R. MacLeod, Marinna Madrid, Hiroaki Mamiya, Lucie Manache-Alberici, Dragoş Mărginean, Olivier Mentre, Stefanie L. Morgan, Joanne Mountford, Humayun Munir, Siemon H.S. Ng, Haruna Ogawa, Steve Oh, Hidetaka Ohara, Keiko Oono, Niall Park, Lygia V. Pereira, Izabella Pereira da Silva Bezerra, Alexandru Robert Podovei, Sergio Querol, Jainy Raje, Angel Raya, Satoko Sakamoto, Raquel Sarafian, Kathleen Schmit, Silvia Selvitella, Gurbind Singh, Matthew J.K. Smart, Jihwan Song, Glyn Stacey, Stephen Sullivan, Miho Sumida, Cecile Terrenoire, Pei Tian, Elias Uhlin, José M.A. Vaquero, Anna Veiga, Jar Wei Vicky Wang, Katherine Warre-Cornish, Jamie Wood, Atsuyo Yamamoto, Gaojun Zhang, Takafusa Hikichi, Marc Turner, Anna Falk

**Affiliations:** 1 Department of Experimental Medical Science (EMV), Lund Stem Cell Center, Science for Life Laboratory, Sölvegatan 17 A11, 221 84 Lund, Sweden; 2 Scottish National Blood Transfusion Service, The Jack Copland Centre, 52 Research Avenue North, Heriot-Watt Research Park, Edinburgh EH14 4BE, UK; 3 CiRA Foundation, 53 Shogoin kawahara-cho, Sakyo-ku, Kyoto 606-8397, Japan; 4 Stem Cell Medicine, Murdoch Children’s Research Institute, 50 Flemington Rd, Parkville, VIC 3052, Australia; 5 UK Stem Cell Bank, Medicines and Healthcare Products Regulatory Agency (MHRA), South Mimms Campus, Blanche Lane, South Mimms, Hertfordshire EN6 3QG, UK; 6 Banc de Sang i Teixits, Barcelona, Spain; 7 SmartCella, Alfred Nobels Allé 150, 146 48 Tullinge-Stockholm, Sweden; 8 Research Center for Precision Medicine, Carlos Chagas Filho Biophysics Institute Federal University of Rio de Janeiro, Rua Carlos Chagas Filho 373, Ilha do Fundão, Rio de Janeiro, RJ 21941-902, Brazil; 9 Cell and Gene Therapy Catapult, 12th Floor Tower Wing, Guy’s Hospital, Great Maze Pond, London SE1 9RT, UK; 10 University Paris-Saclay, INSERM, UMS 45 CITHERA – Center for iPS Cell Therapies, National Infrastructure INGESTEM, Paris, France; 11 Center for Commericialization of Regenerative Medicine (CCRM), #1002, 661 University Ave, Toronto, ON M5G 1M1, Canada; 12 National Center for Stem Cell and Regenerative Medicine, National Institute of Health, 202 Osongsaengmyeong2-ro, Osong-eup, Heungdeok-gu, Cheongju-si, Chungcheongbuk-do 28160, South Korea; 13 Barcelona Stem Cell Bank, Regenerative Medicine Program, Institut d'Investigació Biomèdica, IDIBELL, P-CMR[C]-Program of Translation of Regenerative Medicine in Catalonia, Catalonia, Spain; 14 FUJIFILM Cellular Dynamics Inc, Madison, WI 53711, USA; 15 Department of Paediatrics, University of Melbourne, Parkville, VIC 3052, Australia; 16 TreeFrog Therapeutics, 30 Av. Gustave Eiffel Bâtiment A, 33600 Pessac, France; 17 STEMCELL Technologies, 1618 Station St, Vancouver, BC V6A 1B6, Canada; 18 Department of Biomedical Research and Innovation, Institute for Clinical Research, NHO Osaka National Hospital, Osaka 540-0006, Japan; 19 New York Stem Cell Foundation Research Institute, 619 West 54th Street, New York, NY 10019, USA; 20 Dark Horse Consulting Group, 1255 Treat Blvd # 230, Walnut Creek, CA 94597, USA; 21 Cellino, 750 Main St, Cambridge, MA 02139, USA; 22 College of Pharmaceutical Sciences, Ritsumeikan University, 1-1-1 Noji-Higashi, Kusatsu, Shiga 525-8577, Japan; 23 Notch Therapeutics, Toronto, ON, Canada; 24 Stem Cell Group, Bioprocessing Technology Institute, A∗STAR, Singapore, Singapore; 25 Sumitomo Pharma Co., Ltd., 33-94, Enoki-cho, Suita, Osaka 564-0053, Japan; 26 National Laboratory for Embryonic Stem Cells (LaNCE), Department of Genetics and Evolutionary Biology, Institute of Biosciences, University of São Paulo, São Paulo, SP 05508-090, Brazil; 27 Josep Carreras Leukaemia Foundation, Muntaner 383, 2nd, 08021 Barcelona, Spain; 28 BlueRock Therapeutics, Toronto, ON, Canada; 29 ICREA, Regenerative Medicine Program, Institut d'Investigació Biomèdica de Bellvitge (IDIBELL), P-CMR[C]-Program of Translation of Regenerative Medicine in Catalonia, Center for Networked Biomedical Research on Bioengineering, Biomaterials and Nanomedicine (CIBER-BBN), Physiological Sciences Department, University of Barcelona, Barcelona, Spain; 30 Centre for Stem Cell Research (a unit of BRIC-inStem, Bengaluru), Christian Medical College Campus, Bagayam, Vellore, Tamil Nadu 632002, India; 31 International Stem Cell Biobanking Initiative, Barley, Hertfordshire SG88HZ, UK; 32 National Stem Cell Resource Centre, Institute of Zoology, Chinese Academy of Sciences, Beijing 100190, China; 33 Beijing Institute for Stem Cell Regenerative Medicine and Chinese Academy of Sciences, Beijing 100101, China; 34 Lindville Bio, 69 Warrender Park Road, Edinburgh, UK; 35 Medicines and Healthcare products Regulatory Agency, London, UK; 36 Century Therapeutics, 3624 Market Street, #5 Floor West, Philadelphia, PA 19104, USA; 37 CHA University, Rm 604, CHA Bio Complex, 335 Pangyo-ro, Bundang-gu, Seongnam-si, Gyeonggi-do 13488, Republic of Korea; 38 Formerly Medicines and Healthcare Products Regulatory Agency, London, UK; 39 BlueRock Therapeutics (2019) and Notch Therapeutics (2023/2024), Toronto, ON, Canada

**Keywords:** induced pluripotent stem cells, regenerative medicine, cell therapy manufacturing, quality control, genomic integrity, flow cytometry, qPCR, inter-laboratory reproducibility, standardization, global harmonization

## Abstract

Despite rapid clinical translation, induced pluripotent stem cell (iPSC)-derived therapies face limited global adoption. Harmonized quality control (QC) remains absent, with even fundamental parameters evaluated inconsistently across laboratories. To address this, we conducted two international Quality Assessment Rounds (QARs): QAR 2019 (18 sites, 11 countries) and QAR 2023 (23 sites, 12 countries), evaluating flow cytometry-based assessment of the undifferentiated state and qPCR-based genomic integrity testing. QAR 2019 showed high consistency in genomic integrity testing, while uncovering substantial variability in flow cytometry, prompting QAR 2023 to introduce standardized workflows. These improvements enabled systematic, cross-site evaluation of marker performance across cell states, identifying OCT3/4, TRA-1-60, and SSEA5 as consistently robust pluripotency-associated markers. This global benchmarking effort provides the first empirical multi-site evidence for reproducible iPSC QC and marker-level reliability. Together, these findings establish a foundation for harmonized QC supporting interoperable iPSC banks, regulatory alignment, and scalable manufacturing of globally accessible regenerative therapies.

## Highlights

• Harmonized flow cytometry workflows with thresholds reduce analysis subjectivity
• OCT3/4, TRA-1-60, and SSEA5 are robust and effective markers for iPSC QC
• SSEA4 shows persistent expression in early differentiating iPSCs
• qPCR serves as a standardized complementary tool for routine genomic screening

## Graphical abstract

**Figure:**
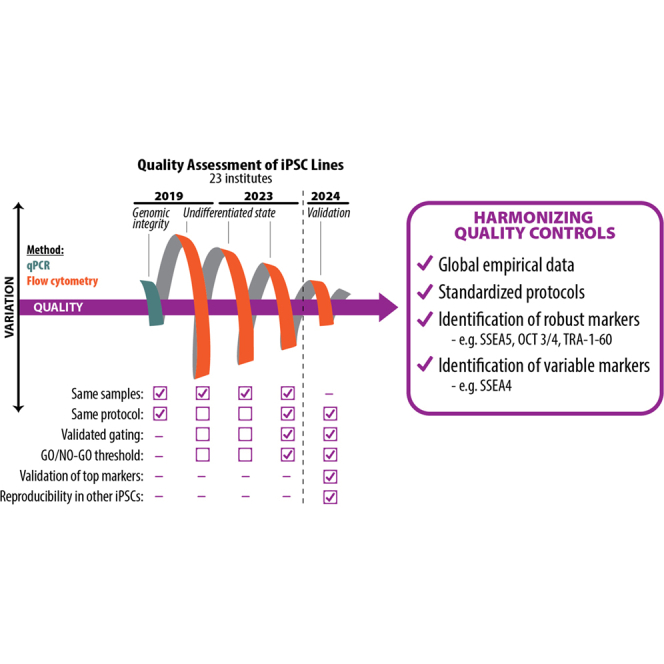

## Teaser

Falk, Turner, and colleagues perform a global, multi-institute study integrating standardized assays with empirical benchmarking to generate data-driven evidence for reproducible iPSC QC protocols and reliable marker panels. They identify robust flow cytometry markers for monitoring undifferentiated status, emphasize harmonized workflows, and show that qPCR provides a complementary genomic integrity screen, supporting consistent quality control of iPSC lines.

## Introduction

Induced pluripotent stem cells (iPSCs) have attracted significant interest as a renewable source of cells for the scalable manufacture of advanced therapy medicinal products (ATMPs), suitable for both autologous and allogeneic applications. Pluripotent cell-based ATMPs are advancing rapidly through clinical pipelines worldwide, with an increasing number of trials and several products approaching regulatory approval (hPSCreg; Kirkeby et al., 2025). These advances highlight the substantial therapeutic potential of iPSC-based therapies, while underscoring the need for consistent, scalable manufacturing processes and high-quality starting materials.

The widespread adoption of iPSC-based therapies will depend on establishing international iPSC banks to ensure a reliable and accessible supply. Achieving this goal requires robust quality control (QC) strategies that enable standardized testing and well-defined release criteria across diverse clinical, regulatory, and geographic contexts. Despite increasing clinical and regulatory activity, no comprehensive, globally harmonized framework has yet been universally accepted for assessing iPSC identity, purity, and genomic integrity. Existing regulatory guidelines are primarily adapted from earlier biotechnological standards and remain fragmented and inadequately implemented across regions (Barry et al., 2015; Martins and Ribeiro, 2025), reflecting the early stage of development for PSC-based products. While some PSC-derived therapies have entered phase 1–3 clinical trials, none have yet achieved full market authorization (hPSCreg). Regulatory approvals from agencies such as the Food and Drug Administration and European Medicines Agency represent critical milestones, and the associated QC strategies are often regarded as *de facto* guidelines for the cell therapy field. However, these regulatory frameworks are insufficient to ensure global scalability, as they typically involve product- and jurisdiction-specific requirements that must be extensively re-validated when applied to different products, processes, or facilities. In the absence of harmonized standards, each therapy or manufacturing site is treated as a standalone case, requiring *de novo* validation that slows progress, increases costs, and limits interoperability. This lack of coordination presents a major barrier to the widespread clinical implementation of iPSC-based therapies.

Importantly, the lack of standardization in QC practices could also hinder global equity. Many low- and middle-income countries may lack the infrastructure or regulatory capacity to implement and validate complex QC workflows without additional support (World Health Organization, 2018). Without harmonized, accessible workflows, these regions risk exclusion from both the development and the benefits of iPSC-based therapies. A globally accepted QC framework would allow centralized iPSC banks to distribute high-quality material worldwide (Escribá et al., 2024), while allowing local laboratories to verify identity and safety using shared, validated protocols. In this context, assay variability is not merely a technical issue, but a structural barrier to equitable progress in regenerative medicine.

Reliable assessment of pluripotency is essential for confirming iPSC nature. Although the *in vivo* teratoma assay has traditionally been considered a gold standard, its reliance on animal models, cost, and low throughput limit its routine use. Alternative methods, such as tri-lineage differentiation and transcriptomic profiling, have been explored, yet no consensus on a single best practice has emerged (International Stem Cell Initiative, 2018). Many of these approaches rely on the ability of cells to differentiate, highlighting the relevance of functionally oriented assays for pluripotency assessment (Abranches et al., 2020).

Flow cytometry remains the most widely used technique for assessing the undifferentiated state of iPSCs. Typically, co-expression of at least one intracellular marker (e.g., OCT3/4, NANOG, SOX2) and one surface antigen (e.g., TRA-1-60 or SSEA4) is evaluated, with >70% positive expression generally expected for undifferentiated iPSCs (Andrews et al., 2015; Sullivan et al., 2018). Each pluripotency-associated marker, however, has unique context-specific strengths and limitations. Misinterpretation, such as relying on a single marker or neglecting assay context, can lead to inaccurate conclusions about the pluripotent state of the iPSC bank. Careful validation of marker combinations and assay design is, therefore, critical to ensure accurate interpretation and reliable assessments. Despite this, substantial variability persists across laboratories in the selection of marker panels, antibody clones, staining protocols, and data interpretation thresholds. Beyond protocol differences, the performance of individual markers can vary by site and sample state, underscoring the need for systematic, multi-site benchmarking to identify the most reliable markers for both undifferentiated and differentiated samples.

Genomic integrity represents another cornerstone of iPSC quality, as recurrent chromosomal abnormalities can arise during reprogramming and extended culture (Andrews et al., 2017; Halliwell et al., 2020; International Stem Cell Initiative et al., 2011; Merkle et al., 2017; Weissbein et al., 2019). Some alterations may confer a selective growth advantage (Avery et al., 2013) or mirror genomic alterations seen in cancers (Peterson and Loring, 2014; Wuputra et al., 2020), raising safety and efficacy concerns. A range of complementary assays, including Giemsa-banding karyotype (G-banding), chromosomal microarray (CMA), next-generation sequencing (NGS), fluorescent *in situ* hybridization, and polymerase chain reaction (PCR), is often used to assess genomic stability, as each offers distinct detection capabilities. G-banding remains a widely used first-line screen, enabling identification of large-scale abnormalities (>5–10 Mb), translocations (balanced and unbalanced), and high-level mosaicism (Abranches et al., 2020). CMA and NGS provide higher resolution, enabling detection of submicroscopic copy number variants and point mutations not detected by G-banding, but may miss balanced rearrangements and low-level mosaicism. The International Stem Cell Initiative (www.iscbi.org) has recommended regular genomic assessment, including NGS at least retrospectively, to ensure the safety of iPSC-derived products (Abranches et al., 2020; Andrews et al., 2015; International Stem Cell Initiative, 2018; Kim et al., 2019).

Although expert guidelines and regulatory filings provide insight into acceptable QC strategies (International Society for Stem Cell Research, 2025; Lovell-Badge et al., 2021; Mahalatchimy et al., 2025; Martins and Ribeiro, 2025; Sullivan et al., 2018), the field lacks systematic, empirical evidence on inter-laboratory assay performance. No large-scale benchmarking studies have evaluated reproducibility in flow cytometry or genomic QC for iPSCs, and publicly available comparative datasets remain limited in scope. Without such data, reproducibility remains unverified, interoperability is constrained, and the clinical translation of iPSC-based therapies remains slower and more resource intensive than necessary.

To address this gap, the Global Alliance for iPSC Therapies (GAiT) launched the first coordinated international effort to benchmark two widely used iPSC QC assays: quantitative PCR (qPCR) for genomic integrity and flow cytometry for monitoring the undifferentiated state. Two Quality Assessment Rounds (QARs) were conducted: QAR 2019, organized in collaboration with the CiRA Foundation (CiRA_F), National Institute for Biological Standards and Control (NIBSC), and STEMCELL Technologies, and QAR 2023, conducted jointly with CiRA_F, involving 18 and 23 participating institutes, respectively, together spanning 12 countries. A QAR is a collaborative process through which scientists from different organizations work to ensure that testing practices yield consistent and comparable results. These GAiT QARs build upon the foundation established in the publication “Quality control guidelines for clinical-grade human induced pluripotent stem cell lines” (Sullivan et al., 2018) and are a part of a broader, ongoing international initiative aimed at facilitating the use of iPSC lines as standardized starting materials for cell therapies.

QAR 2019 revealed substantial inter-laboratory variability in flow cytometry results, while the genomic integrity assay showed consistent performance across sites. This variability, despite the use of nominally similar protocols, highlights the inherent challenges of achieving reproducibility through locally adapted practices. Building on these insights, QAR 2023 introduced standardized flow cytometry conditions and kit-based formats to enhance reproducibility and enable systematic evaluation of marker performance across sites and sample states. This approach enabled the identification of pluripotency‑associated markers that are consistently robust, as well as those that exhibit variability in undifferentiated and differentiated contexts.

This study represents the first global, multi-site initiative to generate empirical, data-driven evidence for reproducible iPSC QC assays. Specifically, it evaluates the consistency of flow cytometry-based identity assays and qPCR-based genomic integrity assessments. Unlike previous standardization efforts based primarily on expert consensus, our work provides empirical benchmarks across institutions, revealing marker-level reliability and informing the design of harmonized QC panels that reduce subjectivity and support interoperable workflows. These findings establish a practical foundation for international iPSC banks and bridge the gap between expert guidelines, regulatory-approved QC practices, and the scalable implementation required for global adoption. Ultimately, this work advances the development of harmonized QC frameworks essential for the safe, efficient, and equitable delivery of iPSC-based ATMPs worldwide.

## Results

### Current testing methods for iPSC lines

#### QAR 2019

In QAR 2019, participating institutes (hereafter referred to as participants) were surveyed prior to and during the QAR to better understand their testing practices and to inform interpretation of results. All 18/18 (100%) completed the survey. Only 5/18 (28%) reported adopting formal national or international quality standards for iPSC testing, covering areas such as sterility, human pathogen screening, karyology, and transgene integration analyses.

Genetic integrity testing was performed by 14/18 (78%) participants using methods including karyotyping, copy number variation (CNV)/single-nucleotide variant array analysis, DNA methylation profiling, and whole-genome sequencing (WGS). Among these, 6/14 (43%) reported using a standard operating procedure (SOP). Testing was carried out in-house by 8/14 (57%) and externally by 6/14 (43%).

Phenotypic characterization was performed by 14/18 (78%) participants, all of whom reported using both immunocytochemistry and flow cytometry. Of these, 7/14 (50%) confirmed using an SOP for at least one of the methods used. Testing was carried out in-house by 11/14 (79%), and 3/14 (21%) outsourced this work to an external laboratory.

Collectively, these findings indicate that while quality assessment practices are employed by the majority of organizations, there remains a clear need to standardize and validate testing protocols within a robust quality management system to ensure consistency and reliability of iPSC characterization across sites.

#### QAR 2023

A total of 18/23 participants (78%) completed the survey. Similar to QAR 2019, 4/18 (22%) reported adopting formal national or international quality standards for iPSC testing.

Genetic integrity testing was performed by 13/18 (72%) participants using methods such as single-nucleotide polymorphism (SNP) array, CNV by droplet digital PCR, karyotyping, and WGS. Among these, 9/13 (69%) reported having an SOP in place. Testing was carried out externally by 7/13 (54%), in-house by 4/13 (31%), and by both in-house and external laboratories by 3/13 (23%).

Phenotypic characterization was reported by 12/18 (67%) participants. Of these, 11/12 (92%) used an SOP and performed the testing in-house. Reported approaches included flow cytometry, germ layer assessment by qPCR or reverse-transcription PCR, and immunocytochemistry.

Genotyping was performed by 12/18 (67%) participants using methods such as karyotyping, microarray, WGS, SNP analysis, and NGS. Of these, 8/12 (67%) reported using an SOP, 2/12 (17%) did not, and 2/12 (17%) did not provide this information. Testing was carried out externally by 6/12 (50%), in-house by 5/12 (42%), and by both in-house and external laboratories by 1/12 (8%).

Taken together, these responses show that iPSC testing practices still vary widely between organizations. This lack of consistency highlights the need for standardized protocols and clear regulatory frameworks to ensure the safety, reproducibility, and scalability of future iPSC banks.

### Genetic integrity testing with a commercially available kit

QAR 2019 involved participants testing two blinded genomic DNA samples using a commercially available qPCR kit, designed for detection of 8 recurrent genomic abnormalities in human pluripotent stem cell (hPSC) cultures (www.stemcell.com/hpsc-genetic-analysis-kit). The two samples provided to participants were extracted from iPSC cultures that were known to exhibit either a normal diploid karyotype (CiRA G1) or contain an unbalanced structural rearrangement resulting in gain of the long arm of chromosome 1 (1q) (CiRA G2).

A total of 45 individual runs were performed: 11/18 (61%) participants chose to complete three runs each, 5/18 (28%) completed two runs, and 2/18 (11%) completed a single run. Each run consisted of the two genomic DNA samples (CiRA G1 and CiRA G2) and the genomic DNA control supplied with the kit. Participants were asked to interpret their own data and submit both raw data and analyzed results for independent evaluation. All genetic data were anonymized, and handling complied with General Data Protection Regulation and the UK Data Protection Act to protect personal genetic information.

**Figure 1:**
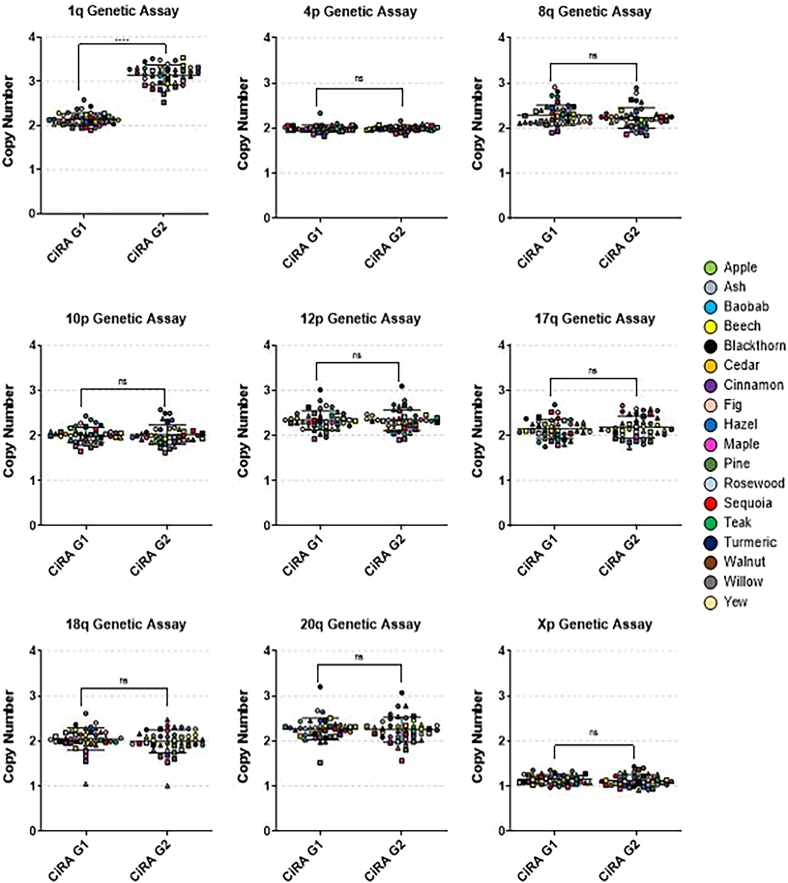
Genetic testing of blinded samples demonstrates broadly consistent results across participants in QAR 2019 Scatterplots show the distribution of copy number data across all valid runs. Each data point represents the median copy number from three technical replicates at a given genomic locus in an individual run. Circles, squares, and triangles denote first, second, and third runs, respectively. Error bars indicate the mean ± standard deviation across all runs for each locus (*n* = 43).

Figure 1 shows the median copy number across each region, calculated from three technical replicates per sample, using data collected from all participants. Of the 45 runs completed, 42 correctly identified the amplification of chromosome 1q in the CiRA G2 sample, while the CiRA G1 sample was accurately classified as diploid. Statistical analysis showed no significant differences in detected copy number between the CiRA G1 and CiRA G2 samples across all tested loci, except for chromosome 1q, which was consistently amplified in the abnormal sample (paired two-tailed *t* test, *p* ≤ 0.0001).

In the three remaining runs, two were flagged as indeterminate due to high variability between technical replicates. The third was classified as possibly abnormal, as the data were insufficient for the data analysis software to assign a confident result. This was attributable to a lower-than-expected copy number at chromosome 1q (2.52 copies) and the detection of two additional false-positive calls (deletion of chromosomes 10p and 18q). In all three cases, repeat testing would have been recommended.

### iPSC characterization by flow cytometry

#### QAR 2019

In QAR 2019, participants tested five fixed cell samples provided by two independent organizations (CiRA_F and NIBSC), following their routine protocols within the specifications outlined in the QAR 2019 subsection of the methods section. The CiRA_F samples were analyzed by all (18) participants. CiRA F1 represented an undifferentiated sample, and CiRA F2 was a spontaneously differentiated cell sample. All participants also analyzed the NIBSC samples; however, sample preparation issues limited inclusion of data from only four participants whose results met all QC criteria (>60% of total events within the P1 gate [i.e., the first flow cytometry gating region] and a P1 cell count >4,000). Data from participants whose samples failed QC criteria were excluded from further analysis.

To ensure appropriate data grouping, and given that the NIBSC samples were technical replicates, a mixed-effects model was employed to estimate differences between the CiRA F1 and NIBSC samples while accounting variability across markers and participants. The model predicted an average 0.1% difference among the NIBSC triplicates and a 1.5% absolute decrease in %PSC values for the NIBSC samples compared to CiRA F1. Consequently, both datasets were combined for analysis (Figure S1).

Testing of the undifferentiated samples showed reasonable consistency across participants, with most correctly identifying the undifferentiated cell line. In contrast, assessment of the CiRA F2 spontaneously differentiated sample exhibited substantial variability. Among markers, NANOG demonstrated the greatest inter-laboratory variability, whereas OCT3/4 and TRA-1-60 showed the least. The most commonly used markers for iPSC analysis were OCT3/4, TRA-1-60, and SSEA4, followed by TRA-1-81, SOX2, NANOG, and SSEA3.

Given the substantial variability observed in flow cytometry results in the QAR 2019 dataset, we aimed to re-evaluate this aspect under more controlled conditions. Accordingly, QAR 2023 was designed to systematically assess whether standardized flow cytometry protocols and kit-based reagents could improve inter-laboratory reproducibility.

#### QAR 2023 Quality Test 1 variables

In QAR 2023, three distinct fixed cell sample types (undifferentiated cells, spontaneously differentiated iPSCs, and a 1:1 ratio mixture of both) were analyzed by 23 participants using two separate quality tests. To understand which markers are routinely used in iPSC characterization, participants were first asked to analyze the samples by flow cytometry following their own protocols and reagents (Quality Test 1). Of 23 participants, 22 completed Quality Test 1, with 19/22 (86%) of participants choosing to perform two test runs and 3/22 (14%) choosing to perform one. The number of replicates per run varied, with 8/22 (36%) of runs testing samples once, 6/22 (27%) twice, and 8/22 (36%) three times. Table S1 provides additional details on the number of replicate measurements performed by each participant for each sample.

The number and choice of flow cytometry markers also varied among participants. Ten of 22 (45%) participants analyzed their samples using four different markers, while 8/22 (36%) used three markers (Table S2). The most popular marker within the QAR was OCT 3/4, with 22/22 (100%) of participants selecting this marker for analysis, followed by TRA-1-60 (19/22; 86%), SSEA4 (18/22; 82%), and SOX2 (9/22; 40.9%). The remaining markers were only used by five participants or fewer (Table S2).

The types of flow cytometer used by participants in QAR 2023 varied considerably. A total of 15 different flow cytometer models were used (Table S3), with the BD Accuri C6, BD FACS Canto II, and Miltenyi Biotec MACSQuant Analyzer 10 being the most commonly used.

#### QAR 2023 Quality Test 1 flow cytometry results

**Figure 2:**
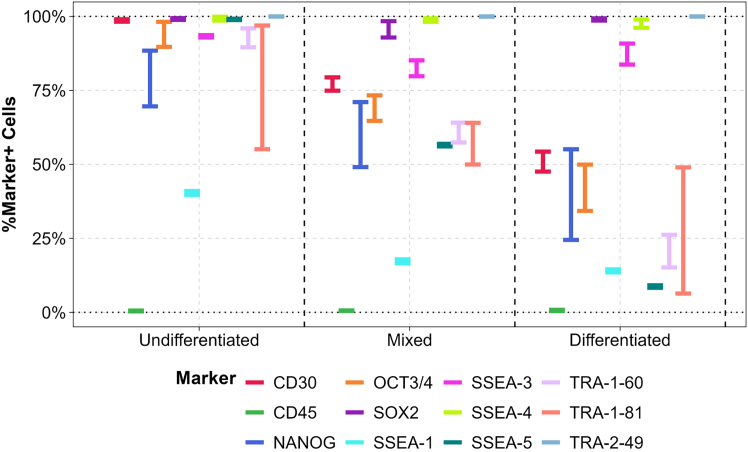
Percentage of marker-positive cells across sample types in QAR 2023 Quality Test 1 Overview of the percentage of marker-positive cells for each sample type (undifferentiated cells, 1:1 ratio mixture, and spontaneously differentiated iPSCs). Each marker is represented by a distinct color. Error bars indicate the 95% confidence interval of the mean, calculated using bootstrapping.

Figure S2 shows each participant’s performance in identifying the individual samples, offering insight into the effectiveness of each participant’s in-house flow cytometry panel and highlighting the variability observed across contributors. Based on these data, the percentage of cells positive for each marker tested was calculated as the mean across all participants’ data (Figure 2). Since the mixed sample combined undifferentiated and differentiated cells at a 1:1 ratio, marker expression was expected to scale proportionally. CD45, SOX2, SSEA4, and TRA-2-49 exhibited low variability between participants; however, their expression levels remained consistent across all sample types, limiting their utility in distinguishing differentiation status.

Conversely, NANOG and TRA-1-81 showed proportional changes in expression correlating with differentiation, decreasing as differentiation increased, but these markers displayed high inter-participant variability. Notably, four markers, CD30, OCT3/4, SSEA5, and TRA-1-60, combined low inter-participant variability with clear proportional changes in expression across sample types, indicating their suitability for assessing differentiation status.

To further investigate which markers most effectively distinguish differentiated from undifferentiated stem cells, the percentage of marker-positive cells in the differentiated samples was subtracted from that in the undifferentiated samples, yielding the difference in marker expression (Table 1). Markers exhibiting a difference in mean percentage of marker-positive cells greater than 30% were considered suitable for confirming stem cell differentiation. In Quality Test 1, the markers exceeding this threshold included SSEA5, TRA-1-60, TRA-1-81, OCT3/4, CD30, and NANOG.

**Table 1:**
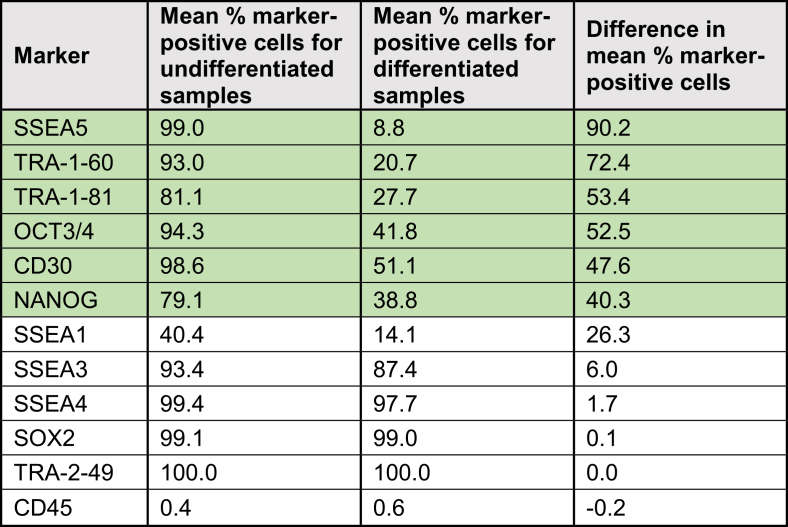
Mean percentage of marker-positive cells in undifferentiated vs. differentiated samples: Greater discrimination is achieved with a higher value for the difference in the percentage of marker-positive cells

Markers showing a >30% difference in the mean proportion of marker-positive cells are highlighted in green.

To evaluate marker consistency, markers showing no change across samples or analyzed by only one participant were excluded. The analysis then focused on four markers: NANOG, OCT3/4, TRA-1-60, and TRA-1-81, which were assessed for within-sample consistency (Table S4). For NANOG, OCT3/4, and TRA-1-60, differentiated cell samples exhibited the highest variability, with standard deviations of 41.4%, 36.4%, and 23.7%, respectively, whereas TRA-1-81 showed greatest variability in undifferentiated samples (33.7%). This variability reflects that some participants failed to detect expected expression changes for certain markers (Figure S2), contributing to the overall data spread.

#### QAR 2023 Quality Test 2 flow cytometry results

To evaluate whether standardizing staining protocols and reagents could minimize variability among organizations, all 23 participants conducted Quality Test 2. This test utilized standardized flow cytometry samples, reagents (including staining buffers and antibodies conjugated exclusively with Alexa Fluor 488 or fluorescein isothiocyanate [FITC] fluorochromes), and uniform staining and analysis procedures provided by CiRA_F. Figure S3 displays the comprehensive data from all participants for the undifferentiated, mixed, and differentiated samples. The results indicate that all participants, except for one identified as “Swallow,” accurately categorized each sample. An anomaly was observed for the participant “Canary,” who recorded an unusually high expression of PAX6 in the undifferentiated sample, as illustrated in Figure S3.

**Figure 3:**
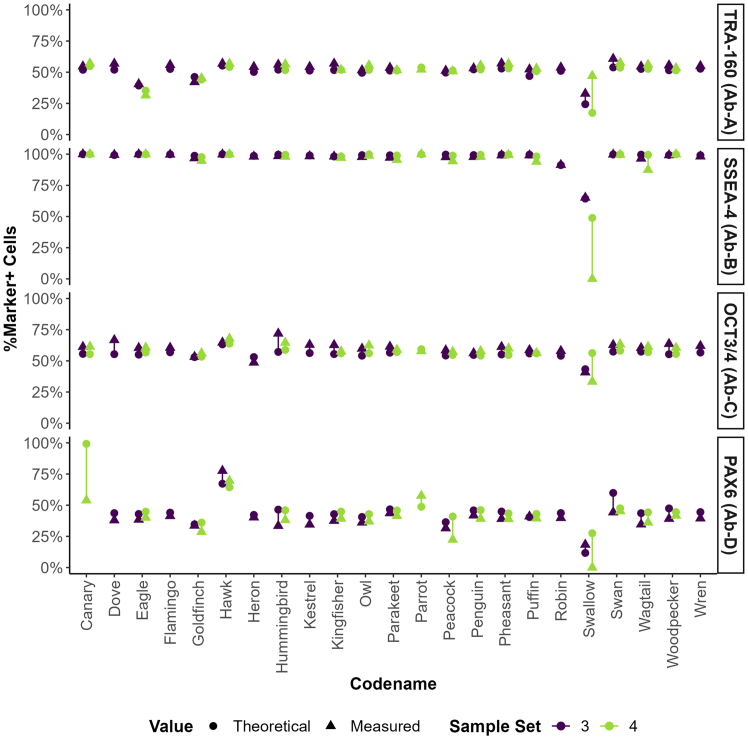
Expected versus observed marker expression in mixed samples in QAR 2023 Quality Test 2 Discrepancies between expected and observed levels of TRA-1-60, SSEA4, OCT3/4, and PAX6 are shown for each participant. The expected recovery represents the precise midpoint between undifferentiated and differentiated samples, reflecting the theoretical marker expression in a 1:1 mixture. Observed measurements are compared to this theoretical value to evaluate marker proportionality and participant performance.

To evaluate the effectiveness of the four markers in distinguishing among sample types, recovery of the mixed (1:1) samples was calculated for each participant. The expected recovery was defined as 100% of the theoretical value, derived from the average marker expression in the corresponding undifferentiated and differentiated samples. Figure 3 illustrates these results, while detailed values are provided in Table S5. Overall, 89.7% of results demonstrated recoveries within the range of 80%–125%, indicating that the selected markers performed consistently and proportionally across most participants.

However, as previously observed, the results from “Swallow” were inconsistent across all markers, deviating markedly from the overall trend. This pattern suggests potential issues related to sample handling, staining or analysis protocol execution or the quality of materials used in their analysis. Further investigation revealed that shipment of cell lines and reagents to “Swallow” took 16 days, compared to 0–8 days for the remaining 22 participants (Table S6). This extended transit time may have compromised reagent integrity and, more critically, the quality of the cell samples. Factors such as temperature fluctuations, potential fixative evaporation, or extended storage under suboptimal conditions may have altered cell morphology or marker detectability, possibly contributing to the inconsistencies observed in the data from “Swallow.”

**Figure 4:**
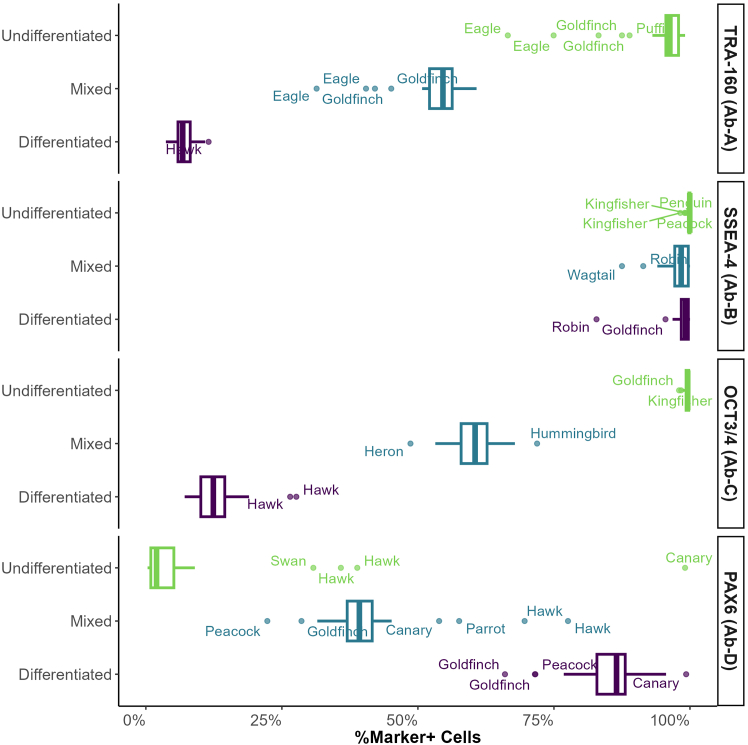
Mean percentage of marker-positive cells by sample type in QAR 2023 Quality Test 2 The mean percentage of cells expressing TRA-1-60, SSEA4, OCT3/4, and PAX6 across undifferentiated (green), mixed (blue), and differentiated (purple) sample types, including standard deviation, are shown. Participant codenames are highlighted for results that deviate most significantly from the median. Data from the participant codenamed “Swallow” have been excluded from this analysis.

The mean percentage of cells positive for each of the four markers, TRA-1-60, SSEA4, OCT3/4, and PAX6, was calculated (Figure 4), excluding the data from the participant “Swallow” due to the large number of outliers observed across all samples. For transparency, calculated values both including and excluding the “Swallow” dataset are reported in Table S7. The most variable marker was PAX6, with standard deviations of 6.2% (differentiated), 9.9% (mixed), and 18.2% (undifferentiated), followed by TRA-1-60 with standard deviations of 1.9% (differentiated), 5.5% (mixed), and 6.7% (undifferentiated); OCT3/4 with 4.4% (differentiated), 4.1% (mixed), and 0.5% (undifferentiated); and SSEA4 with 2.8% (differentiated), 2.6% (mixed), and 0.4% (undifferentiated).

As shown in Figures 4 and S4, most participants’ data show strong agreement across markers, with only minor exceptions. For the PAX6 marker, a few outliers appear in the undifferentiated and mixed samples, primarily from participant “Hawk.” Similarly, for TRA-1-60 in the undifferentiated and mixed samples, outliers originate from participants “Eagle” and “Goldfinch.” These outliers are evident when considering the entire dataset. However, as previously shown in Figure 3, individual data from “Hawk,” “Eagle,” and “Goldfinch” still exhibit clear, proportional shifts in the percentage of marker-positive cells from the undifferentiated to mixed and from mixed to differentiated samples, indicating that flow cytometry results remained consistent and internally coherent for these participants.

The marker SSEA4 was used by 18/22 (82%) participants in Quality Test 1 and was also included as a marker in Quality Test 2. Notably, the level of SSEA4 expression remained high across all analyzed samples. While SSEA4 is one of the markers commonly recommended for inclusion in iPSC QC panels based on expert consensus, and given its widespread use among participants, SSEA4 remains a relevant marker for further investigation. Therefore, a Validation Round was performed to examine marker expressions in greater detail.

#### Validation Round 2024

**Figure 5:**
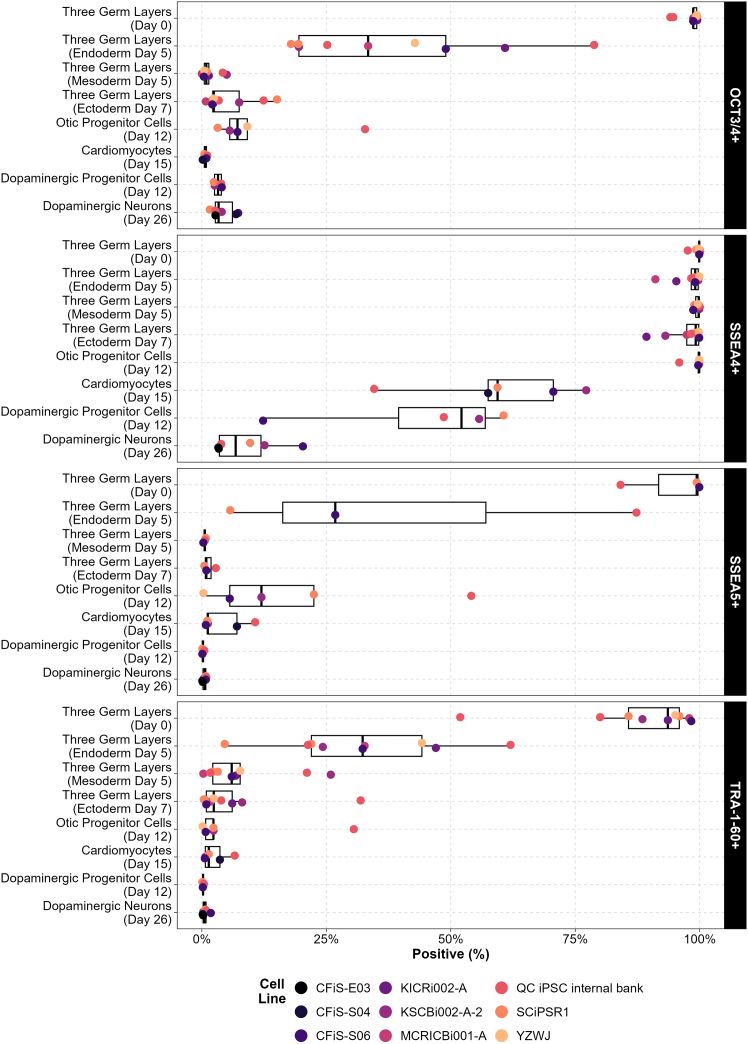
Marker-positive cell percentages across differentiated iPSC lines in Validation Round 2024 Average percentages of cells positive for TRA-1-60, SSEA4, OCT3/4, and SSEA5 are shown for five iPSC lines differentiated into three germ layers, endoderm (day 5), mesoderm (day 5), and ectoderm (day 7), and into specific cell types, otic progenitor cells (day 12), cardiomyocytes (day 15), dopaminergic progenitor cells (day 12), and dopaminergic neurons (day 26). Bars represent mean percentages ±SD.

Building on the findings from QAR 2023, a Validation Round 2024 was carried out to assess the expression of SSEA4, OCT3/4, TRA-1-60, and SSEA5 in five different iPSC lines differentiated into three germ layers (Figure 5). Cell lines used were provided by five different participants. SSEA4 expression remained high, approaching 100% expression, in all five cell lines at day 0, day 5 endoderm, day 5 mesoderm, and day 7 ectoderm. Consistent with previous findings, OCT3/4 and TRA-1-60 expressions declined rapidly in comparison to SSEA4, with levels decreasing from day 5 of differentiation across all cell lines tested in the Validation Round. SSEA5 expression also showed decreased levels from day 5 of differentiation, with 0% expression observed in the day 5 mesoderm layer and day 7 ectoderm layer samples. In the day 5 endoderm samples, OCT3/4, TRA-1-60, and SSEA5 expression was slightly higher than in other lineages, likely due to the lower efficiency of endoderm differentiation, yet still markedly lower than SSEA4. The reduced endoderm differentiation efficiency in this experiment is supported by lineage-specific marker expression in Figure S5.

To assess whether expression levels of SSEA4 decline over time, we applied long-term differentiation protocols toward otic progenitor cells (12 days), cardiomyocytes (15 days), dopaminergic progenitor cells (12 days), and dopaminergic neurons (26 days). These cell types were selected based on established in-house protocols routinely used within our laboratories. Validation of cell type-specific marker expression is provided in Figure S5.

As shown in Figure 5, a progressive decrease in SSEA4 expression was observed across most cell types over time, with some variability between cell lines. Mean SSEA4 expression levels ranged approximately between 50% and 60% for cardiomyocyte and dopaminergic progenitor cell differentiations. In dopaminergic neurons, expression was notably lower, with a mean below 10%. In contrast, otic progenitor cells maintained elevated SSEA4 expression levels even after 12 days of differentiation.

Expressions of TRA-1-60, OCT3/4, and SSEA5 remained consistently low, typically between 0 and 10%, across all long-term differentiation conditions. The exception was observed in otic progenitor cells, where OCT3/4 and SSEA5 showed slightly elevated levels, with mean expression values around 10%, with OCT3/4 remaining just below and SSEA5 slightly above this value.

## Discussion

This international benchmarking initiative represents a significant step toward resolving a key challenge in regenerative medicine: the lack of harmonized standards for iPSC QC. Through two coordinated QARs (QAR 2019 and QAR 2023), involving 18 and 23 participating sites, respectively, across 12 countries and five continents, we engaged a diverse range of institutions, including academic centers, biotechnology companies, government-funded iPSC facilities, and medical research charities. By evaluating inter-laboratory consistency in flow cytometry-based identity assays and qPCR-based genomic integrity assessments, this study provides the first global, multi-site dataset of empirical evidence for reproducible iPSC QC. Our findings highlight markers and workflows that deliver the most consistent results, offering a practical foundation for standardization and global interoperability.

### Genetic testing across the network

In QAR 2019, the qPCR kit and accompanying analysis software demonstrated high-quality, largely consistent data. Some variability in procedures and occasional analysis errors was observed, underscoring the need for appropriate controls and replicates. Both the kit and the software were specifically designed and developed as a diagnostic for fast and simple in-house testing of hPSC cultures. The kit manufacturer reports that the assay can detect culture abnormalities that are present in at least 30% of the population, similar to the sensitivity of other genetic testing methods (Baker et al., 2016). This qPCR-based method targets nine genetic regions, eight of which are known regions of recurrent karyotypic abnormalities in hPSC cultures (Baker et al., 2016). It was not, however, designed to replace more extensive karyotypic analysis, and the need for more informative methods for assessing genetic integrity remains an active area of discussion (Jo et al., 2020). At present, no single approach can provide full identification of all abnormalities that may arise during reprogramming or extended culture of iPSCs (O'Shea et al., 2020). Therefore, it is strongly encouraged to combine G-banding karyotype with a molecular method such as array comparative genomic hybridization or SNP array and WGS.

### Flow cytometry analysis across the network

Flow cytometry characterizations in QAR 2019, using participants’ standard protocols, revealed substantial variability due to diverse reagents, staining combinations, and instrumentation. Co-staining with OCT3/4 and TRA-1-60 was the most common and reliable combination, although sample preparation issues led to some failed analyses. To address these limitations, workshops were held prior to QAR 2023 to harmonize protocols, and a GO/NO-GO step was introduced to exclude samples with insufficient cell numbers or events.

In QAR 2023, two testing formats were applied: Quality Test 1 used participants’ own protocols, while Quality Test 2 employed standardized samples, reagents, and harmonized procedures. In Quality Test 1, 19/22 (86.4%) participants successfully distinguished the three sample types (undifferentiated iPSCs, spontaneously differentiated iPSCs, and a 1:1 mixture). Collectively, OCT3/4, TRA-1-60, and SSEA5 emerged as the top markers, showing both low inter-participant variability and clear proportional expression changes across sample types, underscoring their suitability for assessing differentiation status. Notably, an even higher proportion of participants (22/23, 96%) correctly distinguished the sample types in the standardized Quality Test 2, illustrating the benefit of harmonization. Only one participant generated inconsistent results, most likely due to prolonged shipment times, which may have compromised sample integrity. Minor outliers in the TRA-1-60 and PAX6 datasets still followed clear proportional trends across sample types, demonstrating that accurate classification remained feasible despite variations in gating. Although refined gating could further improve inter-participant alignment, the existing settings were sufficient to distinguish the samples, albeit with a narrower dynamic range and reduced sensitivity to differences in marker expression.

Results from QAR 2023 also showed that SSEA4 remained highly expressed across all samples. In the Validation Round 2024, five iPSC lines were assessed for SSEA4, SSEA5, TRA-1-60, and OCT3/4 expression during differentiation into four different cell types (cardiomyocytes, otic progenitors, dopaminergic progenitors, and dopaminergic neurons) as well as into progenitors of all three germ layers. SSEA4 showed the slowest decline, while SSEA5, TRA-1-60, and OCT3/4 were downregulated more rapidly. These results suggest that SSEA4 is more robustly and persistently expressed in iPSCs, making it less suitable for tracking early differentiation events. However, its stability may be advantageous as a sensitive marker for detecting residual pluripotent cells in differentiated products, particularly in QC settings where detection of uncommitted cells is critical.

### Future considerations

This study focused primarily on flow cytometry-based identity assays and qPCR-based genomic integrity screening. Future QARs could extend benchmarking to additional technologies and differentiation contexts, enabling evaluation of functional assays and marker performance across a wider range of cell types. Such efforts would further strengthen the evidence base for reproducible practices and support the development of comprehensive QC frameworks.

Based on insights from QAR 2019, QAR 2023, and the Validation Round 2024, we propose a preliminary roadmap for implementing future multi-site benchmarking of emerging iPSC QC technologies. Key components of such a benchmarking strategy include: (1) establishment of well-defined reference materials matched to the analytical platform (e.g., iPSC lines with predefined genomic or phenotypic characteristics); (2) co-development of harmonized protocols through workshops and pilot testing, incorporating clear GO/NO-GO criteria to minimize procedural variability; and (3) implementation of shared or standardized data analysis pipelines to reduce software-dependent variability (e.g., in the context of flow cytometry, gating-dependent differences between sites).

Incorporation of these elements can shorten the overall timeline of international benchmarking initiatives by reducing common failure modes, enabling earlier detection of workflow deviations, and streamlining data interpretation across participating sites. To support future efforts, we outline below a proposed roadmap for international multi-site benchmarking.

1. Preparation phase
  • Selection and pre-validation of reference materials
  • Definition of assay scope, test parameters, and performance metrics
2. Harmonization phase
  • Cross-site workshops and protocol alignment
  • Pilot testing to identify sources of variability
  • Definition of GO/NO-GO acceptance criteria
3. Benchmarking phase
  • Distribution of blinded samples
  • Multi-site testing using agreed workflows
  • Standardized data capture and reporting formats
4. Analysis and feedback phase
  • Centralized or harmonized data analysis
  • Inter-site performance comparison
  • Identification of potential sources of variability and protocol refinement

A critical next step for enabling global adoption of harmonized iPSC QC practices is engagement with regulatory authorities. While this study provides empirical evidence to benchmark assay reproducibility and identify robust markers, translating these findings into accepted standards will require coordinated dialog with regulators. International organizations such as GAiT, the International Society for Stem Cell Research, and the International Society for Cell & Gene Therapy are actively engaged in developing standards and guidelines for pluripotent stem cell therapies. Building on this role, they are well positioned to lead regulatory engagement by leveraging global networks and collaborative platforms.

### Conclusion

Despite two decades of iPSC research, harmonized QC standards remain limited, hindering the scalable manufacture of iPSC-derived ATMPs. This global study integrates standardized assays with empirical benchmarking across multiple institutes to generate data-driven evidence for reproducible QC protocols and reliable marker panels. Our findings support the inclusion of OCT3/4, TRA-1-60, and SSEA5 as robust markers for monitoring the undifferentiated status of iPSCs, together with harmonized flow cytometry workflows that apply clear GO/NO-GO thresholds and validated gating strategies to minimize subjectivity. For genomic integrity, we recommend combining G-banding karyotype with genome-wide molecular assays, while using qPCR as a complementary tool for routine screening. This multi-tiered approach balances accuracy, scalability, and feasibility for global implementation.

Standardization is more than a technical exercise; it reduces resource burden, aligns regulations, and enables scalable, interoperable workflows. Sustained international collaboration and continued coordinated efforts will be essential to advance shared quality standards, strengthen supply chains, and deliver high-quality, affordable iPSC-derived therapies globally.

## Methods

### Quality Assessment Round survey

Participants in each study were provided with a survey to document and better understand their routine iPSC testing practices. Complete QAR 2019 and QAR 2023 questionnaires are provided in Document S1. The survey included questions covering the donor management process, infectious agent testing, sterility testing, genotyping, phenotyping, characterization, and safety testing. Participants were also asked to indicate which procedures were governed by SOPs and whether testing was conducted in-house or outsourced to external laboratories. The qualitative data were collated and summarized as the percentage of total responses to each question.

### Quality Assessment Round design

**Figure 6:**
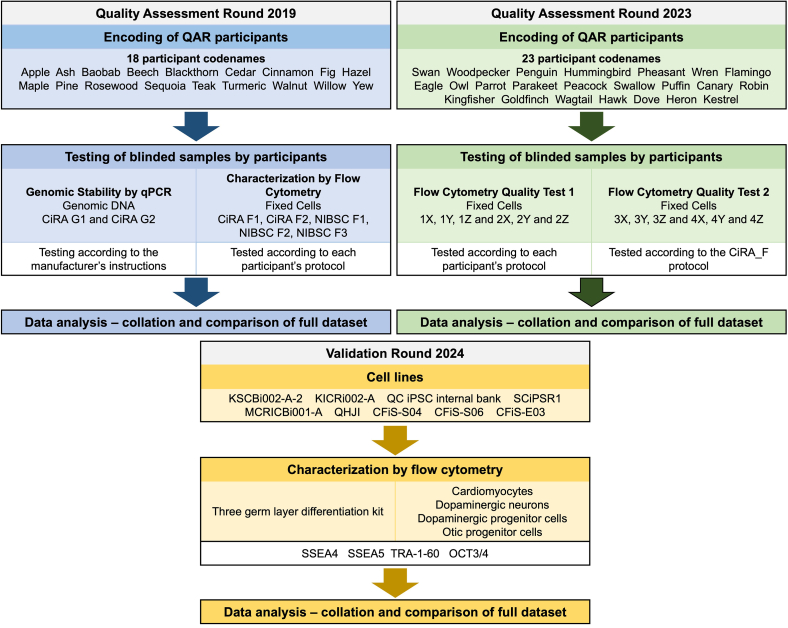
QAR design flow diagram The main stages of each QAR and the Validation Round, including lists of the participants’ codenames.

QAR 2019 (Figure 6): 18 participants were asked to assess the genetic integrity of two genomic DNA samples (CiRA G1 and CiRA G2; provided by CiRA_F) using a commercially available qPCR kit. In parallel, participants analyzed five fixed cell samples (CiRA F1, CiRA F2, NIBSC F1, NIBSC F2, and NIBSC F3; provided by CiRA_F and NIBSC) by flow cytometry, applying their established laboratory protocols and routinely used antibodies to confirm an undifferentiated, iPSC phenotype. Each participant independently analyzed their own datasets, submitting pseudonymized results to GAiT under a unique codename. GAiT subsequently collated and analyzed the complete QAR 2019 dataset.

QAR 2023 (Figure 6): Participants were asked to analyze an identical set of samples provided by CiRA_F using two distinct flow cytometry quality tests. For Quality Test 1, 22 participants tested fixed cell samples (1X, 1Y, and 1Z and 2X, 2Y, and 2Z) following their own laboratory protocols and employing the antibodies routinely used to verify a differentiated, iPSC phenotype. Each participant analyzed the data independently, according to their own procedures and software tools. For Quality Test 2, 23 participants tested fixed cell samples (3X, 3Y, and 3Z and 4X, 4Y, and 4Z) using a standardized protocol and antibody panel provided by CiRA_F. Each participant analyzed the data independently according to the supplied instructions but using their own analysis software. Pseudonymized results were submitted to GAiT under assigned codenames. GAiT subsequently collated and analyzed the complete QAR 2023 dataset.

Validation Round 2024 (Figure 6): Five participants submitted cell lines to CiRA_F for a focused validation round comparing the expression of SSEA4, SSEA5, TRA-1-60, and OCT3/4.

### Quality Assessment Round and Validation Round samples and reagents

Detailed information on the samples and reagents used in QAR 2019, QAR 2023, and Validation Round 2024 is provided in Document S1. Document S1 also contains the full instructions distributed to participants for QAR 2019 and QAR 2023. The use of human iPSC lines in this study was approved by the Kyoto University Graduate School and Faculty of Medicine Ethics Committee, approval number R3218.

### Quality Assessment Round testing and analysis

#### QAR 2019

QAR 2019 genomic stability by qPCR: Samples were thawed and tested using the hPSC genetic analysis kit (STEMCELL Technologies, Cat. No. 07550). Individual datasets were analyzed by participants using the associated data analysis software (STEMCELL Technologies hPSC Genetic Analysis Tool v.1.0.1: 2018-11-13).

QAR 2019 characterization by flow cytometry: Participants were required to test the supplied flow cytometry samples with a minimum of two markers from the standard undifferentiated panel (OCT3/4, TRA-1-60, TRA-1-81, SSEA3, SSEA4, SOX2, or NANOG) as they normally would as part of their in-house iPSC QC testing programs. A combination of at least one intracellular (e.g., OCT3/4, SOX2, or NANOG) and one extracellular (e.g., SSEA4 or TRA-1-60, TRA-1-81) marker was also required. Analysis for additional markers, which comprise participant’s routine iPSC quality testing, were also to be shared. When performing nuclear staining, participants were asked to also use their own standard permeabilization protocols. Participants analyzed their own individual dataset using their internal procedures and provided results as the percentage of cells positive for each marker tested.

#### QAR 2023

QAR 2023 flow cytometry Quality Test 1: Participants were required to test the supplied cell samples (1X, 1Y, and 1Z and 2X, 2Y, and 2Z) with a minimum of two markers from the standard undifferentiated panel (OCT3/4, TRA-1-60, TRA-1-81, SSEA3, SSEA4, SOX2, or NANOG) as they normally would as part of their in-house iPSC QC testing programs. A combination of at least one intracellular (e.g., OCT3/4, SOX2, or NANOG) and one extracellular (e.g., SSEA4, TRA-1-60, or TRA-1-81) marker was also required. Analysis for additional markers, which comprise participant’s routine iPSC quality testing, were also to be shared. When performing nuclear staining, participants were asked to also use their own standard permeabilization protocols. Participants analyzed their own individual dataset using their internal procedures and provided results as the percentage of cells positive for each marker tested.

QAR 2023 flow cytometry Quality Test 2: Participants were required to test samples (3X, 3Y, and 3Z and 4X, 4Y, and 4Z) using the provided single staining protocol and reagents. Cells were thawed, added to 5 mL of buffer, and divided into five individual tubes. Cells were centrifuged at 200 × *g* for 5 min and resuspended in 30 μL of Ab-A, Ab-B, Ab-C, Ab-D, or Ab-U (buffer only). Cells were incubated in the dark at room temperature for 15 min, mixed, and then incubated for a further 15 min. 1 mL of buffer was added to each tube, and cells were centrifuged at 200 × *g* for 5 min. Cells were resuspended in 250 μL of buffer, filtered through a nylon mesh strainer, and stored at 4°C in the dark until analysis. Acquisition instructions included the removal of doublet populations and analysis of cells positive for FITC using the unstained sample as the negative control. Results were provided as the percentage of cells positive for each marker tested.

### Data analysis

Participants submitted their individual analyzed datasets to GAiT. These datasets were collated by quality round, and the results were compared across all participants. Data were managed and shared in accordance with FAIR and CARE principles to ensure accessibility, interoperability, and ethical use. In QAR 2019, participants were assigned codenames based on tree species, and in QAR 2023, bird species. These distinctive labels enabled each organization to recognize its own results without compromising anonymity and reduced confusion compared with random alphanumeric identifiers, particularly during follow-up discussions and comparative analyses. For clarity and continuity, these codenames are retained in this manuscript.

## Resource availability

### Lead contact

Requests for further information and resources should be directed to and will be fulfilled by the lead contact, Anna Falk (anna.falk@med.lu.se).

### Materials availability

This study did not generate new unique reagents. All iPSC lines used in QAR 2019 and QAR 2023 were pre-existing lines established at CiRA_F. Experimental samples, including spontaneously differentiated and fixed cell preparations, were generated solely for inter-laboratory assessment and are not maintained as distributable reagents.

For Validation Round 2024, five participating institutions submitted pre-existing iPSC lines to CiRA_F for centralized analysis. In accordance with institutional agreements and ownership by the submitting institutions, these Validation Round 2024 cell lines are not available for redistribution.

All instructions, workflows and survey materials used in the QARs are provided in Document S1.

### Data and code availability

All flow cytometry data, qPCR data, and survey-derived metadata reported in this study will be shared by the lead contact upon request. This study does not include any data types requiring deposition in a specialized repository, and it does not report original code. Any additional information required to reanalyze the data reported in this paper is available from the lead contact upon request.

## Acknowledgments

Funding for the QAR 2023 and Validation Round 2024 was provided by the 10.13039/501100000371Circulation Foundation through a grant from the 10.13039/501100003478Japanese Ministry of Health, Labour and Welfare (MHLW). This work was supported by 10.13039/501100003653Korea National Institute of Health project 2020-NG-016-00 (H.Y.C.), Vinnova Innovation Milieus (IndiCell 2021-02695 [A.F.] and StartCell 2024-01124 [A.F.]) funded by the Swedish Agency for Innovation, the Lund Stem Cell Center (A.F.), LU ATMP Centrum (A.F.), SFO StemTherapy (A.F.), and the National ATMP Research School, funded by the 10.13039/501100004359Swedish Research Council (A.H.). The authors thank Marie Jönsson for support with the graphical abstract and acknowledge the Cell and Gene Technology Core and the FACS Core at the Lund Stem Cell Center.

## Author contributions

Conceptualization, A.L.M., J.B., J.M., S. Sullivan, and M.T.; methodology, A.L.M., J.B., A.G., J.M., A.J.H., S. Sullivan, and M.T.; formal analysis, D.M. and A.J.H.; investigation, S. Sakamoto, A.H., K.A., E.A., B.A., R.B., R.A.Q.B., A.B.C., D.C., H.Y.C., M.C., B.A.C., S.C., S.J.D., N.E., X.F., M. Feyeux, M. Forrester, S.M.G., A.J.H., A.L.H., L.G.J., R.N.J., Y.K., T.H.K.-B., J.-H.K., M.K., S.K., B.K., C.Y.L., W.L., M.R.M., M.M., L.M.-A., O.M., S.L.M., H. Munir, S.H.S.N., H. Ogawa, S.O., N.P., L.P.V., I.P.d.S.B., A.R.P., S.Q., J.R., A.R., S. Sullivan, R.S., K.S., S. Selvitella., G. Singh, M.J.K.S., M.S., C.T., P.T., E.U., J.M.A.V., A.V., J.W.V.W., K.W.-C., J.W., A.Y., and G.Z.; resources: A.F., A.H., A.L.M., H.C., S.C., N.E., A.G., H.-Y.H., T.H., J.-H.K., K.O., J.M., and P.T.; writing – original draft, A.H., R.W., A.J.H., and S. Sullivan; writing – review & editing: A.F., A.H., A.L.M., E.A., R.B., J.B., D.C., S.J.D., N.E., M. Forrester, J.-H.K., M.M., L.M., S.H.S.N., J.S., G. Stacey, S. Sullivan, M.T., and P.T.; supervision, A.L.M., A.B.G., A.C.C.d.C., A.F., H.C., H.Y.C., B.A.C., R.N.J., H.K., A.M., J.M., H. Ohara, A.R., M.J.K.S., and G. Stacey; project administration, H. Mamiya, R.W., and S. Sullivan; funding acquisition, A.L.M. and T.H.

## Declaration of interests

H.C., M.R.M., O.M., and X.F. are employed by the Center for Commercialization of Regenerative Medicine (CCRM). G.Z. was formerly employed by CCRM. A.F. is the CSO of CCRM Nordic. R.B. is employed by SmartCella. A.R.P., D.M., J.B., M.J.K.S., S.K., and W.L. are employed by Cell and Gene Therapy Catapult. E.A. is the CSO of ViSync. S.J.D. is employed by FUJIFILM Cellular Dynamics Inc. K.S., L.M.-A., and S.M.G. are employed by TreeFrog Therapeutics. M. Feyeux is a co-founder, shareholder, member of the Strategic Committee, and employee of TreeFrog Therapeutics. A.G., A.J.H., A.L.H., J.W.V.W., M. Forrester, and R.N.J. are employed by STEMCELL Technologies. J.R. is employed by BlueRock Therapeutics. L.G.J. was employed by BlueRock Therapeutics (2019) and Notch Therapeutics (2023/2024). C.Y.L. is employed by Century Therapeutics. A.M. is employed by Dark Horse Consulting Group. M.M. is and S.L.M. was employed by Cellino, S.L.M. is listed on patents 12195765 B2, US 11931737 B2, and US11708563 B2. S.H.S.N. was employed by Notch Therapeutics and is currently an independent consultant. H. Ohara. and S. Sakamoto are employed by Sumitomo Pharma Co., Ltd. S. Sullivan is employed by Lindville Bio. E.U. is employed by BioLamnia. J.S. is a founder of iPS Bio, Inc.

## Supplemental information

Document S1. Figures S1–S5, Tables S1–S12, and Methods S1–S4

Document S2. Article plus supplemental information
