# Supplementary material for: Toward standardized iPSC testing: Insights from a multi-year international Quality Assessment Round
Source: Stem Cell Reports. 2026 Mar 19;21(4):102857. doi: 10.1016/j.stemcr.2026.102857 (PMC13083784; doi:10.1016/j.stemcr.2026.102857)
Supplement: Document S1. Figures S1–S5, Tables S1–S12, and Methods S1–S4 [file mmc1.pdf]

Stem Cell Reports, Volume *21*

Supplemental Information

Toward standardized iPSC testing: Insights from a multi-year international Quality Assessment Round

Alice Hägg, Rachel Wood, Ayako L. Mochizuki, Keren Abberton, Elsa Abranches, Belén Alvarez-Palomo, Ricardo Baptista, Raiana Andrade Quintanilha Barbosa, Jacqueline Barry, Adriana Bastos Carvalho, Annelise Bennaceur Griscelli, Antonio Carlos Campos de Carvalho, Diana Chaker, Hong Chang, Hye Young Choi, Margarita Codinach, Begoña Arán Corbella, Scott Cowan, Sarah Jane Dickerson, Ngaire Elwood, Xueling Fan, Maxime Feyeux, Maddy Forrester, Andrew Gaffney, Solenn M. Guilbert, Hye-Yeong Ha, Adam J. Hirst, Arwen L. Hunter, Leanne G. Jamieson, Robert N. Judson, Yonehiro Kanemura, Tais Hanae Kasai-Brunswick, Jung-Hyun Kim, Howard Kim, Manisha Kintali, Siddharth Krishnan, Bernd Kuebler, Chui Yu Lau, Wilson Li, Amanda Mack, Michael R. MacLeod, Marinna Madrid, Hiroaki Mamiya, Lucie Manache-Alberici, Dragoş Mărginean, Olivier Mentre, Stefanie L. Morgan, Joanne Mountford, Humayun Munir, Siemon H.S. Ng, Haruna Ogawa, Steve Oh, Hidetaka Ohara, Keiko Oono, Niall Park, Lygia V. Pereira, Izabella Pereira da Silva Bezerra, Alexandru Robert Podovei, Sergio Querol, Jainy Raje, Angel Raya, Satoko Sakamoto, Raquel Sarafian, Kathleen Schmit, Silvia Selvitella, Gurbind Singh, Matthew J.K. Smart, Jihwan Song, Glyn Stacey, Stephen Sullivan, Miho Sumida, Cecile Terrenoire, Pei Tian, Elias Uhlin, José M.A. Vaquero, Anna Veiga, Jar Wei Vicky Wang, Katherine Warre-Cornish, Jamie Wood, Atsuyo Yamamoto, Gaojun Zhang, Takafusa Hikichi, Marc Turner, and Anna Falk

Supplemental Information

Supplemental Figures

Figure S1. QAR 2019 flow cytometry analysis of marker expression across sample types.

Figure S2. QAR 2023 Quality Test 1 flow cytometry analysis of marker expression across sample types.

Figure S3. QAR 2023 Quality Test 2 flow cytometry analysis of marker expression across sample types.

Figure S4. Distribution of marker-specific data in QAR 2023 Quality Test 2 across different sample types, related to Figure 4.

Figure S5. Validation of cell type-specific marker expression in Validation Round 2024, related to Figure 5.

Supplemental Tables

Table S1. Number of replicate measurements performed by each participant for each sample in QAR 2023 Quality Test 1.

Table S2. Number of participants using each marker in QAR 2023 Quality Test 1.

Table S3. Flow cytometers used by each participant in QAR 2023.

Table S4. Consistency of marker expression between sample types in QAR 2023 Quality Test 1.

Table S5. Percentage recovery of mixed samples based on expected values in QAR 2023 Quality Test 2, related to Figure 3.

Table S6. Duration of cell line and reagent shipment (in days) for each participant in QAR 2023.

Table S7. The mean percentage of cells positive for TRA-1-60, SSEA4, OCT3/4 and PAX6 across the undifferentiated, mixed and differentiated sample types, related to Figure 4.

Supplemental Experimental Procedures

Supplemental Tables related to Supplemental Experimental Procedures

Table S8. Description of samples provided for QAR 2019 and QAR 2023.

Table S9. Description of cell lines and expression tests in Validation Round 2024.

Table S10. iPSC culture media used in the Validation Round 2024.

Table S11. Cell culture coatings used in the Validation Round 2024.

Table S12. Primary and secondary antibodies used in the Validation Round 2024.

Supplemental Methods

Methods S1. QAR 2019 participant survey

Methods S2. QAR 2023 participant survey

Methods S3. QAR 2019 instructions to participants

Methods S4. QAR 2023 Quality Test 1 and 2 instructions to participants

Supplemental Figures

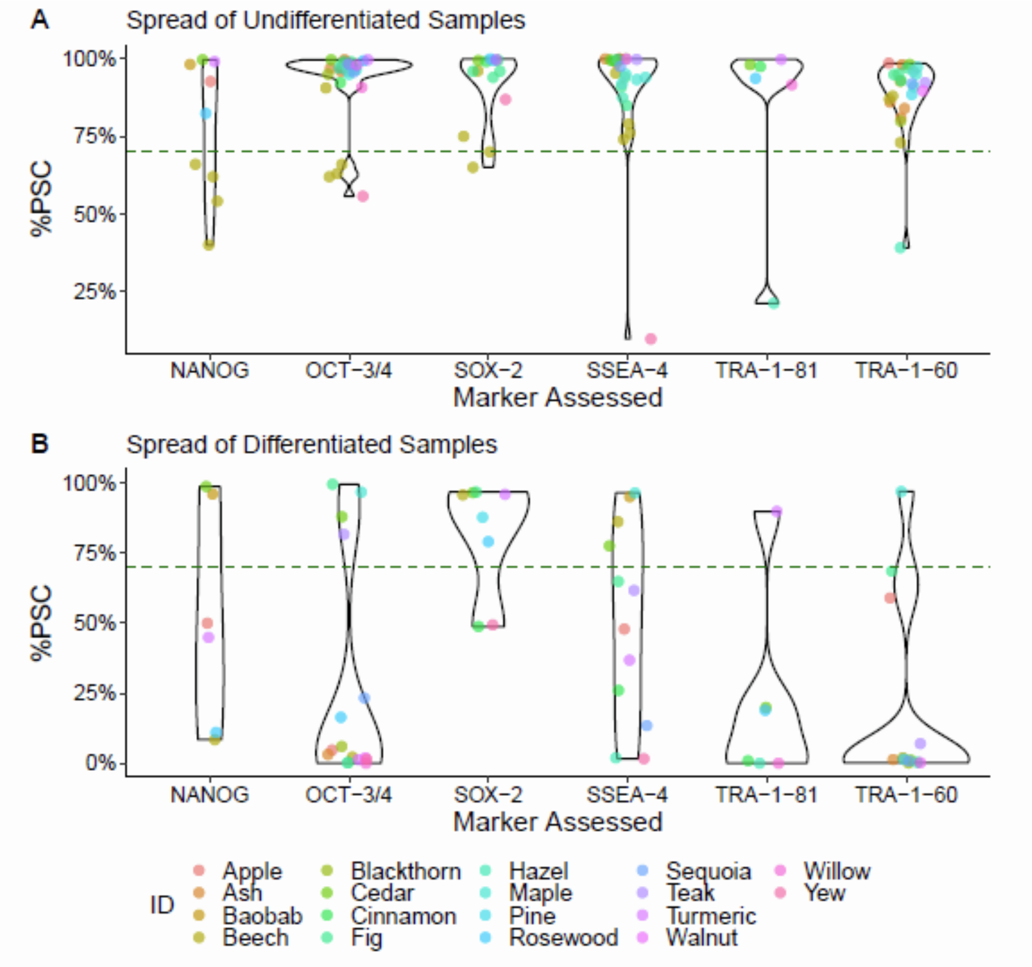

Figure S1. QAR 2019 flow cytometry analysis of marker expression across sample types. Violin plots of all samples that passed the QC criteria. A) shows the data from the undifferentiated CiRA and NIBSC samples, while B) presents data for the spontaneously differentiated CiRA F2 sample. The green dotted line indicates the 70% undifferentiated marker expression threshold, representing the minimum level established in the GAiT QC guidelines (Sullivan et al., 2018). Multiple data points from participants “Beech” and “Fig” reflect repeated analyses performed by these laboratories on the same samples.

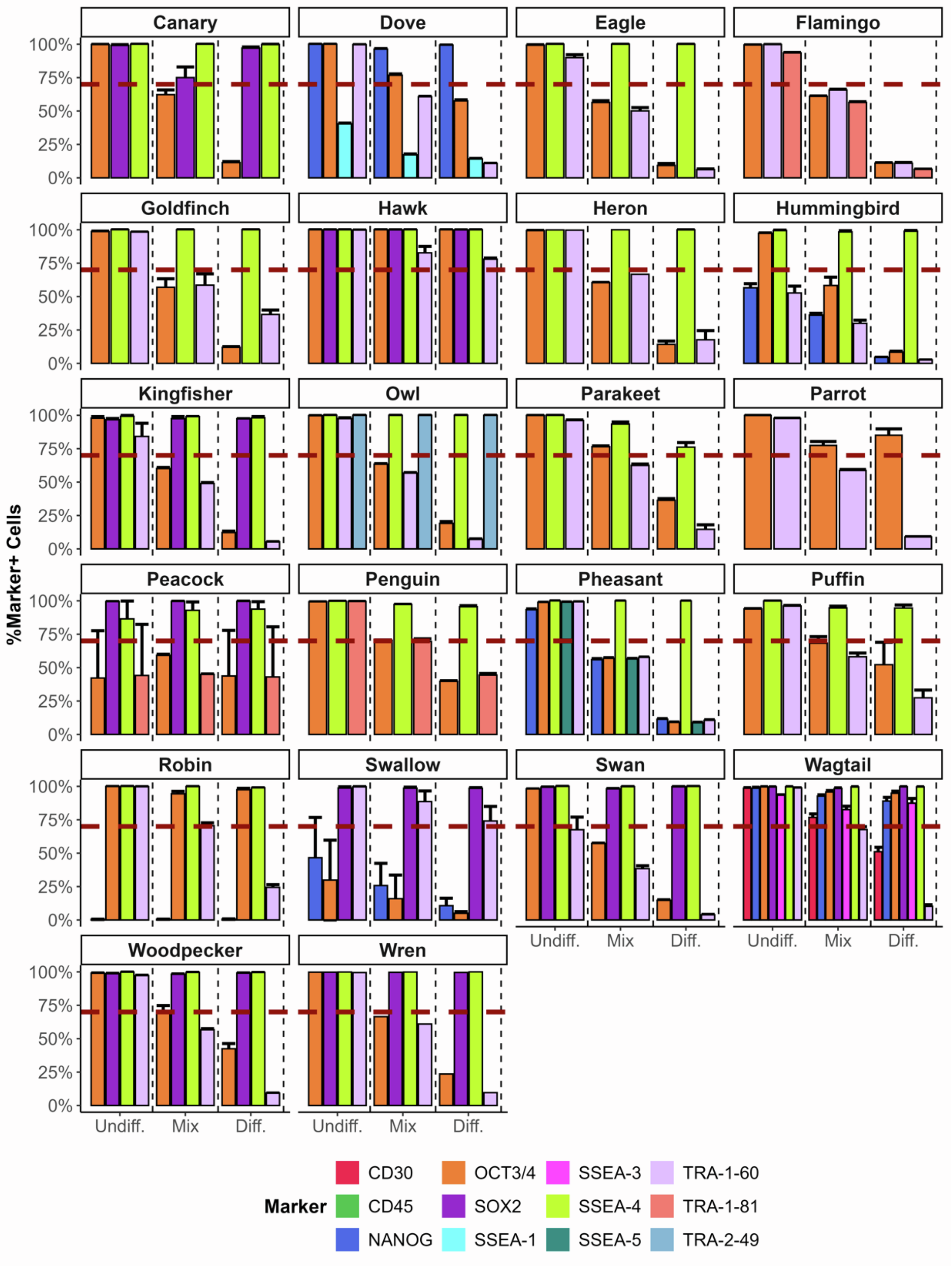

Figure S2. QAR 2023 Quality Test 1 flow cytometry analysis of marker expression across sample types. Percentage of marker-positive cells in each sample type (undifferentiated iPSCs, 1:1 mixed sample, and spontaneously differentiated iPSCs) analysed in Quality Test 1. Results are shown separately for each participant. Each marker is colour coded. Error bars indicate the standard deviation across replicate measurements within each participant's run.

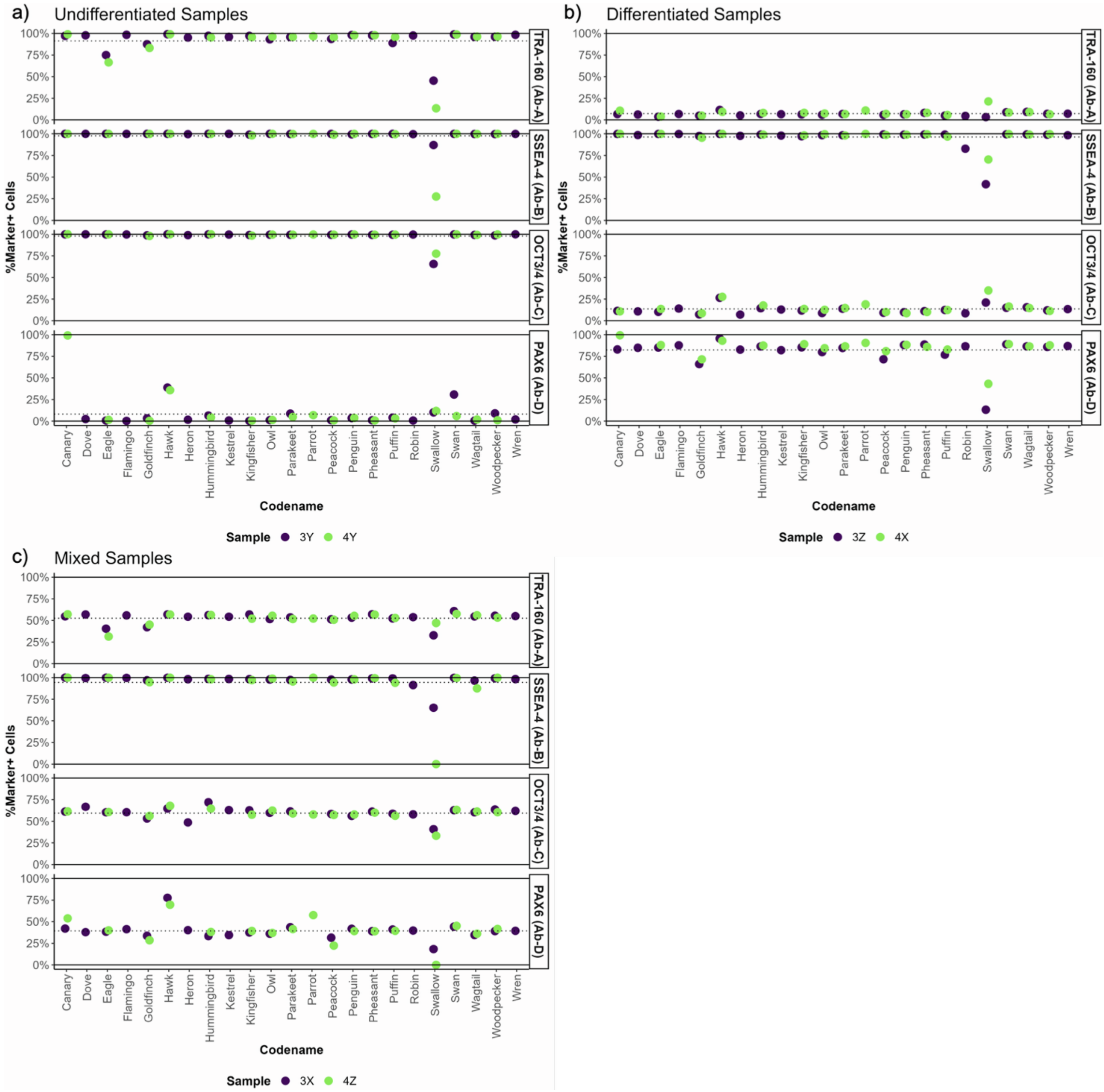

Figure S3. QAR 2023 Quality Test 2 flow cytometry analysis of marker expression across sample types. TRA-1-60, SSEA4, OCT3/4, and PAX6 expression in (a) undifferentiated iPSCs, (b) spontaneously differentiated iPSCs, and (c) a 1:1 mixture of undifferentiated and differentiated cells. Individual points represent each participant's results, and dotted lines indicate the global average for each marker.

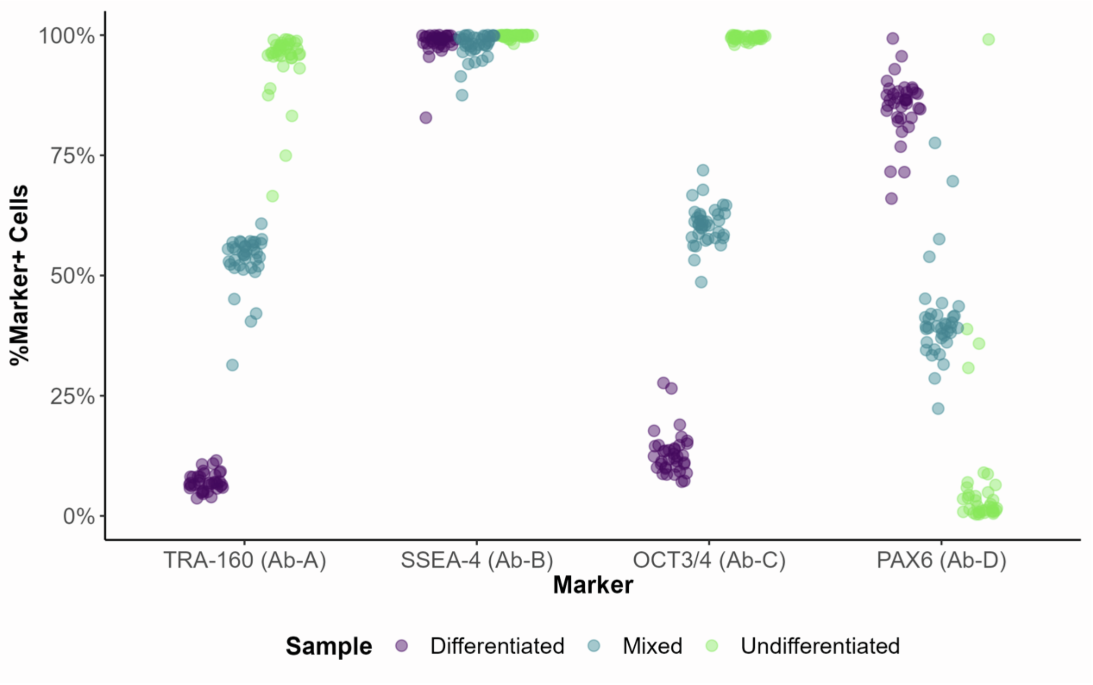

Figure S4. Distribution of marker-specific data in QAR 2023 Quality Test 2 across different sample types, related to Figure 4. Expression levels of TRA-1-60, SSEA4, OCT3/4, and PAX6 were analysed. The different sample types are denoted by following colours: purple for differentiated samples, blue for mixed samples (1:1 ratio), and green for undifferentiated samples. Note that data from the participant identified as "Swallow" have been excluded from this figure for clarity.

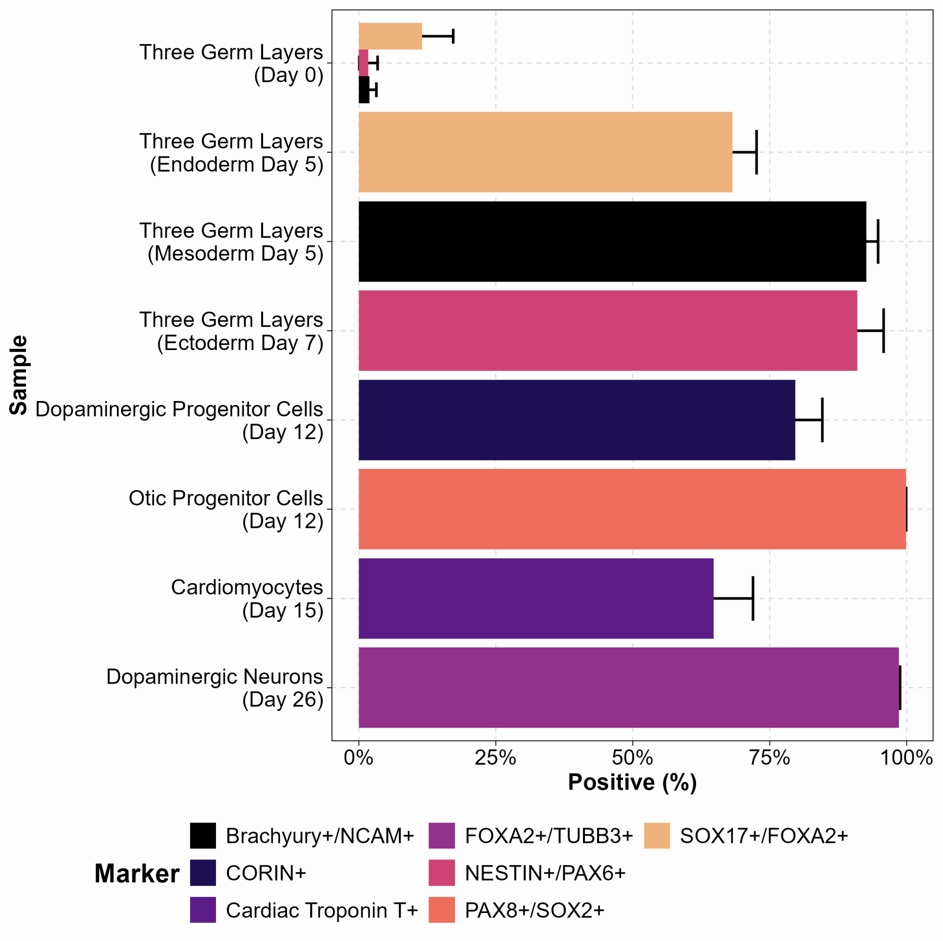

Figure S5. Validation of cell type-specific marker expression in Validation Round 2024, related to Figure 5. Overview of marker expression assessed by flow cytometry during directed differentiation of iPSCs. Samples were collected at key stages: undifferentiated iPSCs (Day 0), endoderm (Day 5), mesoderm (Day 5), ectoderm (Day 7), dopaminergic progenitor cells (Day 12), otic progenitor cells (Day 12), cardiomyocytes (Day 15), and dopaminergic neurons (Day 26).

Supplemental Tables

Table S1. Number of replicate measurements performed by each participant for each sample in QAR 2023 Quality Test 1. Two samples were provided for each condition: undifferentiated (1X, 2Z), differentiated (1Y, 2X), and a 1:1 mixed sample (1Z, 2Y).

	Undifferentiated		Differentiated		Mixed	
**Codename**	**1X**	**2Z**	**1Y**	**2X**	**1Z**	**2Y**
Canary	2	2	2	2	2	2
Dove	3	-	3	-	3	-
Eagle	2	2	2	2	2	2
Flamingo	3	-	3	-	3	-
Goldfinch	1	1	1	1	1	1
Hawk	3	3	3	3	3	3
Heron	1	1	1	1	1	1
Hummingbird	3	3	3	3	3	3
Kingfisher	1	1	1	1	1	1
Owl	2	2	2	2	2	2
Parakeet	2	2	2	2	2	2
Parrot	2	2	2	2	2	2
Peacock	1	1	1	1	1	1
Penguin	1	1	1	1	1	1
Pheasant	3	3	3	3	3	3
Puffin	1	1	1	1	1	1
Robin	3	3	3	3	3	3
Swallow	2	2	2	2	2	2
Swan	1	1	1	1	1	1
Wagtail	3	3	3	3	3	3
Woodpecker	3	3	3	3	3	3
Wren	1	-	1	-	1	-

Table S2. Number of participants using each marker in QAR 2023 Quality Test 1. The table lists all markers together with the participant codenames who reported including them in their in-house flow cytometry panels, as indicated by the green dots. The final row summarizes the total number of participants using each marker.

Codename	OCT3/4	TRA-1-60	SSEA4	SOX2	NANOG	TRA-1-81	SSEA5	TRA-2-49	CD45	CD30	SSEA3	SSEA1
Swan	●	●	●	●	○	○	○	○	○	○	○	○
Woodpecker	●	●	●	●	○	○	○	○	○	○	○	○
Penguin	●	○	●	○	○	●	○	○	○	○	○	○
Hummingbird	●	●	●	○	●	○	○	○	○	○	○	○
Pheasant	●	●	●	○	●	○	●	○	○	○	○	○
Wren	●	●	●	●	○	○	○	○	○	○	○	○
Eagle	●	●	●	○	○	○	○	○	○	○	○	○
Flamingo	●	●	○	○	○	●	○	○	○	○	○	○
Owl	●	●	●	○	○	○	○	●	○	○	○	○
Parrot	●	●	○	○	○	○	○	○	○	○	○	○
Parakeet	●	●	●	○	○	○	○	○	○	○	○	○
Peacock	●	○	●	●	○	●	○	○	○	○	○	○
Swallow	●	●	○	●	●	○	○	○	○	○	○	○
Puffin	●	●	●	○	○	○	○	○	○	○	○	○
Canary	●	○	●	●	○	○	○	○	○	○	○	○
Kingfisher	●	●	●	●	○	○	○	○	○	○	○	○
Robin	●	●	●	○	○	○	○	○	●	○	○	○
Goldfinch	●	●	●	○	○	○	○	○	○	○	○	○
Wagtail	●	●	●	●	●	○	○	○	○	●	●	○
Hawk	●	●	●	●	○	○	○	○	○	○	○	○
Dove	●	●	○	○	●	○	○	○	○	○	○	●
Heron	●	●	●	○	○	○	○	○	○	○	○	○
**Number of Participants (n)**	22	19	18	9	5	3	1	1	1	1	1	1

Table S3. Flow cytometers used by each participant in QAR 2023. The table lists the instrument name, manufacturer, and the participants that reported using each flow cytometer. The final column shows the percentage of all participants using each instrument.

Flow cytometer used	Manufacturer	Codename	Number of participants (n)
BD FACSAria III	BD Biosciences	Goldfinch	1
Attune NxT	Thermo Fisher	Penguin	1
BD Accuri C6	BD Biosciences	Hummingbird<br>Swallow<br>Canary	3
BD Accuri C6+	BD Biosciences	Kestrel	1
BD FACSCanto II	BD Biosciences	Owl<br>Puffin<br>Dove	3
BD FACSLyric	BD Biosciences	Swan	1
BD LSRFortessa	BD Biosciences	Wren<br>Woodpecker	2
CytoFLEX LX	Beckman Coulter	Kingfisher	1
BD FACSVerse	BD Biosciences	Eagle	1
GALLIOS	Beckman Coulter	Peacock	1
Guava easyCyte 12HT	Cytek Biosciences	Parrot	1
BD LSRII	BD Biosciences	Flamingo	1
MACSQuant Analyzer 10	Miltenyi Biotec	Pheasant<br>Parakeet<br>Wagtail	3
MACSQuant Analyzer 16	Miltenyi Biotec	Hawk<br>Robin	2
NAVIOS EX	Beckman Coulter	Heron	1

Table S4. Consistency of marker expression between sample types in QAR 2023 Quality Test 1. Within-sample consistency for NANOG, OCT3/4, TRA-1-60 and TRA-1-81 was calculated by the mean percentage of marker-positive cells and standard deviation.

Marker	Sample type	Mean % marker positive cells	Standard deviation (%)
NANOG	Undifferentiated	79.1	± 25.7
	Mixed	60.0	± 28.7
	Differentiated	38.8	± 41.4
OCT3/4	Undifferentiated	94.3	± 19.4
	Mixed	69.3	± 19.8
	Differentiated	41.8	± 36.4
TRA-1-60	Undifferentiated	93.0	± 14.0
	Mixed	60.7	± 14.8
	Differentiated	20.7	± 23.7
TRA-1-81	Undifferentiated	81.1	± 33.7
	Mixed	56.9	± 10.2
	Differentiated	27.7	± 29.5

Table S5. Percentage recovery of mixed samples based on expected values in QAR 2023 Quality Test 2, related to Figure 3. The expected values are calculated from the results of undifferentiated and differentiated samples. Results within the range of 80% to 125% recovery are highlighted in green, while those below 80 or above 125 are highlighted in red.

Codename	Sample set	PAX6 (Ab-D) (%)	OCT3/4 (Ab-C) (%)	TRA-1-60 (Ab-A) (%)	SSEA4 (Ab-B) (%)
Canary	3	0.00	110.10	105.30	100.10
	4	129.40	277.00	292.00	100.00
Dove	3	86.90	120.50	109.40	100.20
Eagle	3	89.50	109.80	102.90	100.00
	4	89.20	107.00	89.10	100.00
Flamingo	3	93.80	106.40	106.20	99.80
Goldfinch	3	97.00	100.40	91.10	98.00
	4	79.40	105.30	102.30	96.90
Hawk	3	115.40	102.30	102.90	99.90
	4	108.10	106.30	104.90	100.00
Heron	3	95.20	91.70	108.10	99.40
Hummingbird	3	72.00	125.80	107.90	98.90
	4	83.00	109.90	108.90	98.50
Kestrel	3	83.20	111.90	105.90	99.40
Kingfisher	3	87.50	113.10	110.10	100.00
	4	87.40	102.80	100.00	98.80
Owl	3	89.00	110.40	104.20	98.60
	4	86.30	111.30	107.00	99.00
Parakeet	3	93.50	108.60	104.50	98.10
	4	90.50	103.40	100.60	96.50
Parrot	4	118.20	97.50	96.90	100.00
Peacock	3	86.60	107.70	103.20	98.00
	4	54.50	104.70	99.10	95.40
Penguin	3	91.20	102.70	101.80	98.50
	4	85.00	106.60	106.20	98.40
Pheasant	3	86.90	111.00	107.90	99.50
	4	89.70	109.60	106.90	99.60
Puffin	3	101.40	105.00	111.40	99.50
	4	91.60	100.60	104.20	95.70
Robin	3	91.00	107.10	105.40	100.20
Swallow	3	156.70	94.20	134.70	101.20
	4	0.00	59.20	270.40	0.00
Swan	3	74.00	109.40	112.90	100.00
	4	95.10	108.80	107.30	99.70
Wagtail	3	79.40	105.20	103.80	96.90
	4	81.60	107.60	106.30	87.90
Woodpecker	3	82.50	115.00	107.70	99.80
	4	93.60	109.00	103.50	100.10
Wren	3	88.60	109.40	104.20	99.00

Table S6. Duration of cell line and reagent shipment (in days) for each participant in QAR 2023.

Codename	Shipment time (days)
Owl	0
Penguin	4
Parrot	3
Eagle	1
Parakeet	4
Hummingbird	5
Woodpecker	8
Wren	5
Peacock	8
Swallow	16
Swan	4
Wagtail	4
Puffin	4
Hawk	7
Canary	4
Dove	5
Flamingo	6
Goldfinch	7
Pheasant	5
Kestrel	1
Kingfisher	5
Heron	6
Robin	6

Table S7. The mean percentage of cells positive for TRA-1-60, SSEA4, OCT3/4 and PAX6 across the undifferentiated, mixed and differentiated sample types, related to Figure 4. Mean and standard deviations are shown in columns 3 and 4. Columns 5 and 6 represent the same calculations with the outlier data set from participant "Swallow" removed.

Sample type	Marker	Mean % marker positive cells	Standard deviation (%)	Mean % marker positive cells (outlier removed)	Standard deviation (%) (outlier removed)
Differentiated	TRA-1-60 (Ab-A)	7.30	3.00	7.10	1.90
	SSEA4 (Ab-B)	96.30	10.40	98.50	2.80
	OCT3/4 (Ab-C)	13.70	5.70	12.90	4.40
	PAX6 (Ab-D)	82.30	14.50	85.20	6.20
Mixed	TRA-1-60 (Ab-A)	52.50	6.30	53.20	5.50
	SSEA4 (Ab-B)	94.50	16.60	97.80	2.60
	OCT3/4 (Ab-C)	59.30	6.60	60.50	4.10
	PAX6 (Ab-D)	39.30	12.20	40.90	9.90
Undifferentiated	TRA-1-60 (Ab-A)	91.30	16.40	94.60	6.70
	SSEA4 (Ab-B)	97.60	11.70	99.80	0.40
	OCT3/4 (Ab-C)	98.00	6.40	99.40	0.50
	PAX6 (Ab-D)	8.20	17.70	8.10	18.20

Supplemental Experimental Procedures

All quality round samples were labelled in a format to remove bias from participant's testing and analysis (Table S8), sample information was released to participants at the completion of each Quality Round.

Table S8. Description of samples provided for QAR 2019 and QAR 2023.

**QAR 2019 sample label**	
CiRA G1	Genomic DNA from a Ff-I03 iPSC clone for which no common abnormality
CiRA G2	Genomic DNA extracted from Ff-I13 iPSC clone which tested positive for a Chr 1q abnormality
CiRA F1	Undifferentiated and self-renewing Ff-I01s04 iPSC line
CiRA F2	Spontaneously differentiated iPSC culture: differentiated and predominantly non-self-renewing cells Ff-I01s04DIFF (cells differentiated from Ff-I01s04 iPSC line by 10 days culture in the medium without FGF2)
NIBSC F1	Undifferentiated, self-renewing NIBSC8 iPSC line (1st technical replicate)
NIBSC F2	Undifferentiated, self-renewing NIBSC8 iPSC line (2nd technical replicate)
NIBSC F3	Undifferentiated, self-renewing NIBSC8 iPSC line (3rd technical replicate)
**QAR 2023 sample label**	
1X, 2Z, 3Y, 4Y	Fixed undifferentiated cells
1Y, 2X, 3Z, 4X	Fixed differentiated cells
1Z, 2Y, 3X, 4Z	Fixed 1:1 ratio mixture of undifferentiated and differentiated cells

QAR 2019 cell samples

iPSC lines (Ff-I03, Ff-I13 and Ff-I01s04) were established from healthy human peripheral blood mononuclear cells (PBMCs) with episomal vectors in CiRA_F. iPSCs were cultured on Laminin-511 E8 fragment coated plates in StemFit (Ajinomoto, AK03N) under 5% CO2 at 37°C. To introduce spontaneous cell differentiation, iPSCs were cultured in AK03N without reagent C for 10 days. All cell samples were fixed with fixation buffer (BD Biosciences, Cat. No. 554655). Fixed cells were cryopreserved in STEM-CELLBANKER® GMP grade (Zenogen Pharma, Cat. No. 11924) and stored in the vapor phase of a liquid nitrogen tank. Samples were distributed using a dry shipper with continuous temperature monitoring. Clonal chromosomal aberration of CiRA G2 (Ff-I13) was confirmed by Karyotyping.

QAR 2023 cell samples

The iPSC line QHJI was derived from healthy human PBMCs and established using episomal vectors in CiRA_F. iPSCs were maintained on Laminin-511 E8 fragment coated plates in StemFit® AK03N medium (Ajinomoto) under 5% CO_2 at 37 °C. For spontaneous cell differentiation, iPSCs were cultured in AK03N medium without reagent C for 10 days. All cell samples were fixed using Cytofix Fixation Buffer (BD Biosciences, Cat. No. 554655). Sample labeling was randomised for participant testing. Fixed cells were cryopreserved in STEM-CELLBANKER® GMP grade (Zenogen Pharma, Cat. No. 11924) and stored in the vapor phase of a liquid nitrogen tank. Samples were distributed using a dry shipper with continuous temperature monitoring.

QAR 2023 flow cytometry reagents supplied to participants

Participants were provided with a flow cytometry buffer (Perm/Wash™ Buffer, BD Biosciences, Cat. No. 554723) and four antibodies: Ab-A, Alexa Fluor® 488 Mouse anti-Human TRA-1-60 (BD Biosciences, Cat. No. 560173); Ab-B, SSEA4 Antibody, anti-Human, FITC, REAfinity® (Miltenyi Biotec, Cat. No. 130-122-918); Ab-C, Alexa Fluor® 488 Mouse anti-Oct3/4 (BD Biosciences, Cat. No. 560253); and Ab-D, Alexa Fluor® 488 Mouse anti-Human PAX6 (BD Biosciences, Cat. No. 561664).

QAR 2019 participant survey and instructions

The complete QAR 2019 participant survey and the experimental instructions are provided in Methods S1 and Methods S3, respectively.

QAR 2023 participant survey and instructions

The complete QAR 2023 participant survey and the experimental instructions are provided in Methods S2 and Methods S4, respectively.

Validation Round 2024 cells and reagents

Table S9. Description of cell lines and expression tests in Validation Round 2024.

Cell line name	Reference	Expression tests
KSCBi002-A-2 (hFSiPS3-1)	Stem Cell Research, Volume 21, 2017, pages 13-15, https://doi.org/10.1016/j.scr.2017.03.009	Three germ layer differentiation<br>Cardiomyocyte differentiation<br>Dopaminergic neuron differentiation<br>Dopaminergic progenitor cell differentiation<br>Otic progenitor cell differentiation
KICRi002-A (CTRL-10-I)	Stem Cell Research, Volume 18, 2017, Pages 22-25,ISSN 1873-5061, https://doi.org/10.1016/j.scr.2016.12.006.	Three germ layer differentiation
QC iPSC internal bank	ATCC #:ACS-1019, Lot #:70030238 expanded in house.	Three germ layer differentiation<br>Cardiomyocyte differentiation<br>Dopaminergic neuron differentiation<br>Dopaminergic progenitor cell differentiation<br>Otic progenitor cell differentiation
SCiPSR1	Episomally reprogrammed CD34 cell. Characterised against GAIT recommendations.	Three germ layer differentiation<br>Cardiomyocyte differentiation<br>Dopaminergic neuron differentiation<br>Dopaminergic progenitor cell differentiation<br>Otic progenitor cell differentiation
MCRIBi001-A (LO2 Cell 17 2.5d HiTemp)	Front. Cell Dev. Biol, Volume 10, 2022, Article 835321, https://doi.org/10.3389/fcell.2022.835321 Research grade iPSC derived from cord blood. Reprogrammed with Sendai Virus.	Three germ layer differentiation
YZWJ	YZWJ was derived from human cord blood and established with episomal vectors in CiRA_F. Culture methods are the same as those for QHJI, in QAR 2023 Cell samples.	Three germ layer differentiation<br>Otic progenitor cell differentiation
CFiS-S06	Research grade iPSC derived from peripheral blood. Reprogrammed with Sendai Virus.	Three germ layer differentiation<br>Cardiomyocyte differentiation<br>Dopaminergic neuron differentiation<br>Dopaminergic progenitor cell differentiation<br>Otic progenitor cell differentiation
CFiS-S04	Research grade iPSC derived from peripheral blood. Reprogrammed with Sendai Virus.	Cardiomyocyte differentiation<br>Dopaminergic neuron differentiation
CFiS-E03	Research grade iPSC derived from peripheral blood. Reprogrammed with episomal vectors.	Dopaminergic neuron differentiation

Three germ layer differentiation

Three germ layer differentiation was performed by using the STEMdiff™ Trilineage Differentiation Kit (STEMCELL Technologies, Cat No. 05230). The procedure followed the manufacturer's protocol. Briefly, iPSCs were plated and cultured for 5 days for endoderm and mesoderm differentiation, and 7 days for ectoderm differentiation, in the appropriate kit medium for inducing germ layer differentiation.

Cardiomyocyte differentiation

iPSCs were transferred to low adherent 6-well plates with aggregation medium (StemPro34 medium supplemented with 2 mM L-glutamine, 50 µg/ml ascorbic acid, 0.4 mM monothioglycerol, 150 µg/ml transferrin, 10 µM Y-27632 and 2 ng/ml BMP4). 24 hours later, EBs were formed and 2× differentiation medium 1 (StemPro34 medium with 2 mM L-glutamine, 50 µg/ml ascorbic acid, 0.4 mM monothioglycerol, 150 µg/ml transferrin, 18 ng/ml BMP4, 12 ng/ml Activin A and 10 ng/ml bFGF) was added to the plate. At day 3, the medium was replaced with Differentiation Medium 2 (StemPro34 medium supplemented with 2 mM L-glutamine, 50 µg/ml ascorbic acid, 0.4 mM monothioglycerol, 150 µg/ml transferrin, 10 ng/ml VEGF, 1 µM IWP-3, SB431542 and Dorsomorphin) and cells were maintained for 4 days. At day 7, the medium was changed to Differentiation Medium 3 (StemPro 34 medium supplemented with 2 mM L-glutamine, 50 µg/ml ascorbic acid, 0.4 mM monothioglycerol, 150 µg/ml transferrin and 10 ng/ml VEGF) and cells were cultured for 8 days. Culture medium was changed every 2-3 days.

Dopaminergic progenitor and neuron differentiation

iPSCs were plated onto iMatrix-coated 24-well plates at 1.0×10^6 cells/well in differentiation media containing Glasgow's minimum essential medium supplemented with 8% KSR, 0.1 mM MEM, 1 mM sodium pyruvate and 0.1 mM 2-mercaptoethanol. The differentiation medium was changed daily. The following were added to the differentiation medium: 100 nM LDN193189 on days 0-12; 500 nM A83-01 on days 0-6; 100 ng mL-1 fibroblast growth factor 8 and 2 µM purmorphamine on days 1-6; and 3 µM CHIR99021 on days 3-12. On day 12, cells were collected and seeded at 2.0×10^4 cells/well in 96-well round-bottom plates in the neural differentiation media containing NB/B27 medium supplemented with GDNF (10 ng/mL), 200 µM ascorbic acid, BDNF (20 ng/mL), dbcAMP (400 µM). Y-27632 (30 µM) was added at the time of seeding to form aggregate spheres. Every 2-3 days, the neural differentiation medium was changed until Day 26.

Otic progenitor cell differentiation

Otic progenitors were induced by plating iPSCs onto 6 well plates and cultured for 12 days in Dulbecco's Modified Eagle Medium: Ham's F12 (DMEM/F12) supplemented with 1× N2 and 1× B27, FGF3 and FGF10 (both growth factors 50 ng/ml).

Table S10. iPSC culture media used in the Validation Round 2024.

iPSC culture medium	Manufacturer
Essential 8™ Medium	Thermo Fisher Scientific
TeSR-E8 medium	STEMCELL technologies
iPS-Brew	Miltenyi Biotec
SFM XF/FF	ATCC
AK03N	Ajinomoto

Table S11. Cell culture coatings used in the Validation Round 2024.

Coating	Manufacturer
Laminin-521	BioLamina
Vitronectin	STEMCELL technologies
CellMatrix Basement Membrane Gel	ATCC
iMatrix-511	Nippi

Validation Round 2024 flow cytometry characterisation

Cells were fixed using Cytofix Fixation Buffer (BD Biosciences, Cat. No. 554655) and permeabilised with Perm/Wash™ Buffer (BD Biosciences, Cat. No. 554723) for intracellular staining. For extracellular antigens, unfixed cells were used. Antibodies targeting pluripotency-associated markers included Alexa Fluor® 488 Mouse anti-Human TRA-1-60 (BD Biosciences, Cat. No. 560173), SSEA4 Antibody, anti-Human, FITC, REAfinity® (Miltenyi Biotec, Cat. No. 130-122-918), Alexa Fluor® 488 Mouse anti-Oct3/4 (BD Biosciences, Cat. No. 560253), and SSEA5 (Miltenyi Biotec, Cat. No. 130-124-907). Primary and secondary antibodies used to assess cell type-specific differentiations are listed in Table S12. Staining was performed in PBS supplemented with 2% FBS for 30 min in the dark. Unstained cells served as negative controls. Samples were analysed using a FACSCanto II flow cytometer and FACS Diva software (BD Biosciences).

Table S12. Primary and secondary antibodies used in the Validation Round 2024. Markers and corresponding antibodies are listed for each differentiation assay, including three germ layer, cardiomyocyte, dopaminergic progenitor, dopaminergic neuron, and otic progenitor differentiation.

Expression tests	Marker	Antibody	Manufacturer	Cat. No.
Three germ layer differentiation	SOX17	Sox17, anti-human, PE	Miltenyi Biotec	130-111-032
	FOXA2	FoxA2, anti-human, APC	Miltenyi Biotec	130-123-850
	Brachyury	Brachyury (T), anti-human, Alexa Fluor 488	Cell Signaling Technology	94663S
	NCAM	CD56 (NCAM), anti-human, APC	STEMCELL Technologies	60021AZ
	NESTIN	NESTIN, anti-human, PE	BioLegend	656806
	PAX6	PAX-6 Antibody, anti-human, APC	Miltenyi Biotec	130-123-267
Cardiomyocyte differentiation	Cardiac Troponin T	Troponin T (Cardiac), monoclonal mouse, anti-human	R&D	MAB1874
	2nd Ab	Goat anti-mouse IgG (H+L) cross-adsorbed secondary antibody, Alexa Fluor 488	Thermo Fisher Scientific	A-11001
Dopaminergic progenitor cell differentiation	CORIN	CORIN, monoclonal mouse, anti-human	SIGMA	WH0010699M1
	2nd Ab	Goat anti-mouse IgG (H+L) cross-adsorbed secondary antibody, Alexa Fluor 488	Thermo Fisher Scientific	A-11001
Dopaminergic neuron differentiation	TUBB3	Tubulin β 3 (TUBB3), anti-human, Alexa Fluor 488	BioLegend	801203
	FOXA	Human HNF-3 beta /FoxA2, polyclonal goat, anti-human	R&D	AF2400
	2nd Ab	Donkey anti-goat IgG (H+L) cross-adsorbed secondary antibody, Alexa Fluor 647	Thermo Fisher Scientific	A-21447
Otic progenitor cell differentiation	SOX2	SOX2, monoclonal, anti-human, CoraLite® Plus 488	Proteintech	CL488-66411
	PAX8	PAX8 polyclonal, anti-human, CoraLite® Plus 647	Proteintech	CL647-10336

Supplemental Methods

Methods S1. QAR 2019 participant survey

iPSC Quality Assessment Round 2019 Participant Survey

iPSC Quality Round 2019 Technical Survey (including 'secondary testing' and 'sample reorder requests)

iPSC Quality Round 2019 Technical Survey (Estimated Time Burden 20-40 Minutes)

The survey covers two topics: (i) technical detail relating to your quality testing undertaken for the Quality Round and (ii) and repeat (secondary) testing including sample reissues.

Optional: Please note long answers (>300 characters) or images of gates, plots or other data can be uploaded to your data folder labelled as '[Institutional Codeword] - Technical Survey Answers'. Thank you.

1. What is your Iinstitutional code word?

[ ]

Section 1 (a): Immunostaining & Flow Cytometry Technical Questions for Participants

To help us better understand your data and its significance, please answer the following technical questions regarding how you carried out the flow cytometry analysis of your Quality Round Samples.

In order to process your answers efficiently and that the data is standardized for analysis and publication, we request that your answers are in a format similar to the example answers given.

2. Cell Count

Did you perform a cell count on each of the flow cytometry samples?

◯ no, cells were not counted prior to analysis

◯ yes, cells were counted before staining and washing

◯ yes, cells were counted after washing and staining

If you answered yes, please provide detail in the comment box below, otherwise leave it empty

[ ]

3. Antibodies

Have you checked specificity using positive/negative controls or with an isotype control antibody?

(Isotype controls are a type of negative control designed to measure the level of non-specific background signal caused by primary antibodies, based upon the tissue type of the sample. Usually, the background signal is the result of immunoglobulins binding non-specifically to Fc receptors present on the cell surface.)

- ◯ no, neither a positive/negative control or isotype control was included
- ◯ yes, a positive/negative control was included
- ◯ yes, an isotype control antibody was used

If you answered yes, please give details in the comment box below.

Sample answer:

Unstained controls were used as controls.

TRA-1-60 (negative control: unstained cells)

OCT3/4 (negative control: unstained cells)

SSEA4 (negative control: unstained cells)

4. Have you (or your tester) previously used the antibodies used in the Quality Round for evaluation of PSCs?

- ◯ no, I have not used these antibodies before
- ◯ yes, I have used some of the antibodies before (please specific which)
- ◯ yes, I have used all of the antibodies before

If you answered yes, please let us know for which antibodies.

Sample answer:

TRA-1-60: YES

OCT3/4: YES

SSEA4: YES

5. Immunostaining protocol

Primary and secondary antibodies are two groups of antibodies that are classified based on whether they bind to antigens or proteins directly or target another (primary) antibody that, in turn, is bound to an antigen or protein.

Please briefly describe your staining protocol, including permeabilization method for intracellular antigens, and whether single (conjugated) vs. primary/secondary antibody was used.

Sample answer:

For cell surface antigens (SSEA-3,SSEA-4, TRA-1-60):

Cells fixed with Cytofix™ Fixation Buffer (BD 554655) were stained with the single labeled antibody for 30 min in flow cytometry buffer (2% FBS in PBS).

Intracellular antigens (OCT4, NANOG):

Cells fixed with Cytofix™ Fixation Buffer (BD 554655) were permeabilized with Perm/Wash™ Buffer (BD 554723) for 15 min and stained with the single labeled antibody for 30 min in flow cytometry buffer (2% FBS in PBS).

6. Flow cytometry results analysis

Please describe how your gating procedure, including the information about the negative control; upload a representative image to your folder and reference it here if possible.

Sample Answer:

See Survey Sample Answer Figure 1 (www.gait.global/quality-round-primary-data-set).

After gating of main populations by FSC/SSC plotting and gating out abnormal events (like doublets), negative control (unstained sample) was used for establishing the positive signal threshold: the gate boundary was set as to exclude all events of negative control sample (figure). Then, the same gate was used for the stained sample.

Sample answer:

CELL SURFACE ANTIGEN: TRA-1-60, SAMPLE F1

UNSTAINED SAMPLE
TRA-1-60-STAINED SAMPLE
/INTRACELLULAR ANTIGEN: OCT3/4, SAMPLE F1

UNSTAINED SAMPLE
OCT3/4-STAINED SAMPLE

7. Did you perform any mathematical correction on the data (e.g. compansation for signal overlap between the channels of the emission spectra calculations or equalization of some sort?

- ○ NO, no such mathematical corrections were made
- ○ YES, mathematical corrections were made. Two flours with overlapping emission spectra had to be reconciled
- ○ Details of mathematical corecction.

Sample answer:

Cells were stained with FITC SSEA-4 and with a PE isotype control, and collected at different compensation values to correct for the FITC spillover into the PE channel.

Represenative images of uncompensated and compensated cells have been uploaded to the Participant's data folder and labelled [Institutional Codename] - Compensation Data.

8. qPCR Operator

Please indicate how the genetic analysis of samples was performed:

- ◯ All runs were performed by the same operator
- ◯ Individual runs were performed by more than one operator

9. Please indicate the level of qPCR experience of operators:

- ◯ Experienced qPCR user (> 1 year experience prior to Quality Round)
- ◯ Moderate qPCR user (3 months - 1 year experience prior to Quality Round)
- ◯ Novice qPCR user (0 - 3 months experience prior to test)
- ◯ Multiple Operator Option: In cases where multiple operators performed qPCR for the Quality Round please indicate the corresponding run number next to the options in the comment box below.

If only one operator performed the run, you can leave this comment box empty.

Sample answer:

Experienced qPCR user (1 run)
Novice qPCR user (2 runs)

Section 2: OPTIONAL SECONDARY TESTING AND/OR SAMPLE REORDER REQUESTS

10. After you have assessed your data in comparison with other Participants' data, do you wish to undertake additional (secondary) testing?

- ◯ No, I do not wish to undertake secondary testing and will not be uploading further data
- ◯ Yes, I would like to retest samples and upload further Quality Testing data

11. If you wish to undertake additional testing and do you have sufficient samples for retesting?

Please note:

If you require additional NIBSC or CIRA flow cytometry samples these will be dispatched from NIBSC (at no extra charge for the sample and shipping).

If you require additional CiRA genetic analysis samples, these can be dispatched from CiRA at no extra charge for the sample – but dry ice shipping will be at Participant's expense - CiRA will organize on your behalf and bill you for the shipping.

- ◯ No, I do not wish to undertake additional testing and do not require additional samples
- ◯ Yes, I do wish to undertake additional testing but have sufficient sample matters for the retest. I need no additional samples sent to me
- ◯ Yes, I do wish to undertake additional testing but I require additional CiRA flow cytometry samples. I do not require additional CiRA flow cytometry samples or NIBSC flow cytometry samples
- ◯ Yes, I do wish to undertake additional testing but I require additional NIBSC flow cytometry samples. I do not require additional CiRA flow cytometry samples or CiRA genetic analysis samples
- ◯ Yes, I do wish to undertake additional testing but I require additional CiRA genetic analysis samples. I do not require additional CiRA nor NIBSC flow cytometry samples
- ◯ Yes, I do wish to I do wish to undertake additional testing but I require both CiRA and NIBSC flow cytometry samples. I do not require additional CiRA genetic analysis samples
- ◯ Yes, I do wish to I do wish to undertake additional testing but I require all CiRA and NIBSC flow cytometry samples and CiRA genetic testing samples

Thank you for completing the iPSC Quality Round Technical Survey (including 'sample testing' and 'sample reorder' requests).

Methods S2. QAR 2023 participant survey

iPSC Quality Assessment Round 2023 Participant Survey

The evaluator for your centre is asked to provide concise summary responses describing how their banks´ own activity meets the best practice described in the consensus guidance document ISCBI 2015.

https://www.futuremedicine.com/doi/full/10.2217/rme.14.93

The response can describe work under way to establish a system but it should be clear whether this is in process and not complete or yet to start.

If your centre is not yet actively generating iPSCs but are collecting cell and tissue sample you should only complete the relevant questions of the survey.

There are 5 parts to the survey:
(1) Donor Information and Donor Samples
(2) Preparation of induced Pluripotent Stem Cells
(3) Quality Control
(4) Safety Testing, and
(5) Characterisation

* Required

* This form will record your name, please fill your name.

Welcome

1. Institute Codename *

Donor screening & donor samples

ISCBI Guidance Section 3 Provenance and selection of donor tissue Pages 3-5

3.1 Donor Selection, Screening & Medical Records Page 3
3.2 Allogenic Cell Transplantation Page 4
3.3 Ongoing Donor Traceability Page 4
3.4 Advantageous Capture of Biological Specimens Page 4
3.5 Donor Medical Histories Page 5
Appendix 1(b) Informed Consent & Donor Disclosures Page 31

2. Do you have an informed consent process?

- [ ] Yes
- [ ] No
- [ ] Other

3. Do you use donor health and behaviour assessments and/or testing of donors for infectious agents eg. HIV, Hepatitis?

○ Yes

○ No

○ Other

4. If you answered 'yes' to the last question, please provide details below:

5. Is there a Standard Operating Procedure (SOP) for this infectious agent testing?

○ Yes

○ No

○ Unknown

○ Not Applicable (NA)

○ Other

6. Infectious agent testing is done:

- ◯ In house
- ◯ By an external contractor
- ◯ Both
- ◯ Not Applicable (NA)
- ◯ Other

7. Do you have a set of donor samples that you wish to be considered for inclusion in the GAiT haplobank system?

- ◯ Yes
- ◯ No
- ◯ Other

8. If you answered 'yes' to the last question, please provide details of this resource / these resources below:

9. If you are able to say, please state what reprogramming method(s) and starting materials you are routinely using below:

Induced Pluripotent Stem Cells Preparation

10. Have you prepared iPSC lines that you believe to be suitable for inclusion in the GAiT haplobank system?

- [ ] Yes
- [ ] No
- [ ] Other

11. If you answered 'yes' to the last question, please provide details of the cell viability tests used and the points at which cells are tested below:

12. Have you adopted any formal national or international quality standards in preparing the iPSC lines for inclusion into the GAiT haplobank system?

◯ Yes

◯ No

◯ I have not made iPSC lines yet

◯ Other

13. If you answered 'yes' to the last question, please provide details below:

Quality Control - Cell Viability & Identification Testing

14. Do you perform a test for Cell Viability?

- [ ] Yes
- [ ] No
- [ ] Other

15. If you answered 'yes' to the last question, please provide details of the cell viability tests used and the points at which cells are tested below:

16. Is there a Standard Operating Procedure (SOP) for this cell viability test?

- ○ Yes
- ○ No
- ○ Unknown
- ○ Not Applicable (NA)
- ○ Other

17. The cell viability test used is done:

- ○ In house
- ○ By an external contractor
- ○ Both
- ○ Not Applicable (NA)
- ○ Other

18. Do you perform a test for Cell Identity (e.g. DNA fingerprinting)?

- ☐ Yes
- ☐ No
- ☐ Other

19. If you answered 'yes' to the last question, please provide details of the cell identification tests used below:

20. Is there a Standard Operating Procedure (SOP) for this cell identification test?

- ◯ Yes
- ◯ No
- ◯ Unknown
- ◯ Not Applicable (NA)
- ◯ Other

21. The cell identification test used is done:

- ◯ In house
- ◯ By an external contractor
- ◯ Both
- ◯ Not Applicable (NA)
- ◯ Other

Safety Testing - Sterility testing of banked iPSC lines for viruses, bacteria & fungi

22. Do you perform a virus detection test on the banked iPSC lines?

- ☐ Yes
- ☐ No
- ☐ Other

23. If you answered 'yes' to the last question, please provide details of the viral detection tests used below:

24. Is there a Standard Operating Procedure (SOP) for this viral detection test?

- ○ Yes
- ○ No
- ○ Unknown
- ○ Not Applicable (NA)
- ○ Other

25. The viral detection test? used is done:

- ○ In house
- ○ By an external contractor
- ○ Both
- ○ Not Applicable (NA)
- ○ Other

26. Do you perform a a sterility test to detect bacteria, fungi and/or mycoplasma?

- [ ] Yes
- [ ] No
- [ ] Other

27. If you answered 'yes' to the last question, please provide details of the bacteria/fungi detection tests used below:

28. Is there a Standard Operating Procedure (SOP) for this bacteria/fungi detection test?

- ○ Yes
- ○ No
- ○ Unknown
- ○ Not Applicable (NA)
- ○ Other

29. The bacteria/fungi detection test used is done:

- ○ In house
- ○ By an external contractor
- ○ Both
- ○ Not Applicable (NA)
- ○ Other

Safety Testing - Tumorigenicity and Other Safety Tests

30. Do you perform a test for tumorigenicity?

- [ ] Yes
- [ ] No
- [ ] Other

31. If you answered 'yes' to the last question, please provide details of the tumorigenicity test used below:

32. Is there a Standard Operating Procedure (SOP) for this tumorigenicity test?

- ○ Yes
- ○ No
- ○ Unknown
- ○ Not Applicable (NA)
- ○ Other

33. The tumorigenicity test used is done:

- ○ In house
- ○ By an external contractor
- ○ Both
- ○ Not Applicable (NA)
- ○ Other

34. Do you use any tests to demonstrate the clearance of exogenous reprogramming materials from the banked iPSC cell lines eg. vector clearance?

- ☐ Yes
- ☐ No
- ☐ Other

35. If you answered 'yes' to the last question, please provide details of the additional safety tests used below:

36. Is there a Standard Operating Procedure (SOP) for this clearance test?

- ○ Yes
- ○ No
- ○ Unknown
- ○ Not Applicable (NA)
- ○ Other

37. The clearance test used is done :

- ○ In house
- ○ By an external contractor
- ○ Both
- ○ Not Applicable (NA)
- ○ Other

38. Do you perform any other safety test not already described?

- ○ Yes
- ○ No

39. If you answered 'yes' to the last question, please provide details of the additional safety tests used below:

40. Is there a Standard Operating Procedure (SOP) for this additional safety test?

- ◯ Yes
- ◯ No
- ◯ Unknown
- ◯ Not Applicable (NA)
- ◯ Other

41. The additional safety test used is done:

- ◯ In house
- ◯ By an external contractor
- ◯ Both
- ◯ Not Applicable (NA)
- ◯ Other

Characterisation - Genotyping & Pluripotency

42. Do you perform a genotyping test?

- ☐ Yes
- ☐ No
- ☐ Other

43. If you answered 'yes' to the last question, please provide details of the genotyping test used below:

44. Is there a Standard Operating Procedure (SOP) for this genotyping test?

- ◯ Yes
- ◯ No
- ◯ Unknown
- ◯ Not Applicable (NA)
- ◯ Other

45. The genotyping test used is done:

- ◯ In house
- ◯ By an external contractor
- ◯ Both
- ◯ Not Applicable (NA)
- ◯ Other

46. Do you perform a test for pluripotency?

- ☐ Yes
- ☐ No
- ☐ Other

47. If you answered 'yes' to the last question, please provide details of the pluripotency test used below:

48. Is there a Standard Operating Procedure (SOP) for this pluripotency test?

- ◯ Yes
- ◯ No
- ◯ Unknown
- ◯ Not Applicable (NA)
- ◯ Other

49. The pluripotency test used is done :

- ◯ In house
- ◯ By an external contractor
- ◯ Both
- ◯ Not Applicable (NA)
- ◯ Other

Characterisation - Phenotyping

50. Do you perform a phenotyping test?

- ☐ Yes
- ☐ No
- ☐ Other

51. If you answered 'yes' to the last question, please provide details of the phenotyping test used below:

52. Is there a Standard Operating Procedure (SOP) for this phenotyping test?

- ◯ Yes
- ◯ No
- ◯ Unknown
- ◯ Not Applicable (NA)
- ◯ Other

53. The phenotyping test used is done:

- ◯ In house
- ◯ By an external contractor
- ◯ Both
- ◯ Not Applicable (NA)
- ◯ Other

Characterisation - Cell stability/Epigenetics & Other Characterisation Test

54. Do you perform a cell stability/epigenetic characterisation test?

- ☐ Yes
- ☐ No
- ☐ Other

55. If you answered 'yes' to the last question, please provide details of the cell stability/epigenetic characterisation test used below:

56. Is there a Standard Operating Procedure (SOP) for this cell stability/epigenetic characterisation test?

- ◯ Yes
- ◯ No
- ◯ Unknown
- ◯ Not Applicable (NA)
- ◯ Other

57. The cell stability/epigenetic characterisation test used is done:

- ◯ In house
- ◯ By an external contractor
- ◯ Both
- ◯ Not Applicable (NA)
- ◯ Other

58. Do you perform any other characterisation test not already described?

- ☐ Yes
- ☐ No
- ☐ Other

59. If you answered 'yes' to the last question, please provide details of the additional characterisation tests used below:

60. Is there a Standard Operating Procedure (SOP) for this additional characterisation test?

- ◯ Yes
- ◯ No
- ◯ Unknown
- ◯ Not Applicable (NA)
- ◯ Other

61. The additional characterisation test used is done:

- ◯ In house
- ◯ By an external contractor
- ◯ Both
- ◯ Not Applicable (NA)
- ◯ Other

Technical Survey

* Required

* This form will record your name, please fill your name.

1. What is your Institute Codename? *

Quality Test 1

2. Did you perform a cell count on each of the flow cytometry samples?

- ○ no, cells were not counted prior to analysis
- ○ yes, cells were counted **before** staining and washing
- ○ yes, cells were counted **after** staining and washing
- ○ Other

3. If you answered yes to Question 2, please provide detail in the comment box below e.g method of counting/equipment type, otherwise leave it empty

4. Were there enough cells for you to run your procedure as you would routinely do?

- ○ Yes
- ○ No
- ○ Other

5. How many technical replicates did you analyse per run?

6. How many runs did you complete?

- ○ 2 complete runs
- ○ 1 complete run only
- ○ 1 complete run as first run failed.
- ○ Other

7. What type of controls did you use?

- [ ] None
- [ ] Positive Controls
- [ ] Negative Controls - e.g unstained Cells
- [ ] Isotype Controls
- [ ] FMOs
- [ ] Other

8. If you answered yes, please give details in the comment box below.
Sample answer:
Unstained controls were used as controls.
TRA-1-60 (negative control: unstained cells)
OCT3/4 (negative control: unstained cells)
SSEA-4 (negative control: unstained cells)

9. Please briefly describe your staining protocol.

Optional: Please note long answers or supplemental data related to this survey can be uploaded to your data folder labelled as 'Codename_TechnicalSurvey9-1' (additional files can be numbered -2, -3 and so on)..
Thank you.

Sample answer:
- For cell surface antigens (SSEA-3, SSEA-4, TRA-1-60):
- Cells fixed with Cytofix™ Fixation Buffer (BD 554655) were stained with the single labelled antibody for 30 min in flow cytometry buffer (2% FBS in PBS).
- Intracellular antigens (OCT3/4, NANOG):
- Cells fixed with Cytofix™ Fixation Buffer (BD 554655) were permeabilized with Perm/Wash™ Buffer (BD 554723) for 15 min and stained with the single labelled antibody for 30 min in flow cytometry buffer (2% FBS in PBS).

10. Please describe how your gating procedure, including the information about the negative control; upload a representative image to your folder and reference it here.

Name file: Codename_TechnicalSurveyQ10-1 (additional files can be numbered -2, -3 and so on).

Sample Answer:

See file Codename_TechnicalSurveyQ10-1

Sample answer:
1. All cells by FSC/SSC
2. Doublet exclusion
3. Negative control e.g. unstained sample to set gates

Please note supplemental images of gates, plots or other data related to this survey can be uploaded to your data folder labelled as 'Codename_TechnicalSurveyQ10-1' (additional files can be numbered -2, -3 and so on).

11. Did you perform any mathematical correction on the data (e.g. compensation for signal overlap between the channels of the emission spectra calculations or equalization of some sort?

Sample answer:
Cells were stained with FITC SSEA-4 and with a PE isotype control, and collected at different compensation values to correct for the FITC spill over into the PE channel.

Representative images of uncompensated and compensated cells have been uploaded to the Participant's data folder and labelled 'Codename_TechnicalSurveyQ11-1' (additional files can be numbered -2, -3 and so on).

- ○ NO, no such mathematical corrections were made
- ○ YES, mathematical corrections were made. Two fluorophores with overlapping emission spectra had to be reconciled
- ○ Other

12. If you answered YES to Question 11 please add details of mathematical correction.

Quality Test 2

13. How many assay runs did you complete?

- ◯ 2 complete runs
- ◯ 1 complete run only
- ◯ 1 complete run as first run failed.
- ◯ Other

Quality Round 2019

International External Quality Assessment Scheme (IEQAS) for human iPSC lines

Quality Assessment Round Instructions

Suppliers Instructions

Directions for iPSC Biomarker Expression Analysis by Flow-cytometry

UK Stem Cell Bank

Information for users for hPSC standards for flow cytometry.

Each vial contains ~ 1million PFA fixed hPSC cells. Each vial has 1 million cells, so participants can run up to 3-5 technical replicates as required.

They should be kept in the fridge at 4°C until ready to use.

The cells are fixed and stored in PBS without Ca++/Mg++; if nuclear staining is required a permeabilization step is necessary first.

If not, then the cells are ready to use.

Please used your local staining procedure. The standard can be used with any flow cytometer. Use your local gating strategies.

Please send back the data as % positive for each antibody tested, and the raw data as an FCS file if possible.

Images of representative plots are requested for upload to help Organizers with data amalgamation and interpretation.

STEMCELL™ TECHNOLOGIES

Supplied Instructions for Genomic Stability Analysis using StemCell Tech hPSC Genetic Analysis Kit

The kit supplied product and technical bulletin are included here for Participants convenience by STEMCELL Technologies.

qPCR analysis kit for detecting the majority of karyotypic abnormalities reported in human ES and iPS cells
Catalog #07550 60 Reactions

Product Description
hPSC Genetic Analysis Kit contains nine primer-probe mixes to detect the majority of recurrent karyotypic abnormalities reported in human embryonic stem (ES) cells and induced pluripotent stem (iPS) cells. This qPCR-based kit enables the genetic screening of multiple human ES and iPS cell lines in a rapid and cost-effective manner. It uses double-quenched probes with a 5-carboxyfluorescein (5-FAM) dye to give superior performance over other single-quenched probes.
hPSC Genetic Analysis Kit contains a Genomic DNA Control sample that has been validated as a diploid control for the regions analysed using this kit, as well as a separate ROX Reference Dye. The kit contains sufficient material to analyse 20 individual samples in triplicate (60 reactions).

Product Information
All components listed below are stable until expiry date (EXP) on label.

COMPONENT NAME	COMPONENT #	SIZE	STORAGE
qPCR Master Mix (2X)	07551	3 mL	Store at -20°C.
ROX Reference Dye	07552	0.2 mL	Store at -20°C.
Chr 1q Genetic Assay	07553	60 Rxn	Store at -20°C.
Chr 4p Genetic Assay	07554	60 Rxn	Store at -20°C.
Chr 8q Genetic Assay	07555	60 Rxn	Store at -20°C.
Chr 10p Genetic Assay	07556	60 Rxn	Store at -20°C.
Chr 12p Genetic Assay	07557	60 Rxn	Store at -20°C.
Chr 17q Genetic Assay	07558	60 Rxn	Store at -20°C.
Chr 18q Genetic Assay	07559	60 Rxn	Store at -20°C.
Chr 20q Genetic Assay	07560	60 Rxn	Store at -20°C.
Chr Xp Genetic Assay	07561	60 Rxn	Store at -20°C.
Genomic DNA Control	07562	15 µL	Store at -20°C.
TE Buffer	07563	1 mL	Store at -20°C.

Materials Required but Not Included

PRODUCT NAME	CATALOG #
Genomic DNA extraction kit	e.g. QIAGEN 69504
Costar® Microcentrifuge Tubes, 0.65 mL	38037
384-well qPCR plate	e.g. Sigma Z374911
Nuclease-free water	e.g. Sigma W4502
Optical adhesive film	e.g. Sigma Z707465

Preparation of Reagents and Materials

A. Harvesting Genomic DNA

Harvest genomic DNA from the cell line to be analysed using an appropriate genomic DNA extraction kit. If not used immediately, store harvested DNA at -20°C.

B. Master Mix + Dye

1. Thaw qPCR Master Mix (2X) and ROX Reference Dye on ice. Protect from light.
2. Add ROX Reference Dye to qPCR Master Mix (2X) according to Tables 1 and 2. For instruments not listed, refer to the manufacturer's instructions.

PCR SYSTEM	REFERENCE DYE CONCENTRATION LEVEL		
	HIGH	LOW	NONE
**Applied Biosystems**<br>• 7900HT Fast and 7300 Real-Time PCR Systems<br>• StepOne™ and StepOnePlus™ Real-Time PCR Systems	X		
**Applied Biosystems**<br>• ViiA™ 7 and 7500 Real-Time PCR Systems<br>• QuantStudio™ Flex		X	
**Agilent Technologies**<br>• Mx3005P and Mx4000P		X	
**Bio-Rad**<br>• CFX, iQ™, and DNA Engine Opticon® Real Time PCR Systems			X
**Roche**<br>• LightCycler® Real-Time PCR System			X

Table 1. Recommended Reference Dye Concentration Levels for PCR Systems

VOLUME OF MASTER MIX	VOLUME OF ROX REFERENCE DYE	
	High Reference Dye System (see Table 1)	Low Reference Dye System (see Table 1)
3 mL	120 µL	12 µL

Table 2. Volume of ROX Reference Dye to Add to Master Mix

3. Pulse vortex Master Mix + Dye on high for 3 - 5 seconds. Place on ice and protect from light.

NOTE: If not used immediately, aliquot and store at -20°C. Do not exceed the expiry date on the label. After thawing the aliquots, use immediately. Do not re-freeze.

hPSC Genetic Analysis Kit

C. Genomic DNA Control and Samples

1. Thaw Genomic DNA Control and samples on ice.

2. Measure the concentration of the genomic DNA samples to be analysed using a NanoDrop™ spectrophotometer or other method.
NOTE: At least two samples (or one sample plus the control) are required to determine copy number.
3. In a 0.65 mL microcentrifuge tube, add 1.5 µL of Genomic DNA Control to 56.5 µL of nuclease-free water (final concentration 5 ng/µL).
Vortex on high for 3 - 5 seconds. Place on ice.
4. Add 290 ng of genomic DNA sample to separate 0.65 mL microcentrifuge tubes and adjust the volume to 58 µL with nuclease-free water (final concentration 5 ng/µL). Place tubes on ice.
NOTE: If the concentration of genomic DNA samples is > 290 ng/ µL, it is recommended to dilute the sample using nuclease-free water so that an appropriate volume of genomic DNA is added (> 1 µL).
5. Vortex Master Mix + Dye (prepared in section B) on high for 5 seconds. Add 145 µL to each genomic DNA sample and the control (prepared in steps 3 - 4). Pipette up and down 2 - 3 times to mix. Place the tubes on ice and protect from light.
NOTE: Master Mix + Dye is viscous; pipette with care to avoid loss of material in the pipette tip.
D. Primer-Probe Mixes
1. Prepare the primer-probe stock solutions as follows:
a. Centrifuge the primer-probe sequences (e.g. Chr 4p Genetic Assay) at 750 x g for 10 seconds to ensure the contents are at the bottom of the tube.
b. Add 33 µL of TE Buffer to each tube. Pipette the solution up and down the sides of the tube to ensure complete resuspension.
NOTE: Use a separate pipette tip between Genetic Assays to avoid cross-contamination.
c. Centrifuge tubes at 750 x g for 10 seconds.
NOTE: If not used immediately, aliquot and store at -20°C. Do not exceed the expiry date on the label. After thawing the aliquots, use immediately. Do not re-freeze.
2. Calculate the number of reactions required for each primer-probe using the equation below:
Number of reactions per primer-probe = (Number of genomic DNA samples [including control] to be analysed x 3) + n
Where:
• For 2 - 5 samples, n = 2 OR
• For 6 - 10 samples, n = 3 OR
• For > 10 samples, n = 10% of number of samples
3. Prepare the primer-probe mixes by combining each primer-probe stock solution (prepared in step 1) with nuclease-free water in individual 0.65 mL microcentrifuge tubes. Refer to Table 3 for example volumes or calculate the volumes required as follows:
Volume of nuclease-free water = Number of reactions per primer-probe x 2.5 µL
Volume of primer-probe stock solution = Number of reactions per primer-probe x 0.5 µL
Vortex each tube on high for 5 seconds.

NUMBER OF SAMPLES	2	3	4	5	6	7	8	9	10
NUMBER OF REACTIONS	8	11	14	17	21	24	27	30	33
VOLUME OF NUCLEASE-FREE WATER (µL)	20	27.5	35	42.5	52.5	60	67.5	75	82.5
VOLUME OF PRIMER-PROBE STOCK SOLUTION (µL)	4	5.5	7	8.5	10.5	12	13.5	15	16.5
**TOTAL VOLUME (µL)**	**24**	**33**	**42**	**51**	**63**	**72**	**81**	**90**	**99**

Table 3. Example Volumes for Preparing Primer-Probe Mixes

Directions for Use
Please read the entire protocol before proceeding.
A. qPCR
1. Using a felt pen, mark a 3 x 9-well boundary for each sample on a 384-well PCR plate according to Figure 1 or Figure 2, depending on the number of samples. Tape the top edge of the plate to a microcentrifuge tube rack (or similar) to create an ~30º angle.

Figure 1. PCR Plate Array for up to 8 Samples

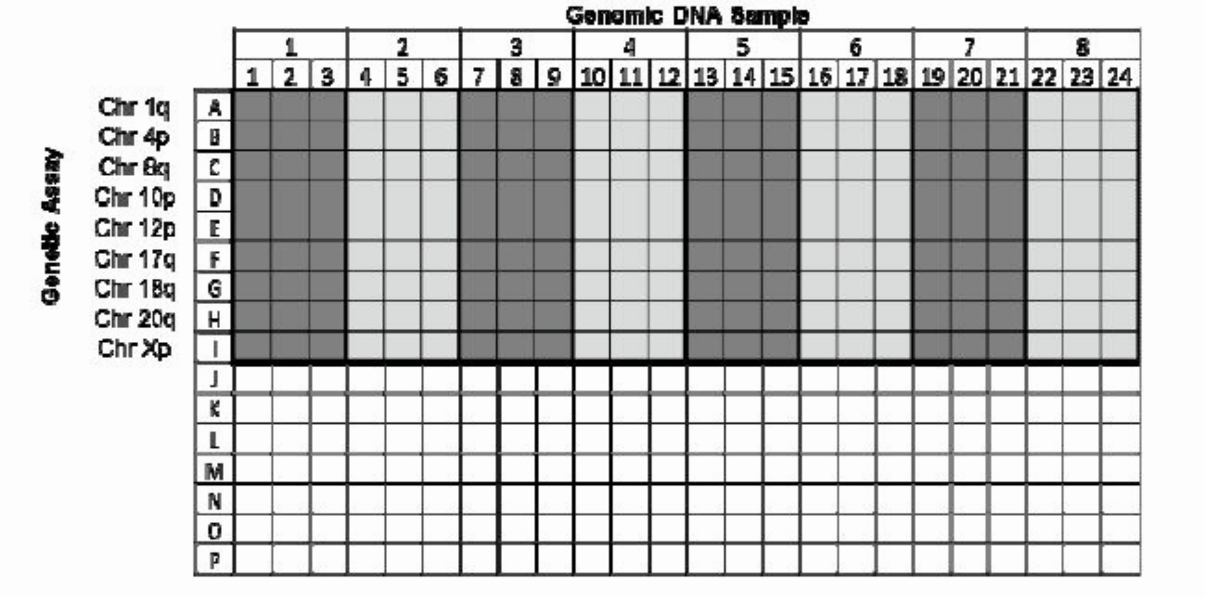

Figure 2. PCR Plate Array for 10 Samples

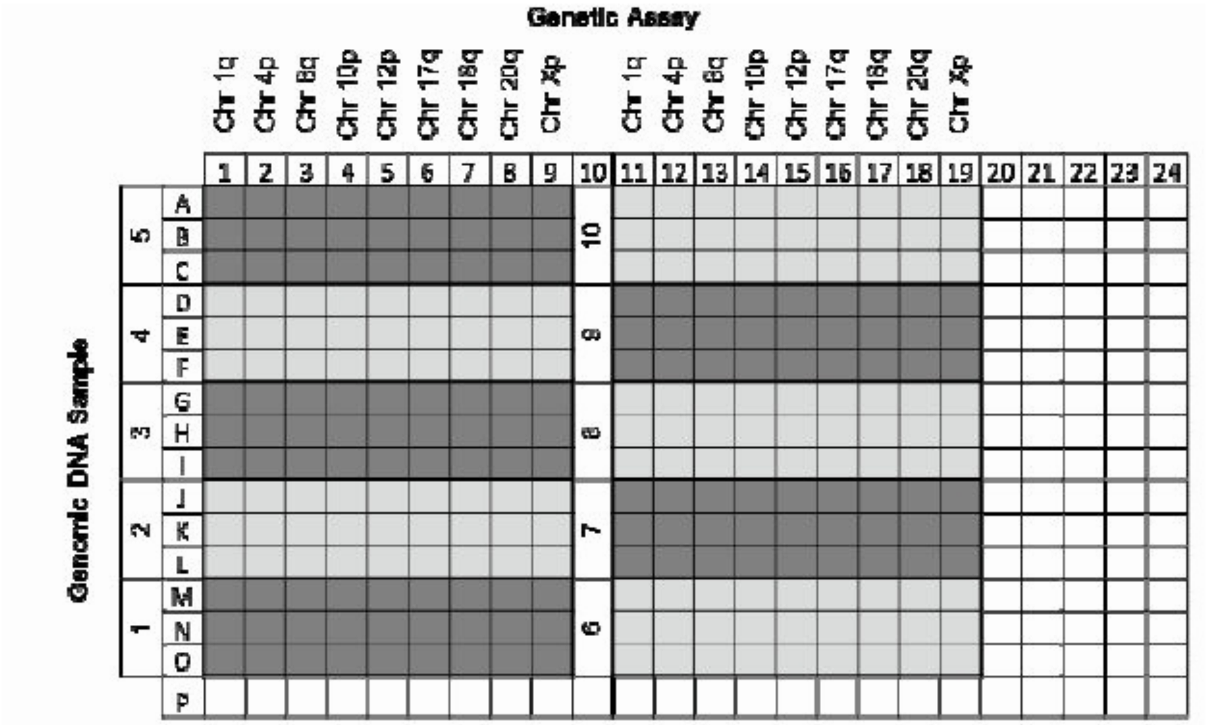

2. For the control and each genomic DNA sample prepared in section C (containing Master Mix + Dye), vortex on high for 5 seconds then centrifuge briefly to collect material at the bottom of the tube. Add 7 µL to the bottom edge of each well of the prepared plate, keeping within the marked 3 x 9-well boundary. Repeat for each sample until all samples have been loaded.
3. Working with one primer-probe mix at a time, vortex on high for 5 seconds, then centrifuge briefly to collect material at the bottom of the tube. Add 3 µL to the appropriate row of the plate, at the top edge of the well. Repeat until all primer-probe mixes have been loaded.
4. Cover the plate using optical adhesive film; use a scraper or roller to ensure that all edges and wells are sealed. Centrifuge the plate at 1000 x g for 1 - 2 minutes in a swinging bucket rotor fitted with plate holders to collect material at the bottom of the well.
5. Using a quantitative PCR system run the plate using the cycling conditions indicated in Table 4.

Table 4. PCR Cycling Conditions

STAGE	CYCLES	TEMPERATURE (°C)	FAST CYCLING TIME (min:sec)	STANDARD CYCLING TIME (min:sec)
Polymerase activation	1	95.0	03:00	03:00
Amplification:	40			
Denature		95.0	0:05	0:15
Anneal/extend		60.0	0:30	1:00

B. ANALYSIS OF RESULTS

Results obtained using hPSC Genetic Analysis Kit can be analysed using the application available at www.stemcell.com/geneticanalysisapp.

NOTE: Organize the Ct values (also referred to as Cq values) into the format shown in Figure 3; the data can then be pasted directly into the table in the application.

Figure 3. Data Organization for Analysis

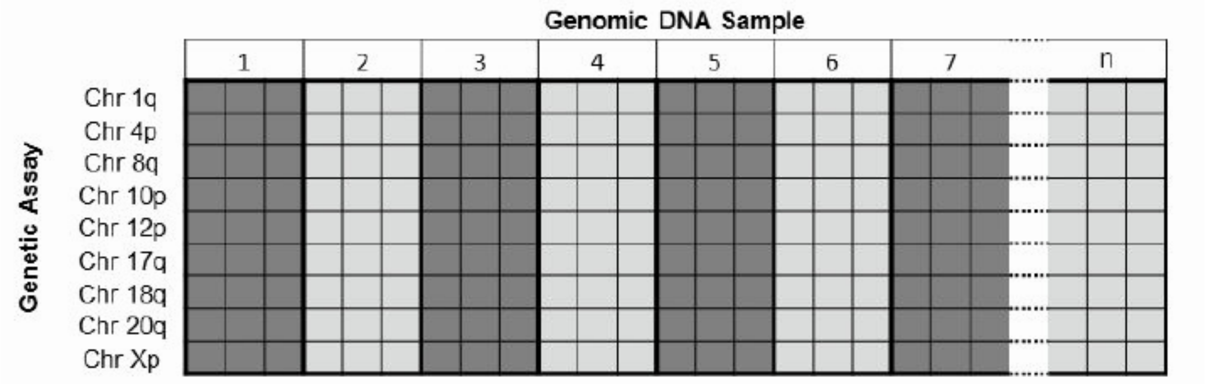

Alternatively, data can be analysed using the methods described below.

1. Calculate ΔΔCt values as follows:

Subtract the replicate Ct values of each Genetic Assay from the average Ct value of Chr 4p Genetic Assay.

NOTE: This calculation normalizes the data within each sample to account for any differences in DNA concentration between samples.

2. Calculate ΔΔCt values as follows:

Subtract the replicate Ct values of each test sample from the average Ct value of the Genomic DNA Control sample for each Genetic Assay.

NOTE: This calculation normalizes the data to a known control sample and will be the basis of determining copy number.

3. Calculate copy number as follows:

Copy number = (2-ΔΔCt) x 2

NOTE: A copy number < 1.8 or > 2.2 with a p-value*< 0.05 may indicate the presence of an abnormality within the culture.

NOTE: High variability between technical replicates will significantly impact downstream analysis. This is particularly important in any Genetic Assay within the control sample and also for the Chr 4p Genetic Assay within test samples, as this data is used for normalization.

*p-values can be calculated across all loci using a one-way ANOVA with a tukey post-hoc test. Alternatively, an unpaired t-test can be performed between the Chr 4p Genetic Assay control region and the locus of interest.

TECHNICAL BULLETIN

hPSC GENETIC ANALYSIS KIT

TECHNICAL TIPS & Recommendations

This document contains tips and recommendations for optimal use of the hPSC Genetic Analysis Kit. This document should be used in conjunction with the Product Information Sheet (PIS; Document #DX22330).

Key Tips

• When using the hPSC Genetic Analysis Kit for the first time, run the Genomic DNA Control and only 1 - 2 samples to become familiar with the protocol before screening large numbers of samples.

• Vortex the mixtures thoroughly (5 seconds per sample) when indicated in the PIS.

• It is important to be precise and careful when pipetting. A digital single-channel repeating (multi-dispenser) pipette is recommended when analysing multiple samples.

General Tips

When using the kit for the first time

1. Read the PIS thoroughly before resuspending the Genetic Assays or preparing the qPCR Master Mix + Dye.

2. The first run should be carried out on the Genomic DNA Control plus 1 - 2 samples at most. Once you are familiar with the technique and have low replicate variability, the sample number can be increased.

3. Plan your plate layout in advance and have it in front of you for reference when pipetting the reactions.

Vortex, Vortex, Vortex!

1. When working with DNA there is often a hesitation about vortexing, since it may lead to shearing. Although we do not recommend vortexing the stock genomic DNA samples, it is important to thoroughly vortex the DNA and Master Mix + Dye solution (prepared in step C5) for 5 seconds. This will help to reduce the variability between technical replicates.

2. Likewise, the Genetic Assay and water solution prepared in step D3 should be sufficiently mixed by vortexing (5 seconds per sample). This helps to reduce the variability between technical replicates.

Genomic DNA Sample Preparation

1. The amount of genomic DNA per reaction can be increased; in some cases, this can improve reproducibility between replicates; e.g. instead of using 290 ng in 58 µL (step C.4), use 580 ng or 870 ng in 58 µL.

2. Mix the genomic DNA and Master Mix + Dye, prepared by the end of C5, by vortexing thoroughly (5 seconds per sample) immediately before pipetting into the plate.

3. Determine the concentration and quality of genomic DNA samples using an appropriate method. Genomic DNA samples should have absorbance ratios in the range of A260/280 ~1.8- 2.0 and A260/230 ~1.9 - 2.2.

4. If the concentration of the sample DNA is low (< 5 ng/µL), it is beneficial to concentrate using ethanol precipitation or other suitable method prior to analysis with the hPSC Genetic Analysis Kit. Running very low concentrations of DNA may introduce high variability between replicates and lead to difficult-to-interpret results.

Genetic Assays

1. The lyophilized primer-probe pellet may have become dislodged during shipping, so it is important to centrifuge the tube prior to reconstitution. If possible, try to locate the pellet and pipette the TE Resuspension Buffer directly onto the pellet for resuspension.

2. It is important to mix the Genetic Assay thoroughly before use, particularly if the suspension is going to be aliquoted. This can be done by flicking the tube followed by brief centrifugation.

3. Take care when pipetting the Genetic Assays; always use a clean pipette tip when moving between stocks or diluted assays to avoid cross-contamination of primer-probes.

Troubleshooting

qPCR Reaction Fails/No Amplification

All reactions fail to amplify:

• Incorrect amount or no ROX Reference Dye added to qPCR Master Mix (2X).

Note: Check that the correct amount of ROX Reference Dye was added to the qPCR Master Mix (2X) for the machine being used.

• Incorrect cycling conditions used.

Note: If you are unsure which cycling conditions are required for your machine, use the standard cycling times provided in the PIS.

Individual reactions fail to amplify:

• One or more components are missing from the reaction including genomic DNA, qPCR Master Mix (2X), ROX Reference Dye, or Genetic Assay.

Note: Take care when pipetting the reactions together; marking sample borders can help to keep track of wells that have been filled.

• Incorrect wells selected in the qPCR software.

Note: Some qPCR machines use software that require the pre-selection of wells and their contents; check that the correct wells are selected prior to performing qPCR.

High Variability Between Technical Replicates

• Inaccurate pipetting of samples/reagents.

Note: Properly calibrated repeating pipettes can be used to reduce variability.

• Insufficient mixing/vortexing of reagents.

Note: Where indicated in the PIS, mixtures should be sufficiently mixed by vortexing.

• Insufficient volume of mixture.

Note: If prepared correctly, the genomic DNA + qPCR Master

Mix solution created in step C and the Genetic Assay mixes created in step D should have sufficient excess material to allow for loss during pipetting.

• Inaccurate loading of reagents into qPCR plate.

Note: Take care when pipetting the reagents into the qPCR plate; loading variable amounts of DNA between wells will affect amplification dynamics.

Data Format Requirements

- Raw flow-cytometry data should be in FCS format and raw rtPCR data should be in the format below and should be uploaded to the GAiT.global Quality Round portal at www.gait.global/qualityrounddataportal. A GAiT username and password is required to access this section of the GAiT website.
- rtPCR should be uploaded as an Excel spreadsheet with the following headings: TE, HE, TE-HE, TC-HC, ΔCTE, & ΔCTC, & ΔΔCt.
- Cell line nomenclature in the format suggested by Kurtz et al. (2018) [1].

Quality Round Data Portal

NIBSC
Confidence in Biological Medicines

NIBSC Standards Reporting Template

- Note: Report back in % positive
- Use local antibodies/flow machine/gating strategies
- Please also send back raw data file

Cell Line 1

Antibody	Vial 1	Vial 2	Vial 3
Oct 4			
Nanog			
Sox-2			
SSEA4			
SSEA1			
Tra-160			
Antibody XXX			
Antibody XXX			
Antibody XXX			

Cell Line 2

Antibody	Vial 1	Vial 2	Vial 3
Oct 4			
Nanog			
Sox-2			
SSEA4			
SSEA1			
Tra-160			
Antibody XXX			
Antibody XXX			
Antibody XXX			

Cell Line 3

Antibody	Vial 1	Vial 2	Vial 3
Oct 4			
Nanog			
Sox-2			

SSEA4			
SSEA1			
Tra-160			
Antibody XXX			
Antibody XXX			
Antibody XXX			

Flow Cytometer Used:XXX

Antibody	Supplier	Catalogue Number
Oct 4		
Nanog		
Sox-2		
SSEA4		
SSEA1		
Tra-160		
Antibody XXX		
Antibody XXX		
Antibody XXX		

References

1. Kurtz A, Seltmann S, Bairoch A *et al*. A Standard Nomenclature for Referencing and Authentication of Pluripotent Stem Cells. *Stem Cell Reports* 10(1), 1-6 (2018).

Methods S4. QAR 2023 Quality Test 1 and 2 instructions to participants

QUALITY ASSESSMENT ROUND 2023

A Quality Assessment Scheme

for human induced Pluripotent Stem Cell lines

Quality Test 1 Instructions

The samples can be used with any flow cytometer. Use your local gating strategies, reagents and protocol.

Quality Assessment Round (QAR) Participants are required to test the QAR samples with a minimum of two markers from the standard hPSC panel (positive for OCT4, TRA-1-60, TRA-1-81, SSEA-3, SSEA-4, SOX2, NANAOG) as they normally do as part of the iPSC quality testing. A combination of at least one intracellular (e.g., OCT4, SOX2 or NANOG) and one extracellular (e.g. SSEA-4 or TRA-1-60, TRA-1-81) is required.

Ensure **all events** are recorded and save data as **FCS files**.

Analysis for additional markers, that comprise QAR Participant's routine iPSC quality testing, should also be included and shared. When nuclear staining, Participants are asked to use their own standard protocol, as they normally would. It is important to fill out the data templates thoroughly so QAR Organisers can independently analyse and interpret the results appropriately.

Please upload to your GAiT portal folder:

- The data as **% positive** for each antibody tested using the template provided
- The raw data as **FCS files**
- The **plots or histograms (graphs)** of analysed data

Sample Information and Template Completion

Samples provided for Quality Test 1 are as follows:

Set 1: Cell 1-X、Cell 1-Y、Cell 1-Z

Set 2: Cell 2-X、Cell 2-Y、Cell 2-Z

Cell samples should be stored at or below -80°C until ready to use.

Set 1 and Set 2 cells **cannot** be mixed. Please use one complete set per run.

Each vial contains up to **4.3 x 10^6 PFA fixed cells** in 0.8 mL CellBanker. As a Quality Test run requires 1 complete set (3 different cell type; X,Y and Z), participants have enough samples to complete up to 2 runs.

The sample sets provided for Quality Test 1 can be used as follows:

- Individually for two separate runs (Run 1 and Run 2 on the template)
  OR
- The second set can be used as a backup if error occurs with the first set of cells.

PLEASE ENSURE YOU CLEARLY RECORD THE LABEL OF EACH SAMPLE ANALYSED ON THE TEMPLATE (1X, 1Y, 1Z, 2X, 2Y or 2Z)

Run definition: A run is defined as an individual assay set up, either performed by different operators or by the same operator on a different occasion.

Replicate definition: A replicate is a test of the same sample within a run.

The number of replicates within each run will be determined by each Institute's protocol, all results should be added to the template and the total mean % positive events for each antibody tested should also be calculated:

For example:

Run 1 Sample 1X (% Positive events for each antibody)

Antibody Name (e.g SSEA-4 FITC)	Replicate 1 (%)	Replicate 2 (%)	Replicate 3 (%)	Mean (%)
SSEA-4 FITC	97.2	92.4	94.6	94.7

Note: Please add additional columns or rows to the template to ensure all data is captured. For example, if more replicates or antibodies are included.

Naming of Data Files for Upload to GAiT Website Portal

Data template sheets should be labelled in the following format:

[INSTITUTIONAL CODENAME]_Quality Test (QT) [Quality Test number]. Examples:

Butterfly_QT 1 would be data template Quality Test 1 from Participant codenamed 'butterfly'

Raw data files should be labelled in the following format:

[INSTITUTIONAL CODENAME] Quality Test (QT) [Quality Test number]_[sample numbers]_[epitope qualifier if necessary]_[Run number]_[Replicate number]

Examples:

Glowworm_QT1_1X_OCT4_Run1_Rep2 would be raw data from Participant codenamed 'Glowworm' for Quality Test 1 for samples 1X, stained for OCT4 tested in Run 1 as sample replicate 2.

Spider_QT1_2Y_OCT4_SSEA-4_Run2_Rep3 would be raw data from Participant codenamed 'Spider' for Quality Test 1 for samples 2Y, stained for OCT4 and SSEA-4, tested in Run 2 as sample replicate 3.

Data Analysis plots/histograms should be labelled in the following format:

[INSTITUTIONAL CODENAME] Quality Test (QT) [Quality Test number]_[sample numbers]_[epitope qualifier if necessary]_[Run number]_[Replicate number]_GRAPH

Examples:

Glowworm_QT1_1X_OCT4_Run1_Rep2_GRAPH would be the graph from Participant codenamed 'Glowworm' for Quality Test 1 for samples 1X, stained for OCT4 tested in Run 1 as sample replicate 2.

Quality Test 2 Instructions

Protocol for undifferentiated marker expression analysis by flowcytometry for the Quality Assessment Round 2023.

This protocol is based on the CiRA_F validated quality test for undifferentiated marker expression of iPSCs and has been modified with reference to the methods of each Participant from the Quality Assessment Round kick off meeting and experts' comments. To reduce human error, the protocol is simplified compared to what is routinely performed. Although daily tests in CiRA_F are performed with live cell/fixed cells, to enable all participants to use the same cell samples, only fixed cells will be provided from CiRA_F. In this Quality Assessment Round, we will verify whether equivalent analysis can be carried out by 23 institutions.

Each vial contains up to **4.3 x 10^6 PFA fixed cells** in 0.8 mL CellBanker. As a single test needs 1 set (3 different cell type; X, Y and Z), participants have enough samples to complete up to 2 runs.

Equipment required:

PBS
FACS buffer (if you wish to use)
1.5 mL tube
15 mL tube
50 mL tube
Flow cytometry tubes
Nylon mesh strainer/ filter (≧35 μm)
Adjustable pipette
Pipette tips
Liquid nitrogen storage or deep freezer
Water bath
Centrifuge
Flow cytometer

Kit components

Antibodies:

Antibody-A (Ab-A): 2 tubes 35 μL each
Antibody-B (Ab-B): 2 tubes 10 μL each
Antibody-C (Ab-C): 2 tubes 70 μL each
Antibody-D (Ab-D): 2 tubes 35 μL each
Storage temperature: 4°C
All antibodies are conjugated with Alexa Fluor® 488 or FITC

Cryopreserved fixed cell:

Set 3: Cell 3-X、Cell 3-Y、Cell 3-Z

Set 4: Cell 4-X、Cell 4-Y、Cell 4-Z

Cell samples should be stored at or below -80°C until ready to use.

Set 3 and Set 4 cells **cannot** be mixed. Please use one complete set per run.

Buffer solution:

Buffer-K (10x): 4 tubes 9 mL each

Storage temperature: 4°C

Procedure

1. Tube and buffer preparation

1. Dilute 4 mL of Buffer-K (10x) with 36 mL of PBS in a 50 mL tube
2. Label 3 of 15 mL tubes as 3X, 3Y and 3Z for cell suspension (4X, 4Y and 4Z for set 4)
3. Label 5 of 1.5 mL tubes for Ab-A, Ab-B, Ab-C, Ab-D and Ab-U for staining buffer
4. Label 15 of 1.5 mL tubes for cell staining as on the list below

Tube labeling list:

<table>
<tr><th>Tube</th><th>Label</th></tr>
<tr><td rowspan="3">Cell Suspension 15ml tubes (3)</td><td>3X</td></tr>
<tr><td>3Y</td></tr>
<tr><td>3Z</td></tr>
<tr><td rowspan="5">Antibody Staining Buffer 1.5ml tubes (5)</td><td>Ab-A</td></tr>
<tr><td>Ab-B</td></tr>
<tr><td>Ab-C</td></tr>
<tr><td>Ab-D</td></tr>
<tr><td>Ab-U</td></tr>
<tr><td rowspan="15">Cell Staining 1.5ml tubes (15)</td><td>XA</td></tr>
<tr><td>XB</td></tr>
<tr><td>XC</td></tr>
<tr><td>XD</td></tr>
<tr><td>XU</td></tr>
<tr><td>YA</td></tr>
<tr><td>YB</td></tr>
<tr><td>YC</td></tr>
<tr><td>YD</td></tr>
<tr><td>YU</td></tr>
<tr><td>ZA</td></tr>
<tr><td>ZB</td></tr>
<tr><td>ZC</td></tr>
<tr><td>ZD</td></tr>
<tr><td>ZU</td></tr>
</table>

2. Staining buffer preparation

1. Add the following volumes of antibody and buffer-K (X1) to the labelled Staining buffer tubes
2. Mix well by tapping

Staining buffer tube label	Ab-A	Ab-B	Ab-C	Ab-D	UAb
Buffer-K (X1) (μL)	90	100	90	90	100
Antibody: volume (μL)	Ab-A: 9	Ab-B: 2	Ab-C: 18	Ab-D: 9	-
Total (μL)	99	102	108	99	100

Keep the diluted antibodies in a dark place at 4°C until ready to use.

3. Preparation of cell samples

1. Add 5 mL of Buffer-K (x1) into the 3 cell suspension tubes (15 mL) each
2. Thaw **one set** of cells X, Y and Z vials (one vial each) in a 37 °C water bath immediately after taking the vials from the storage.
3. Transfer the cells into the cell suspension tube.
4. Split the cell suspension into 5 cell staining tubes (1 mL / tube)

For Example:

Cell 3-X:		Cell 3-Y:		Cell 3-Z:	
	XA		YA		YA
	XB		YB		YB
	XC		YC		YC
	XD		YD		YD
	XU		YU		YU

(Total 15 tubes)

4. Staining procedure

1. Centrifuge at 200 x g for 5 min and remove the supernatant
2. Add the following:

Antibody	Tubes
30 μL of Ab-A	XA, YA, ZA
30 μL of Ab-B	XB, YB, ZB
30 μL of Ab-C	XC, YC, ZC
30 μL of Ab-D	XD, YD, ZD
30 μL of Ab-U	XU, YU, ZU

3. Incubate at RT for 15 min in a dark place.
4. Mix cell suspension by tapping and stand at for another 15 min in a dark place (30 min incubation in total)
5. Add 1mL of Buffer-K (x1) to each tube
6. Centrifuge at 200 x g for 5 min and remove the supernatant
7. Resuspend stained cells to 250 μL of PBS or FACS Buffer
8. Filter cell suspensions through a nylon mesh strainer to a flow cytometry tube
9. Store at 4°C in a dark place until the start of flow cytometric measurements

5. Acquisition, Analysis

Acquire the respective stained cells on the validated flow cytometer (at least 10,000 events at P3)

1. **P1 gating**: Create a forward scatter (FSC)-Area vs side scatter (SSC)-Area density plot and gate on the population of interest
2. **P2 gating**: Create an FSC-Height vs HSC-Width density plot, show only the P1 gated population, and gate on the single-cell population
3. **P3 gating**: Create an SSC-Height vs SSC-Width density plot, show only the P2 gated population, and gate on the single cell population
4. Create a FITC-A vs Counts histogram and show only the P3 gated cell population. Draw a gate to set the range of fluorescence values that will define FITC positive Ab stained cells

Unstained Control	Samples			
XU	XA	XB	XC	XD
YU	YA	YB	YC	YD
ZU	ZA	ZB	ZC	ZD

UAb stained cells show the background level of fluorescence as a negative control. Overlay of the UAb stained population onto the Ab stained population allows easy identification of the FITC positive cells

5. Obtain the mean fluorescence intensity (MFI) of FITC and the percentage of marker expression in the P3 gated population.
6. Save raw data and analysed data as FSC files

Please upload to your GAiT portal folder:

- The data as **% positive** for each antibody tested using the template provided
- The raw data as **FCS files**
- The **plots or histograms (graphs)** of analysed data

Template completion

The sample sets provided for Quality Test 2 can be used as follows:

- Individually for two separate assay runs (Run 1 and Run 2 on the template)

OR

- The second set can be used as a backup if error occurs with the first set of cells

PLEASE ENSURE YOU CLEARLY RECORD THE LABEL OF EACH SAMPLE ANALYSED ON THE TEMPLATE (3X, 3Y, 3Z, 4X, 4Y or 4Z).

Run definition: A run is defined as an individual assay set up, either performed by different operators or by the same operator on a different occasion.

Naming of Data Files for Upload to GAiT Website Portal

Data template sheets should be labelled in the following format:

[INSTITUTIONAL CODENAME]_Quality Test (QT) [Quality Test number]. Examples:

Butterfly_QT 2 would be data template Quality Test 2 from Participant codenamed 'butterfly'

Raw data files should be labelled in the following format:

[INSTITUTIONAL CODENAME] Quality Test (QT) [Quality Test number]_[sample numbers]_[Antibody]_[Run number]

Examples:

Glowworm_QT2_3X_A_Run1 would be raw data from Participant codenamed 'Glowworm' for Quality Test 2 for samples 3X, stained for Ab-A tested in Run 1.

Spider_QT2_4Y_B_Run2 would be raw data from Participant codenamed 'Spider' for Quality Test 2 for samples 4Y, stained for Ab-B, tested in Run 2.

Data Analysis plots/histograms should be labelled in the following format:

[INSTITUTIONAL CODENAME] Quality Test (QT) [Quality Test number]_[sample numbers]_[Antibody]_[Run number]_GRAPH

Examples:

Glowworm_QT2_3X_A_Run1_GRAPH would be graph from Participant codenamed 'Glowworm' for Quality Test 2 for samples 3X, stained for Ab-A tested in Run 1.
