# Supplementary material for: Toward standardized iPSC testing: Insights from a multi-year international Quality Assessment Round
Source: Stem Cell Reports. 2026 Mar 19;21(4):102857. doi: 10.1016/j.stemcr.2026.102857 (PMC13083784; doi:10.1016/j.stemcr.2026.102857)
Supplement: Document S2. Article plus supplemental information [file mmc2.pdf]

STEM CELL REPORTS
Resource
OPEN ACCESS

Toward standardized iPSC testing: Insights from a multi-year international Quality Assessment Round

Alice Hägg,[1] Rachel Wood,[2] Ayako L. Mochizuki,[3] Keren Abberton,[4] Elsa Abranches,[5] Belén Alvarez-Palomo,[6] Ricardo Baptista,[7] Raiana Andrade Quintanilha Barbosa,[8] Jacqueline Barry,[9] Adriana Bastos Carvalho,[8] Annelise Bennaceur Griscelli,[10] Antonio Carlos Campos de Carvalho,[8] Diana Chaker,[10] Hong Chang,[11] Hye Young Choi,[12] Margarita Codinach,[6] Begoña Arán Corbella,[13] Scott Cowan,[2] Sarah Jane Dickerson,[14] Ngaire Elwood,[4,15] Xueling Fan,[11] Maxime Feyeux,[16] Maddy Forrester,[17] Andrew Gaffney,[17] Solenn M. Guilbert,[16] Hye-Yeong Ha,[12] Adam J. Hirst,[17] Arwen L. Hunter,[17] Leanne G. Jamieson,[39] Robert N. Judson,[17] Yonehiro Kanemura,[18] Tais Hanae Kasai-Brunswick,[8] Jung-Hyun Kim,[12,41] Howard Kim,[19] Manisha Kintali,[19] Siddharth Krishnan,[9] Bernd Kuebler,[13] Chui Yu Lau,[36] Wilson Li,[9] Amanda Mack,[20] Michael R. MacLeod,[11] Marinna Madrid,[21] Hiroaki Mamiya,[22] Lucie Manache-Alberici,[16] Dragoș Mărginean,[9] Olivier Mentre,[11] Stefanie L. Morgan,[21,42] Joanne Mountford,[2] Humayun Munir,[19] Siemon H.S. Ng,[23,43] Haruna Ogawa,[3] Steve Oh,[24,40] Hidetaka Ohara,[25] Keiko Oono,[3] Niall Park,[2] Lygia V. Pereira,[26] Izabella Pereira da Silva Bezerra,[8] Alexandru Robert Podovei,[9] Sergio Querol,[27] Jainy Raje,[28] Angel Raya,[29] Satoko Sakamoto,[25] Raquel Sarafian,[26] Kathleen Schmit,[16] Silvia Selvitella,[13] Gurbind Singh,[30] Matthew J.K. Smart,[9] Jihwan Song,[37] Glyn Stacey,[31,32,33] Stephen Sullivan,[34] Miho Sumida,[18] Cecile Terrenoire,[19] Pei Tian,[4] Elias Uhlin,[1] José M.A. Vaquero,[13] Anna Veiga,[13] Jar Wei Vicky Wang,[17] Katherine Warre-Cornish,[35] Jamie Wood,[38,44] Atsuyo Yamamoto,[18] Gaojun Zhang,[11] Takafusa Hikichi,[3] Marc Turner,[2,*] and Anna Falk[1,45,*]

[1]Department of Experimental Medical Science (EMV), Lund Stem Cell Center, Science for Life Laboratory, Sölvegatan 17 A11, 221 84 Lund, Sweden
[2]Scottish National Blood Transfusion Service, The Jack Copland Centre, 52 Research Avenue North, Heriot-Watt Research Park, Edinburgh EH14 4BE, UK
[3]CiRA Foundation, 53 Shogoin kawahara-cho, Sakyo-ku, Kyoto 606-8397, Japan
[4]Stem Cell Medicine, Murdoch Children's Research Institute, 50 Flemington Rd, Parkville, VIC 3052, Australia
[5]UK Stem Cell Bank, Medicines and Healthcare Products Regulatory Agency (MHRA), South Mimms Campus, Blanche Lane, South Mimms, Hertfordshire EN6 3QG, UK
[6]Banc de Sang i Teixits, Barcelona, Spain
[7]SmartCella, Alfred Nobels Allé 150, 146 48 Tullinge-Stockholm, Sweden
[8]Research Center for Precision Medicine, Carlos Chagas Filho Biophysics Institute Federal University of Rio de Janeiro, Rua Carlos Chagas Filho 373, Ilha do Fundão, Rio de Janeiro, RJ 21941-902, Brazil
[9]Cell and Gene Therapy Catapult, 12th Floor Tower Wing, Guy's Hospital, Great Maze Pond, London SE1 9RT, UK
[10]University Paris-Saclay, INSERM, UMS 45 CITHERA – Center for iPS Cell Therapies, National Infrastructure INGESTEM, Paris, France
[11]Center for Commericialization of Regenerative Medicine (CCRM), #1002, 661 University Ave, Toronto, ON M5G 1M1, Canada
[12]National Center for Stem Cell and Regenerative Medicine, National Institute of Health, 202 Osongsaengmyeong2-ro, Osong-eup, Heungdeok-gu, Cheongju-si, Chungcheongbuk-do 28160, South Korea
[13]Barcelona Stem Cell Bank, Regenerative Medicine Program, Institut d'Investigació Biomèdica, IDIBELL, P-CMR[C]-Program of Translation of Regenerative Medicine in Catalonia, Catalonia, Spain
[14]FUJIFILM Cellular Dynamics Inc, Madison, WI 53711, USA
[15]Department of Paediatrics, University of Melbourne, Parkville, VIC 3052, Australia
[16]TreeFrog Therapeutics, 30 Av. Gustave Eiffel Bâtiment A, 33600 Pessac, France
[17]STEMCELL Technologies, 1618 Station St, Vancouver, BC V6A 1B6, Canada
[18]Department of Biomedical Research and Innovation, Institute for Clinical Research, NHO Osaka National Hospital, Osaka 540-0006, Japan
[19]New York Stem Cell Foundation Research Institute, 619 West 54th Street, New York, NY 10019, USA
[20]Dark Horse Consulting Group, 1255 Treat Blvd # 230, Walnut Creek, CA 94597, USA
[21]Cellino, 750 Main St, Cambridge, MA 02139, USA
[22]College of Pharmaceutical Sciences, Ritsumeikan University, 1-1-1 Noji-Higashi, Kusatsu, Shiga 525-8577, Japan
[23]Notch Therapeutics, Toronto, ON, Canada
[24]Stem Cell Group, Bioprocessing Technology Institute, A*STAR, Singapore, Singapore
[25]Sumitomo Pharma Co., Ltd., 33-94, Enoki-cho, Suita, Osaka 564-0053, Japan
[26]National Laboratory for Embryonic Stem Cells (LaNCE), Department of Genetics and Evolutionary Biology, Institute of Biosciences, University of São Paulo, São Paulo, SP 05508-090, Brazil
[27]Josep Carreras Leukaemia Foundation, Muntaner 383, 2nd, 08021 Barcelona, Spain
[28]BlueRock Therapeutics, Toronto, ON, Canada
[29]ICREA, Regenerative Medicine Program, Institut d'Investigació Biomèdica de Bellvitge (IDIBELL), P-CMR[C]-Program of Translation of Regenerative Medicine in Catalonia, Center for Networked Biomedical Research on Bioengineering, Biomaterials and Nanomedicine (CIBER-BBN), Physiological Sciences Department, University of Barcelona, Barcelona, Spain
[30]Centre for Stem Cell Research (a unit of BRIC-inStem, Bengaluru), Christian Medical College Campus, Bagayam, Vellore, Tamil Nadu 632002, India
[31]International Stem Cell Biobanking Initiative, Barley, Hertfordshire SG88HZ, UK
[32]National Stem Cell Resource Centre, Institute of Zoology, Chinese Academy of Sciences, Beijing 100190, China
[33]Beijing Institute for Stem Cell Regenerative Medicine and Chinese Academy of Sciences, Beijing 100101, China
[34]Lindville Bio, 69 Warrender Park Road, Edinburgh, UK
[35]Medicines and Healthcare products Regulatory Agency, London, UK

Published by Elsevier Inc. on behalf of International Society for Stem Cell Research.
This is an open access article under the CC BY license (http://creativecommons.org/licenses/by/4.0/).

[36]Century Therapeutics, 3624 Market Street, #5 Floor West, Philadelphia, PA 19104, USA
[37]CHA University, Rm 604, CHA Bio Complex, 335 Pangyo-ro, Bundang-gu, Seongnam-si, Gyeonggi-do 13488, Republic of Korea
[38]Formerly Medicines and Healthcare Products Regulatory Agency, London, UK
[39]BlueRock Therapeutics (2019) and Notch Therapeutics (2023/2024), Toronto, ON, Canada
[40]Present address: Vin University, Hanoi, Vietnam
[41]Present address: Ajou University, Suwon-si, Gyeonggi-do, 16499, Republic of Korea
[42]Present address: AgelessRx, 2370 E Stadium Blvd, Ann Arbor, MI 48104, USA
[43]Present address: Independent Consultant, Toronto, ON, Canada
[44]Present address: Cardiff University, Cardiff, UK
[45]Lead contact
*Correspondence: marc.turner2@nhs.scot (M.T.), anna.falk@med.lu.se (A.F.)
https://doi.org/10.1016/j.stemcr.2026.102857

SUMMARY

Despite rapid clinical translation, induced pluripotent stem cell (iPSC)-derived therapies face limited global adoption. Harmonized quality control (QC) remains absent, with even fundamental parameters evaluated inconsistently across laboratories. To address this, we conducted two international Quality Assessment Rounds (QARs): QAR 2019 (18 sites, 11 countries) and QAR 2023 (23 sites, 12 countries), evaluating flow cytometry-based assessment of the undifferentiated state and qPCR-based genomic integrity testing. QAR 2019 showed high consistency in genomic integrity testing, while uncovering substantial variability in flow cytometry, prompting QAR 2023 to introduce standardized workflows. These improvements enabled systematic, cross-site evaluation of marker performance across cell states, identifying OCT3/4, TRA-1-60, and SSEA5 as consistently robust pluripotency-associated markers. This global benchmarking effort provides the first empirical multi-site evidence for reproducible iPSC QC and marker-level reliability. Together, these findings establish a foundation for harmonized QC supporting interoperable iPSC banks, regulatory alignment, and scalable manufacturing of globally accessible regenerative therapies.

INTRODUCTION

Induced pluripotent stem cells (iPSCs) have attracted significant interest as a renewable source of cells for the scalable manufacture of advanced therapy medicinal products (ATMPs), suitable for both autologous and allogeneic applications. Pluripotent cell-based ATMPs are advancing rapidly through clinical pipelines worldwide, with an increasing number of trials and several products approaching regulatory approval (hPSCreg; Kirkeby et al., 2025). These advances highlight the substantial therapeutic potential of iPSC-based therapies, while underscoring the need for consistent, scalable manufacturing processes and high-quality starting materials.

The widespread adoption of iPSC-based therapies will depend on establishing international iPSC banks to ensure a reliable and accessible supply. Achieving this goal requires robust quality control (QC) strategies that enable standardized testing and well-defined release criteria across diverse clinical, regulatory, and geographic contexts. Despite increasing clinical and regulatory activity, no comprehensive, globally harmonized framework has yet been universally accepted for assessing iPSC identity, purity, and genomic integrity. Existing regulatory guidelines are primarily adapted from earlier biotechnological standards and remain fragmented and inadequately implemented across regions (Barry et al., 2015; Martins and Ribeiro, 2025), reflecting the early stage of development for PSC-based products. While some PSC-derived therapies have entered phase 1–3 clinical trials, none have yet achieved full market authorization (hPSCreg). Regulatory approvals from agencies such as the Food and Drug Administration and European Medicines Agency represent critical milestones, and the associated QC strategies are often regarded as *de facto* guidelines for the cell therapy field. However, these regulatory frameworks are insufficient to ensure global scalability, as they typically involve product- and jurisdiction-specific requirements that must be extensively re-validated when applied to different products, processes, or facilities. In the absence of harmonized standards, each therapy or manufacturing site is treated as a standalone case, requiring *de novo* validation that slows progress, increases costs, and limits interoperability. This lack of coordination presents a major barrier to the widespread clinical implementation of iPSC-based therapies.

Importantly, the lack of standardization in QC practices could also hinder global equity. Many low- and middle-income countries may lack the infrastructure or regulatory capacity to implement and validate complex QC workflows without additional support (World Health Organization, 2018). Without harmonized, accessible workflows, these regions risk exclusion from both the development and the benefits of iPSC-based therapies. A globally accepted QC framework would allow centralized iPSC banks to distribute high-quality material worldwide (Escribá et al., 2024), while allowing local laboratories to verify identity and safety using shared, validated protocols. In this context, assay variability is not merely a technical issue, but a structural barrier to equitable progress in regenerative medicine.

Reliable assessment of pluripotency is essential for confirming iPSC nature. Although the *in vivo* teratoma assay has traditionally been considered a gold standard, its reliance on animal models, cost, and low throughput limit its routine use. Alternative methods, such as tri-lineage differentiation and transcriptomic profiling, have been explored, yet no consensus on a single best practice has emerged (International Stem Cell Initiative, 2018). Many of these approaches rely on the ability of cells to differentiate, highlighting the relevance of functionally oriented assays for pluripotency assessment (Abranches et al., 2020).

Flow cytometry remains the most widely used technique for assessing the undifferentiated state of iPSCs. Typically, co-expression of at least one intracellular marker (e.g., OCT3/4, NANOG, SOX2) and one surface antigen (e.g., TRA-1-60 or SSEA4) is evaluated, with >70% positive expression generally expected for undifferentiated iPSCs (Andrews et al., 2015; Sullivan et al., 2018). Each pluripotency-associated marker, however, has unique context-specific strengths and limitations. Misinterpretation, such as relying on a single marker or neglecting assay context, can lead to inaccurate conclusions about the pluripotent state of the iPSC bank. Careful validation of marker combinations and assay design is, therefore, critical to ensure accurate interpretation and reliable assessments. Despite this, substantial variability persists across laboratories in the selection of marker panels, antibody clones, staining protocols, and data interpretation thresholds. Beyond protocol differences, the performance of individual markers can vary by site and sample state, underscoring the need for systematic, multi-site benchmarking to identify the most reliable markers for both undifferentiated and differentiated samples.

Genomic integrity represents another cornerstone of iPSC quality, as recurrent chromosomal abnormalities can arise during reprogramming and extended culture (Andrews et al., 2017; Halliwell et al., 2020; International Stem Cell Initiative et al., 2011; Merkle et al., 2017; Weissbein et al., 2019). Some alterations may confer a selective growth advantage (Avery et al., 2013) or mirror genomic alterations seen in cancers (Peterson and Loring, 2014; Wuputra et al., 2020), raising safety and efficacy concerns. A range of complementary assays, including Giemsa-banding karyotype (G-banding), chromosomal microarray (CMA), next-generation sequencing (NGS), fluorescent *in situ* hybridization, and polymerase chain reaction (PCR), is often used to assess genomic stability, as each offers distinct detection capabilities. G-banding remains a widely used first-line screen, enabling identification of large-scale abnormalities (>5–10 Mb), translocations (balanced and unbalanced), and high-level mosaicism (Abranches et al., 2020). CMA and NGS provide higher resolution, enabling detection of submicroscopic copy number variants and point mutations not detected by G-banding, but may miss balanced rearrangements and low-level mosaicism. The International Stem Cell Initiative (www.iscbi.org) has recommended regular genomic assessment, including NGS at least retrospectively, to ensure the safety of iPSC-derived products (Abranches et al., 2020; Andrews et al., 2015; International Stem Cell Initiative, 2018; Kim et al., 2019).

Although expert guidelines and regulatory filings provide insight into acceptable QC strategies (International Society for Stem Cell Research, 2025; Lovell-Badge et al., 2021; Mahalatchimy et al., 2025; Martins and Ribeiro, 2025; Sullivan et al., 2018), the field lacks systematic, empirical evidence on inter-laboratory assay performance. No large-scale benchmarking studies have evaluated reproducibility in flow cytometry or genomic QC for iPSCs, and publicly available comparative datasets remain limited in scope. Without such data, reproducibility remains unverified, interoperability is constrained, and the clinical translation of iPSC-based therapies remains slower and more resource intensive than necessary.

To address this gap, the Global Alliance for iPSC Therapies (GAiT) launched the first coordinated international effort to benchmark two widely used iPSC QC assays: quantitative PCR (qPCR) for genomic integrity and flow cytometry for monitoring the undifferentiated state. Two Quality Assessment Rounds (QARs) were conducted: QAR 2019, organized in collaboration with the CiRA Foundation (CiRA_F), National Institute for Biological Standards and Control (NIBSC), and STEMCELL Technologies, and QAR 2023, conducted jointly with CiRA_F, involving 18 and 23 participating institutes, respectively, together spanning 12 countries. A QAR is a collaborative process through which scientists from different organizations work to ensure that testing practices yield consistent and comparable results. These GAiT QARs build upon the foundation established in the publication "Quality control guidelines for clinical-grade human induced pluripotent stem cell lines" (Sullivan et al., 2018) and are a part of a broader, ongoing international initiative aimed at facilitating the use of iPSC lines as standardized starting materials for cell therapies.

QAR 2019 revealed substantial inter-laboratory variability in flow cytometry results, while the genomic integrity assay showed consistent performance across sites. This variability, despite the use of nominally similar protocols, highlights the inherent challenges of achieving reproducibility through locally adapted practices. Building on these insights, QAR 2023 introduced standardized flow cytometry conditions and kit-based formats to enhance reproducibility and enable systematic evaluation of marker performance across sites and sample states. This

approach enabled the identification of pluripotency-associated markers that are consistently robust, as well as those that exhibit variability in undifferentiated and differentiated contexts.

This study represents the first global, multi-site initiative to generate empirical, data-driven evidence for reproducible iPSC QC assays. Specifically, it evaluates the consistency of flow cytometry-based identity assays and qPCR-based genomic integrity assessments. Unlike previous standardization efforts based primarily on expert consensus, our work provides empirical benchmarks across institutions, revealing marker-level reliability and informing the design of harmonized QC panels that reduce subjectivity and support interoperable workflows. These findings establish a practical foundation for international iPSC banks and bridge the gap between expert guidelines, regulatory-approved QC practices, and the scalable implementation required for global adoption. Ultimately, this work advances the development of harmonized QC frameworks essential for the safe, efficient, and equitable delivery of iPSC-based ATMPs worldwide.

RESULTS

Current testing methods for iPSC lines

QAR 2019

In QAR 2019, participating institutes (hereafter referred to as participants) were surveyed prior to and during the QAR to better understand their testing practices and to inform interpretation of results. All 18/18 (100%) completed the survey. Only 5/18 (28%) reported adopting formal national or international quality standards for iPSC testing, covering areas such as sterility, human pathogen screening, karyology, and transgene integration analyses.

Genetic integrity testing was performed by 14/18 (78%) participants using methods including karyotyping, copy number variation (CNV)/single-nucleotide variant array analysis, DNA methylation profiling, and whole-genome sequencing (WGS). Among these, 6/14 (43%) reported using a standard operating procedure (SOP). Testing was carried out in-house by 8/14 (57%) and externally by 6/14 (43%).

Phenotypic characterization was performed by 14/18 (78%) participants, all of whom reported using both immunocytochemistry and flow cytometry. Of these, 7/14 (50%) confirmed using an SOP for at least one of the methods used. Testing was carried out in-house by 11/14 (79%), and 3/14 (21%) outsourced this work to an external laboratory.

Collectively, these findings indicate that while quality assessment practices are employed by the majority of organizations, there remains a clear need to standardize and validate testing protocols within a robust quality management system to ensure consistency and reliability of iPSC characterization across sites.

QAR 2023

A total of 18/23 participants (78%) completed the survey. Similar to QAR 2019, 4/18 (22%) reported adopting formal national or international quality standards for iPSC testing.

Genetic integrity testing was performed by 13/18 (72%) participants using methods such as single-nucleotide polymorphism (SNP) array, CNV by droplet digital PCR, karyotyping, and WGS. Among these, 9/13 (69%) reported having an SOP in place. Testing was carried out externally by 7/13 (54%), in-house by 4/13 (31%), and by both in-house and external laboratories by 3/13 (23%).

Phenotypic characterization was reported by 12/18 (67%) participants. Of these, 11/12 (92%) used an SOP and performed the testing in-house. Reported approaches included flow cytometry, germ layer assessment by qPCR or reverse-transcription PCR, and immunocytochemistry.

Genotyping was performed by 12/18 (67%) participants using methods such as karyotyping, microarray, WGS, SNP analysis, and NGS. Of these, 8/12 (67%) reported using an SOP, 2/12 (17%) did not, and 2/12 (17%) did not provide this information. Testing was carried out externally by 6/12 (50%), in-house by 5/12 (42%), and by both in-house and external laboratories by 1/12 (8%).

Taken together, these responses show that iPSC testing practices still vary widely between organizations. This lack of consistency highlights the need for standardized protocols and clear regulatory frameworks to ensure the safety, reproducibility, and scalability of future iPSC banks.

Genetic integrity testing with a commercially available kit

QAR 2019 involved participants testing two blinded genomic DNA samples using a commercially available qPCR kit, designed for detection of 8 recurrent genomic abnormalities in human pluripotent stem cell (hPSC) cultures (www.stemcell.com/hpsc-genetic-analysis-kit). The two samples provided to participants were extracted from iPSC cultures that were known to exhibit either a normal diploid karyotype (CiRA G1) or contain an unbalanced structural rearrangement resulting in gain of the long arm of chromosome 1 (1q) (CiRA G2).

A total of 45 individual runs were performed: 11/18 (61%) participants chose to complete three runs each, 5/18 (28%) completed two runs, and 2/18 (11%) completed a single run. Each run consisted of the two genomic DNA samples (CiRA G1 and CiRA G2) and the genomic DNA control supplied with the kit. Participants were asked to interpret their own data and submit both raw data and analyzed results for independent evaluation. All genetic data were anonymized, and handling complied with

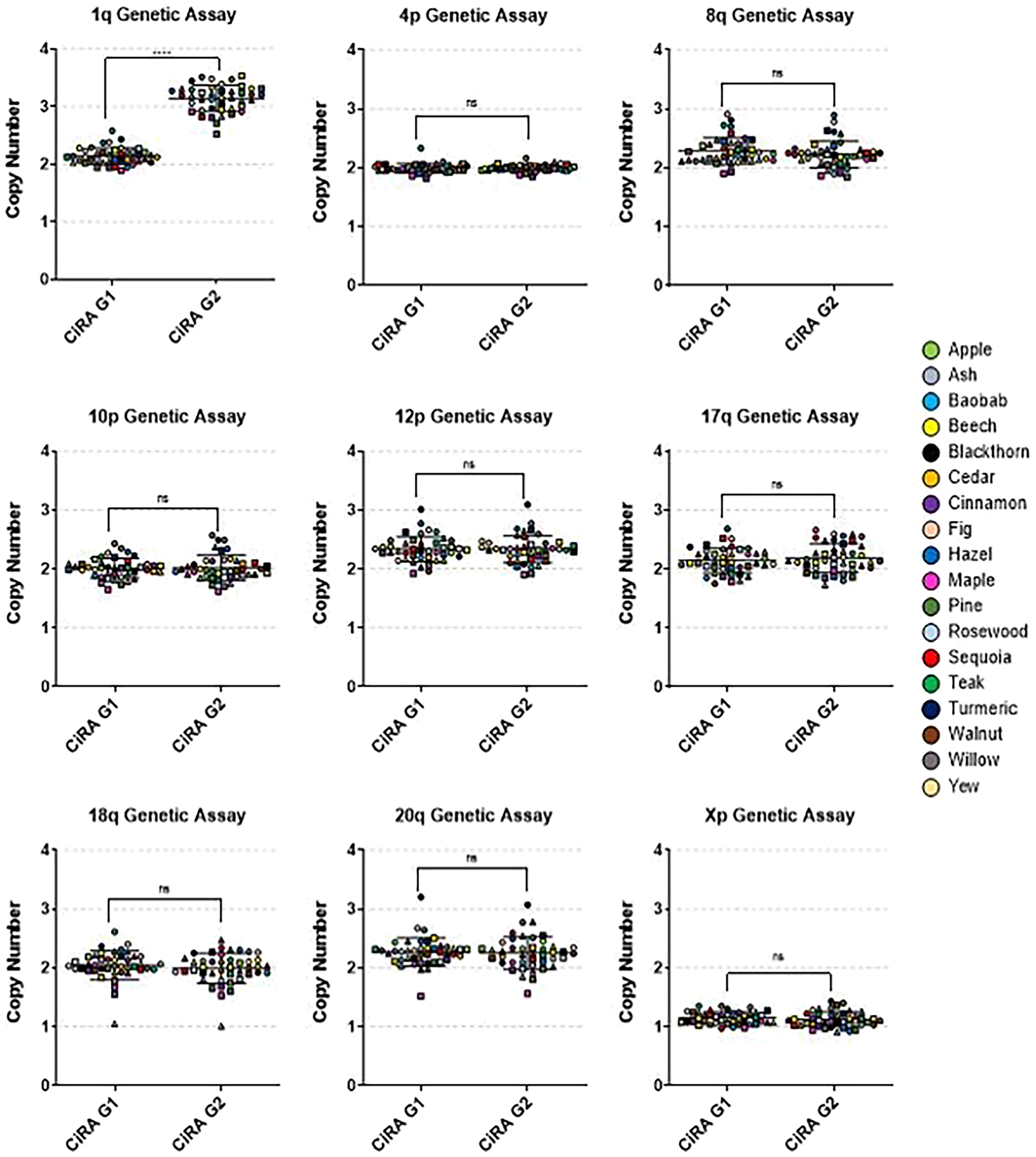

Figure 1. Genetic testing of blinded samples demonstrates broadly consistent results across participants in QAR 2019

Scatterplots show the distribution of copy number data across all valid runs. Each data point represents the median copy number from three technical replicates at a given genomic locus in an individual run. Circles, squares, and triangles denote first, second, and third runs, respectively. Error bars indicate the mean ± standard deviation across all runs for each locus (n = 43).

General Data Protection Regulation and the UK Data Protection Act to protect personal genetic information.

Figure 1 shows the median copy number across each region, calculated from three technical replicates per sample, using data collected from all participants. Of the 45 runs completed, 42 correctly identified the amplification of chromosome 1q in the CiRA G2 sample, while the CiRA G1 sample was accurately classified as diploid. Statistical

analysis showed no significant differences in detected copy number between the CiRA G1 and CiRA G2 samples across all tested loci, except for chromosome 1q, which was consistently amplified in the abnormal sample (paired two-tailed t test, $p \leq 0.0001$).

In the three remaining runs, two were flagged as indeterminate due to high variability between technical replicates. The third was classified as possibly abnormal, as the data were insufficient for the data analysis software to assign a confident result. This was attributable to a lower-than-expected copy number at chromosome 1q (2.52 copies) and the detection of two additional false-positive calls (deletion of chromosomes 10p and 18q). In all three cases, repeat testing would have been recommended.

iPSC characterization by flow cytometry

QAR 2019

In QAR 2019, participants tested five fixed cell samples provided by two independent organizations (CiRA_F and NIBSC), following their routine protocols within the specifications outlined in the QAR 2019 subsection of the methods section. The CiRA_F samples were analyzed by all (18) participants. CiRA F1 represented an undifferentiated sample, and CiRA F2 was a spontaneously differentiated cell sample. All participants also analyzed the NIBSC samples; however, sample preparation issues limited inclusion of data from only four participants whose results met all QC criteria (>60% of total events within the P1 gate [i.e., the first flow cytometry gating region] and a P1 cell count >4,000). Data from participants whose samples failed QC criteria were excluded from further analysis.

To ensure appropriate data grouping, and given that the NIBSC samples were technical replicates, a mixed-effects model was employed to estimate differences between the CiRA F1 and NIBSC samples while accounting variability across markers and participants. The model predicted an average 0.1% difference among the NIBSC triplicates and a 1.5% absolute decrease in %PSC values for the NIBSC samples compared to CiRA F1. Consequently, both datasets were combined for analysis (Figure S1).

Testing of the undifferentiated samples showed reasonable consistency across participants, with most correctly identifying the undifferentiated cell line. In contrast, assessment of the CiRA F2 spontaneously differentiated sample exhibited substantial variability. Among markers, NANOG demonstrated the greatest inter-laboratory variability, whereas OCT3/4 and TRA-1-60 showed the least. The most commonly used markers for iPSC analysis were OCT3/4, TRA-1-60, and SSEA4, followed by TRA-1-81, SOX2, NANOG, and SSEA3.

Given the substantial variability observed in flow cytometry results in the QAR 2019 dataset, we aimed to re-evaluate this aspect under more controlled conditions. Accordingly, QAR 2023 was designed to systematically assess whether standardized flow cytometry protocols and kit-based reagents could improve inter-laboratory reproducibility.

QAR 2023 Quality Test 1 variables

In QAR 2023, three distinct fixed cell sample types (undifferentiated cells, spontaneously differentiated iPSCs, and a 1:1 ratio mixture of both) were analyzed by 23 participants using two separate quality tests. To understand which markers are routinely used in iPSC characterization, participants were first asked to analyze the samples by flow cytometry following their own protocols and reagents (Quality Test 1). Of 23 participants, 22 completed Quality Test 1, with 19/22 (86%) of participants choosing to perform two test runs and 3/22 (14%) choosing to perform one. The number of replicates per run varied, with 8/22 (36%) of runs testing samples once, 6/22 (27%) twice, and 8/22 (36%) three times. Table S1 provides additional details on the number of replicate measurements performed by each participant for each sample.

The number and choice of flow cytometry markers also varied among participants. Ten of 22 (45%) participants analyzed their samples using four different markers, while 8/22 (36%) used three markers (Table S2). The most popular marker within the QAR was OCT 3/4, with 22/22 (100%) of participants selecting this marker for analysis, followed by TRA-1-60 (19/22; 86%), SSEA4 (18/22; 82%), and SOX2 (9/22; 40.9%). The remaining markers were only used by five participants or fewer (Table S2).

The types of flow cytometer used by participants in QAR 2023 varied considerably. A total of 15 different flow cytometer models were used (Table S3), with the BD Accuri C6, BD FACS Canto II, and Miltenyi Biotec MACSQuant Analyzer 10 being the most commonly used.

QAR 2023 Quality Test 1 flow cytometry results

Figure S2 shows each participant's performance in identifying the individual samples, offering insight into the effectiveness of each participant's in-house flow cytometry panel and highlighting the variability observed across contributors. Based on these data, the percentage of cells positive for each marker tested was calculated as the mean across all participants' data (Figure 2). Since the mixed sample combined undifferentiated and differentiated cells at a 1:1 ratio, marker expression was expected to scale proportionally. CD45, SOX2, SSEA4, and TRA-2-49 exhibited low variability between participants; however, their expression levels remained consistent across all sample types, limiting their utility in distinguishing differentiation status.

Conversely, NANOG and TRA-1-81 showed proportional changes in expression correlating with differentiation, decreasing as differentiation increased, but these markers displayed high inter-participant variability. Notably, four

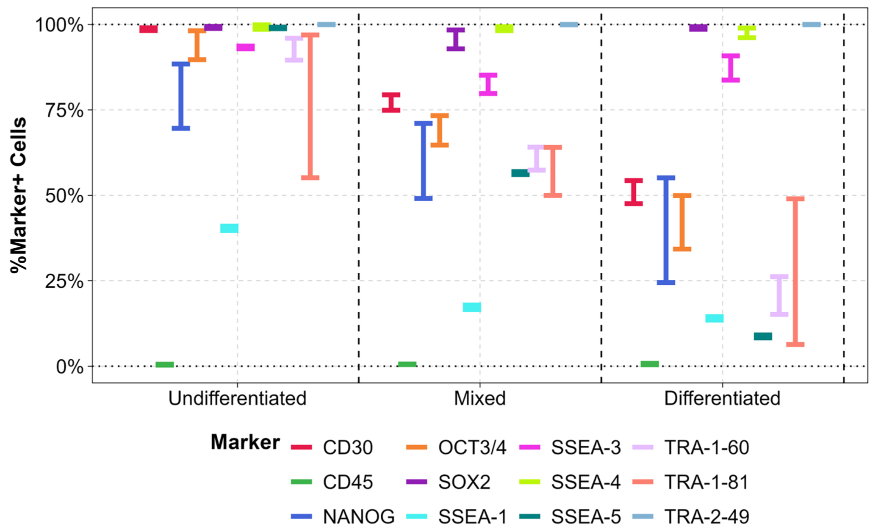

Figure 2. Percentage of marker-positive cells across sample types in QAR 2023 Quality Test 1

Overview of the percentage of marker-positive cells for each sample type (undifferentiated cells, 1:1 ratio mixture, and spontaneously differentiated iPSCs). Each marker is represented by a distinct color. Error bars indicate the 95% confidence interval of the mean, calculated using bootstrapping.

markers, CD30, OCT3/4, SSEA5, and TRA-1-60, combined low inter-participant variability with clear proportional changes in expression across sample types, indicating their suitability for assessing differentiation status.

To further investigate which markers most effectively distinguish differentiated from undifferentiated stem cells, the percentage of marker-positive cells in the differentiated samples was subtracted from that in the undifferentiated samples, yielding the difference in marker expression (Table 1). Markers exhibiting a difference in mean percentage of marker-positive cells greater than 30% were considered suitable for confirming stem cell differentiation. In Quality Test 1, the markers exceeding this threshold included SSEA5, TRA-1-60, TRA-1-81, OCT3/4, CD30, and NANOG.

To evaluate marker consistency, markers showing no change across samples or analyzed by only one participant were excluded. The analysis then focused on four markers: NANOG, OCT3/4, TRA-1-60, and TRA-1-81, which were assessed for within-sample consistency (Table S4). For NANOG, OCT3/4, and TRA-1-60, differentiated cell samples exhibited the highest variability, with standard deviations of 41.4%, 36.4%, and 23.7%, respectively, whereas TRA-1-81 showed greatest variability in undifferentiated samples (33.7%). This variability reflects that some participants failed to detect expected expression changes for certain markers (Figure S2), contributing to the overall data spread.

QAR 2023 Quality Test 2 flow cytometry results

To evaluate whether standardizing staining protocols and reagents could minimize variability among organizations, all 23 participants conducted Quality Test 2. This test utilized standardized flow cytometry samples, reagents (including staining buffers and antibodies conjugated exclusively with Alexa Fluor 488 or fluorescein isothiocyanate [FITC] fluorochromes), and uniform staining and analysis procedures provided by CiRA_F. Figure S3 displays the comprehensive data from all participants for the undifferentiated, mixed, and differentiated samples. The results indicate that all participants, except for one identified as "Swallow," accurately categorized each sample. An anomaly was observed for the participant "Canary," who recorded an unusually high expression of PAX6 in the undifferentiated sample, as illustrated in Figure S3.

To evaluate the effectiveness of the four markers in distinguishing among sample types, recovery of the mixed (1:1) samples was calculated for each participant. The expected recovery was defined as 100% of the theoretical value, derived from the average marker expression in the corresponding undifferentiated and differentiated samples. Figure 3 illustrates these results, while detailed values are provided in Table S5. Overall, 89.7% of results demonstrated recoveries within the range of 80%–125%, indicating that the selected markers performed consistently and proportionally across most participants.

However, as previously observed, the results from "Swallow" were inconsistent across all markers, deviating markedly from the overall trend. This pattern suggests potential issues related to sample handling, staining or analysis protocol execution or the quality of materials used in their analysis. Further investigation revealed that shipment of cell lines and reagents to "Swallow" took 16 days, compared to 0–8 days for the remaining 22 participants (Table S6). This extended transit time may have compromised reagent integrity and, more critically, the quality of the cell samples. Factors such as temperature fluctuations, potential fixative evaporation, or extended storage under suboptimal conditions may have altered cell morphology

Table 1. Mean percentage of marker-positive cells in undifferentiated vs. differentiated samples: Greater discrimination is achieved with a higher value for the difference in the percentage of marker-positive cells

Marker	Mean % marker-positive cells for undifferentiated samples	Mean % marker-positive cells for differentiated samples	Difference in mean % marker-positive cells
SSEA5	99.0	8.8	90.2
TRA-1-60	93.0	20.7	72.4
TRA-1-81	81.1	27.7	53.4
OCT3/4	94.3	41.8	52.5
CD30	98.6	51.1	47.6
NANOG	79.1	38.8	40.3
SSEA1	40.4	14.1	26.3
SSEA3	93.4	87.4	6.0
SSEA4	99.4	97.7	1.7
SOX2	99.1	99.0	0.1
TRA-2-49	100.0	100.0	0.0
CD45	0.4	0.6	-0.2

Markers showing a >30% difference in the mean proportion of marker-positive cells are highlighted in green.

or marker detectability, possibly contributing to the inconsistencies observed in the data from "Swallow."

The mean percentage of cells positive for each of the four markers, TRA-1-60, SSEA4, OCT3/4, and PAX6, was calculated (Figure 4), excluding the data from the participant "Swallow" due to the large number of outliers observed across all samples. For transparency, calculated values both including and excluding the "Swallow" dataset are reported in Table S7. The most variable marker was PAX6, with standard deviations of 6.2% (differentiated), 9.9% (mixed), and 18.2% (undifferentiated), followed by TRA-1-60 with standard deviations of 1.9% (differentiated), 5.5% (mixed), and 6.7% (undifferentiated); OCT3/4 with 4.4% (differentiated), 4.1% (mixed), and 0.5% (undifferentiated); and SSEA4 with 2.8% (differentiated), 2.6% (mixed), and 0.4% (undifferentiated).

As shown in Figures 4 and S4, most participants' data show strong agreement across markers, with only minor exceptions. For the PAX6 marker, a few outliers appear in the undifferentiated and mixed samples, primarily from participant "Hawk." Similarly, for TRA-1-60 in the undifferentiated and mixed samples, outliers originate from participants "Eagle" and "Goldfinch." These outliers are evident when considering the entire dataset. However, as previously shown in Figure 3, individual data from "Hawk," "Eagle," and "Goldfinch" still exhibit clear, proportional shifts in the percentage of marker-positive cells from the undifferentiated to mixed and from mixed to differentiated samples, indicating that flow cytometry results remained consistent and internally coherent for these participants.

The marker SSEA4 was used by 18/22 (82%) participants in Quality Test 1 and was also included as a marker in Quality Test 2. Notably, the level of SSEA4 expression remained high across all analyzed samples. While SSEA4 is one of the markers commonly recommended for inclusion in iPSC QC panels based on expert consensus, and given its widespread use among participants, SSEA4 remains a relevant marker for further investigation. Therefore, a Validation Round was performed to examine marker expressions in greater detail.

Validation Round 2024

Building on the findings from QAR 2023, a Validation Round 2024 was carried out to assess the expression of SSEA4, OCT3/4, TRA-1-60, and SSEA5 in five different iPSC lines differentiated into three germ layers (Figure 5). Cell lines used were provided by five different participants. SSEA4 expression remained high, approaching 100% expression, in all five cell lines at day 0, day 5 endoderm, day 5 mesoderm, and day 7 ectoderm. Consistent with previous findings, OCT3/4 and TRA-1-60 expressions declined rapidly in comparison to SSEA4, with levels decreasing from day 5 of differentiation across all cell lines tested in the Validation Round. SSEA5 expression also showed decreased levels from day 5 of differentiation, with 0% expression observed in the day 5 mesoderm layer and day 7 ectoderm layer samples. In the day 5 endoderm samples,

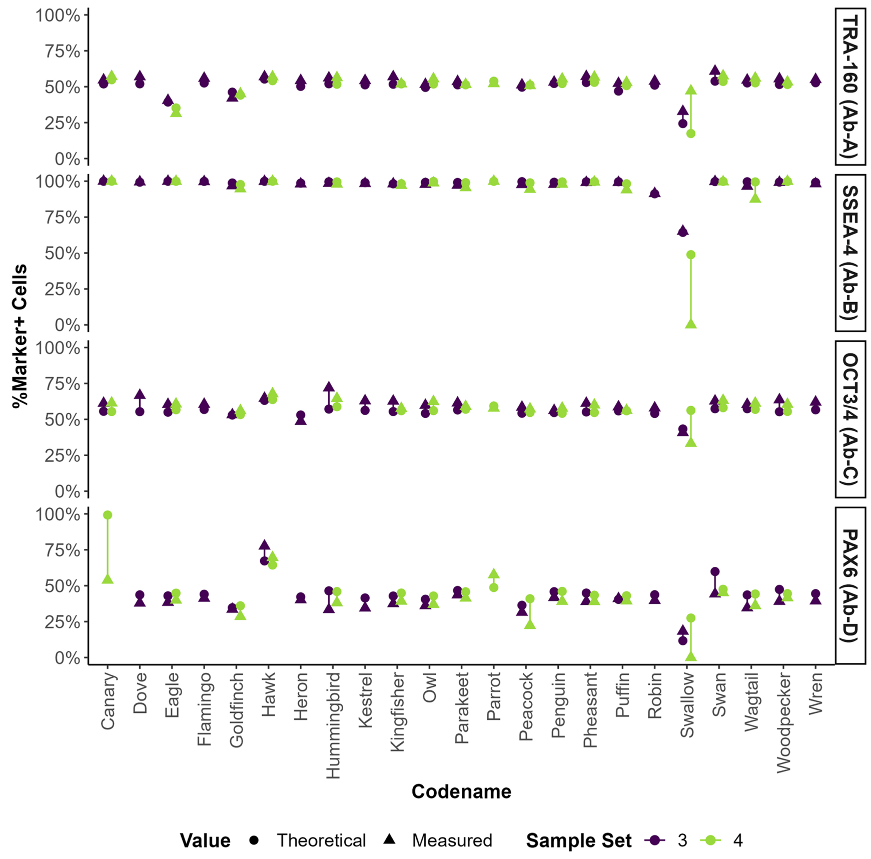

Figure 3. Expected versus observed marker expression in mixed samples in QAR 2023 Quality Test 2

Discrepancies between expected and observed levels of TRA-1-60, SSEA4, OCT3/4, and PAX6 are shown for each participant. The expected recovery represents the precise midpoint between undifferentiated and differentiated samples, reflecting the theoretical marker expression in a 1:1 mixture. Observed measurements are compared to this theoretical value to evaluate marker proportionality and participant performance.

OCT3/4, TRA-1-60, and SSEA5 expression was slightly higher than in other lineages, likely due to the lower efficiency of endoderm differentiation, yet still markedly lower than SSEA4. The reduced endoderm differentiation efficiency in this experiment is supported by lineage-specific marker expression in Figure S5.

To assess whether expression levels of SSEA4 decline over time, we applied long-term differentiation protocols toward otic progenitor cells (12 days), cardiomyocytes (15 days), dopaminergic progenitor cells (12 days), and dopaminergic neurons (26 days). These cell types were selected based on established in-house protocols routinely used within our laboratories. Validation of cell type-specific marker expression is provided in Figure S5.

As shown in Figure 5, a progressive decrease in SSEA4 expression was observed across most cell types over time, with some variability between cell lines. Mean SSEA4 expression levels ranged approximately between 50% and 60% for cardiomyocyte and dopaminergic progenitor cell differentiations. In dopaminergic neurons, expression was notably lower, with a mean below 10%. In contrast, otic progenitor cells maintained elevated SSEA4 expression levels even after 12 days of differentiation.

Expressions of TRA-1-60, OCT3/4, and SSEA5 remained consistently low, typically between 0 and 10%, across all long-term differentiation conditions. The exception was observed in otic progenitor cells, where OCT3/4 and SSEA5 showed slightly elevated levels, with mean expression values around 10%, with OCT3/4 remaining just below and SSEA5 slightly above this value.

DISCUSSION

This international benchmarking initiative represents a significant step toward resolving a key challenge in regenerative medicine: the lack of harmonized standards for iPSC QC. Through two coordinated QARs (QAR 2019 and QAR 2023), involving 18 and 23 participating sites, respectively, across 12 countries and five continents, we engaged a diverse range of institutions, including academic centers, biotechnology companies, government-funded iPSC facilities, and medical research charities. By evaluating interlaboratory consistency in flow cytometry-based identity assays and qPCR-based genomic integrity assessments, this study provides the first global, multi-site dataset of

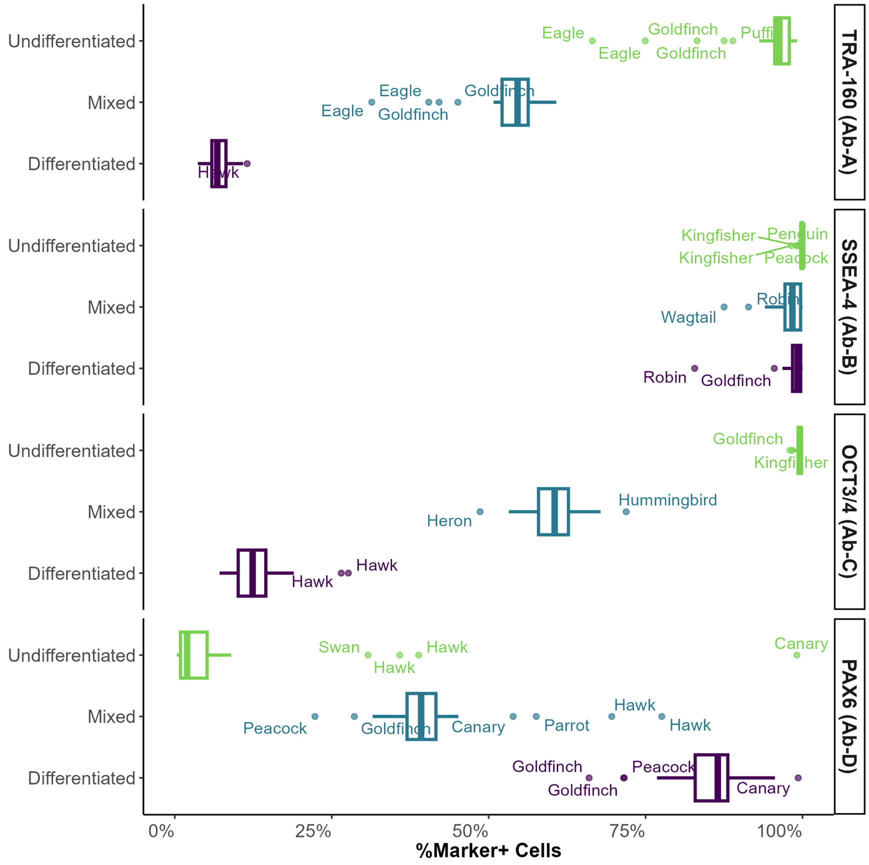

Figure 4. Mean percentage of marker-positive cells by sample type in QAR 2023 Quality Test 2

The mean percentage of cells expressing TRA-1-60, SSEA4, OCT3/4, and PAX6 across undifferentiated (green), mixed (blue), and differentiated (purple) sample types, including standard deviation, are shown. Participant codenames are highlighted for results that deviate most significantly from the median. Data from the participant codenamed "Swallow" have been excluded from this analysis.

empirical evidence for reproducible iPSC QC. Our findings highlight markers and workflows that deliver the most consistent results, offering a practical foundation for standardization and global interoperability.

Genetic testing across the network

In QAR 2019, the qPCR kit and accompanying analysis software demonstrated high-quality, largely consistent data. Some variability in procedures and occasional analysis errors was observed, underscoring the need for appropriate controls and replicates. Both the kit and the software were specifically designed and developed as a diagnostic for fast and simple in-house testing of hPSC cultures. The kit manufacturer reports that the assay can detect culture abnormalities that are present in at least 30% of the population, similar to the sensitivity of other genetic testing methods (Baker et al., 2016). This qPCR-based method targets nine genetic regions, eight of which are known regions of recurrent karyotypic abnormalities in hPSC cultures (Baker et al., 2016). It was not, however, designed to replace more extensive karyotypic analysis, and the need for more informative methods for assessing genetic integrity remains an active area of discussion (Jo et al., 2020). At present, no single approach can provide full identification of all abnormalities that may arise during reprogramming or extended culture of iPSCs (O'Shea et al., 2020). Therefore, it is strongly encouraged to combine G-banding karyotype with a molecular method such as array comparative genomic hybridization or SNP array and WGS.

Flow cytometry analysis across the network

Flow cytometry characterizations in QAR 2019, using participants' standard protocols, revealed substantial variability due to diverse reagents, staining combinations, and instrumentation. Co-staining with OCT3/4 and TRA-1-60 was the most common and reliable combination, although sample preparation issues led to some failed analyses. To address these limitations, workshops were held prior to QAR 2023 to harmonize protocols, and a GO/NO-GO step was introduced to exclude samples with insufficient cell numbers or events.

In QAR 2023, two testing formats were applied: Quality Test 1 used participants' own protocols, while Quality Test 2 employed standardized samples, reagents, and harmonized procedures. In Quality Test 1, 19/22 (86.4%) participants successfully distinguished the three sample

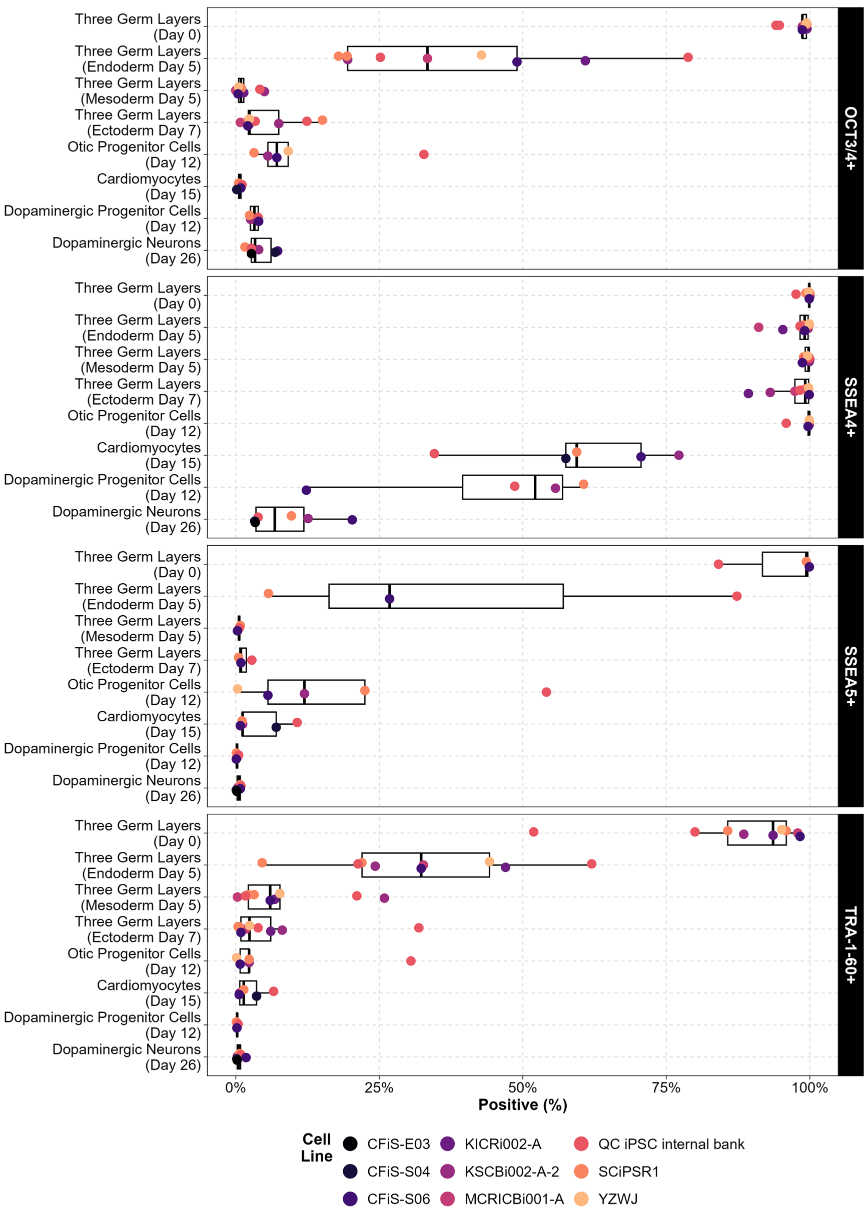

Figure 5. Marker-positive cell percentages across differentiated iPSC lines in Validation Round 2024

Average percentages of cells positive for TRA-1-60, SSEA4, OCT3/4, and SSEA5 are shown for five iPSC lines differentiated into three germ layers, endoderm (day 5), mesoderm (day 5), and ectoderm (day 7), and into specific cell types, otic progenitor cells (day 12), cardiomyocytes (day 15), dopaminergic progenitor cells (day 12), and dopaminergic neurons (day 26). Bars represent mean percentages ±SD.

types (undifferentiated iPSCs, spontaneously differentiated iPSCs, and a 1:1 mixture). Collectively, OCT3/4, TRA-1-60, and SSEA5 emerged as the top markers, showing both low inter-participant variability and clear proportional expression changes across sample types, underscoring their suitability for assessing differentiation status. Notably, an even higher proportion of participants (22/23, 96%) correctly distinguished the sample types in the standardized Quality Test 2, illustrating the benefit of harmonization. Only one participant generated inconsistent results, most likely due to prolonged shipment times, which may have compromised sample integrity. Minor outliers in the TRA-1-60 and PAX6 datasets still followed clear proportional trends across sample types, demonstrating that accurate classification remained feasible despite variations in gating. Although refined gating could further improve inter-participant alignment, the existing settings were sufficient to distinguish the samples, albeit with a narrower dynamic range and reduced sensitivity to differences in marker expression.

Results from QAR 2023 also showed that SSEA4 remained highly expressed across all samples. In the Validation Round 2024, five iPSC lines were assessed for SSEA4, SSEA5, TRA-1-60, and OCT3/4 expression during differentiation into four different cell types (cardiomyocytes, otic progenitors, dopaminergic progenitors, and dopaminergic

neurons) as well as into progenitors of all three germ layers. SSEA4 showed the slowest decline, while SSEA5, TRA-1-60, and OCT3/4 were downregulated more rapidly. These results suggest that SSEA4 is more robustly and persistently expressed in iPSCs, making it less suitable for tracking early differentiation events. However, its stability may be advantageous as a sensitive marker for detecting residual pluripotent cells in differentiated products, particularly in QC settings where detection of uncommitted cells is critical.

Future considerations

This study focused primarily on flow cytometry-based identity assays and qPCR-based genomic integrity screening. Future QARs could extend benchmarking to additional technologies and differentiation contexts, enabling evaluation of functional assays and marker performance across a wider range of cell types. Such efforts would further strengthen the evidence base for reproducible practices and support the development of comprehensive QC frameworks.

Based on insights from QAR 2019, QAR 2023, and the Validation Round 2024, we propose a preliminary roadmap for implementing future multi-site benchmarking of emerging iPSC QC technologies. Key components of such a benchmarking strategy include: (1) establishment of well-defined reference materials matched to the analytical platform (e.g., iPSC lines with predefined genomic or phenotypic characteristics); (2) co-development of harmonized protocols through workshops and pilot testing, incorporating clear GO/NO-GO criteria to minimize procedural variability; and (3) implementation of shared or standardized data analysis pipelines to reduce software-dependent variability (e.g., in the context of flow cytometry, gating-dependent differences between sites).

Incorporation of these elements can shorten the overall timeline of international benchmarking initiatives by reducing common failure modes, enabling earlier detection of workflow deviations, and streamlining data interpretation across participating sites. To support future efforts, we outline below a proposed roadmap for international multi-site benchmarking.

1. Preparation phase
   - Selection and pre-validation of reference materials
   - Definition of assay scope, test parameters, and performance metrics
2. Harmonization phase
   - Cross-site workshops and protocol alignment
   - Pilot testing to identify sources of variability
   - Definition of GO/NO-GO acceptance criteria
3. Benchmarking phase
   - Distribution of blinded samples
   - Multi-site testing using agreed workflows
   - Standardized data capture and reporting formats
4. Analysis and feedback phase
   - Centralized or harmonized data analysis
   - Inter-site performance comparison
   - Identification of potential sources of variability and protocol refinement

A critical next step for enabling global adoption of harmonized iPSC QC practices is engagement with regulatory authorities. While this study provides empirical evidence to benchmark assay reproducibility and identify robust markers, translating these findings into accepted standards will require coordinated dialog with regulators. International organizations such as GAiT, the International Society for Stem Cell Research, and the International Society for Cell & Gene Therapy are actively engaged in developing standards and guidelines for pluripotent stem cell therapies. Building on this role, they are well positioned to lead regulatory engagement by leveraging global networks and collaborative platforms.

Conclusion

Despite two decades of iPSC research, harmonized QC standards remain limited, hindering the scalable manufacture of iPSC-derived ATMPs. This global study integrates standardized assays with empirical benchmarking across multiple institutes to generate data-driven evidence for reproducible QC protocols and reliable marker panels. Our findings support the inclusion of OCT3/4, TRA-1-60, and SSEA5 as robust markers for monitoring the undifferentiated status of iPSCs, together with harmonized flow cytometry workflows that apply clear GO/NO-GO thresholds and validated gating strategies to minimize subjectivity. For genomic integrity, we recommend combining G-banding karyotype with genome-wide molecular assays, while using qPCR as a complementary tool for routine screening. This multi-tiered approach balances accuracy, scalability, and feasibility for global implementation.

Standardization is more than a technical exercise; it reduces resource burden, aligns regulations, and enables scalable, interoperable workflows. Sustained international collaboration and continued coordinated efforts will be essential to advance shared quality standards, strengthen supply chains, and deliver high-quality, affordable iPSC-derived therapies globally.

METHODS

Quality Assessment Round survey

Participants in each study were provided with a survey to document and better understand their routine iPSC testing practices. Complete QAR 2019 and QAR 2023 questionnaires are provided in Document S1. The survey

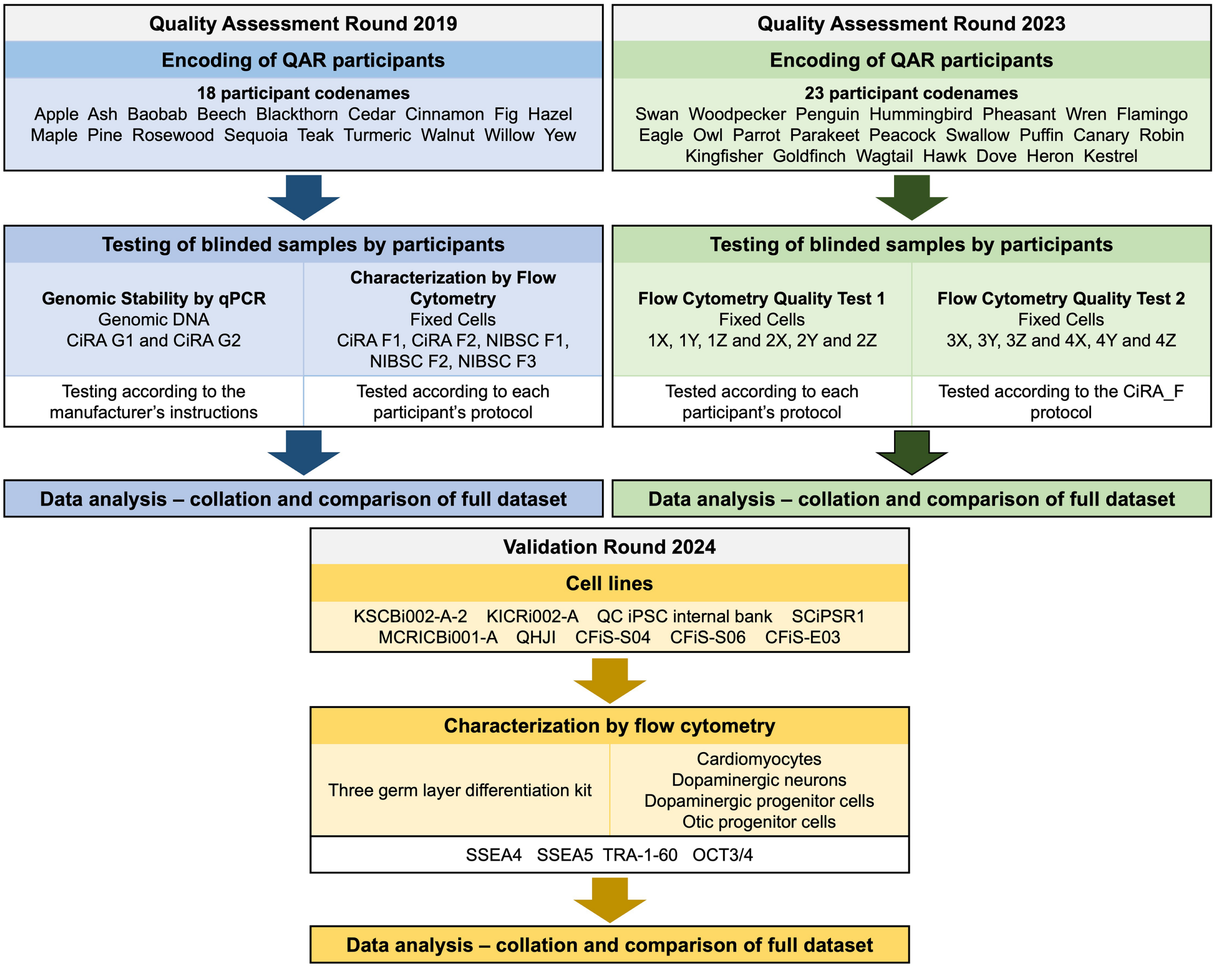

Figure 6. QAR design flow diagram

The main stages of each QAR and the Validation Round, including lists of the participants' codenames.

included questions covering the donor management process, infectious agent testing, sterility testing, genotyping, phenotyping, characterization, and safety testing. Participants were also asked to indicate which procedures were governed by SOPs and whether testing was conducted in-house or outsourced to external laboratories. The qualitative data were collated and summarized as the percentage of total responses to each question.

Quality Assessment Round design

QAR 2019 (Figure 6): 18 participants were asked to assess the genetic integrity of two genomic DNA samples (CiRA G1 and CiRA G2; provided by CiRA_F) using a commercially available qPCR kit. In parallel, participants analyzed five fixed cell samples (CiRA F1, CiRA F2, NIBSC F1, NIBSC F2, and NIBSC F3; provided by CiRA_F and NIBSC) by flow cytometry, applying their established laboratory protocols and routinely used antibodies to confirm an undifferentiated, iPSC phenotype. Each participant independently analyzed their own datasets, submitting pseudonymized results to GAiT under a unique codename. GAiT subsequently collated and analyzed the complete QAR 2019 dataset.

QAR 2023 (Figure 6): Participants were asked to analyze an identical set of samples provided by CiRA_F using two distinct flow cytometry quality tests. For Quality Test 1, 22 participants tested fixed cell samples (1X, 1Y, and 1Z and 2X, 2Y, and 2Z) following their own laboratory protocols and employing the antibodies routinely used to verify a differentiated, iPSC phenotype. Each participant analyzed the data independently, according to their own procedures and software tools. For Quality Test 2, 23 participants tested fixed cell samples (3X, 3Y, and 3Z and 4X, 4Y, and 4Z) using a standardized protocol and antibody panel provided by CiRA_F. Each participant analyzed the data independently according to the supplied instructions but using their own

analysis software. Pseudonymized results were submitted to GAiT under assigned codenames. GAiT subsequently collated and analyzed the complete QAR 2023 dataset.

Validation Round 2024 (Figure 6): Five participants submitted cell lines to CiRA_F for a focused validation round comparing the expression of SSEA4, SSEA5, TRA-1-60, and OCT3/4.

Quality Assessment Round and Validation Round samples and reagents

Detailed information on the samples and reagents used in QAR 2019, QAR 2023, and Validation Round 2024 is provided in Document S1. Document S1 also contains the full instructions distributed to participants for QAR 2019 and QAR 2023. The use of human iPSC lines in this study was approved by the Kyoto University Graduate School and Faculty of Medicine Ethics Committee, approval number R3218.

Quality Assessment Round testing and analysis

QAR 2019

QAR 2019 genomic stability by qPCR: Samples were thawed and tested using the hPSC genetic analysis kit (STEMCELL Technologies, Cat. No. 07550). Individual datasets were analyzed by participants using the associated data analysis software (STEMCELL Technologies hPSC Genetic Analysis Tool v.1.0.1: 2018-11-13).

QAR 2019 characterization by flow cytometry: Participants were required to test the supplied flow cytometry samples with a minimum of two markers from the standard undifferentiated panel (OCT3/4, TRA-1-60, TRA-1-81, SSEA3, SSEA4, SOX2, or NANOG) as they normally would as part of their in-house iPSC QC testing programs. A combination of at least one intracellular (e.g., OCT3/4, SOX2, or NANOG) and one extracellular (e.g., SSEA4 or TRA-1-60, TRA-1-81) marker was also required. Analysis for additional markers, which comprise participant's routine iPSC quality testing, were also to be shared. When performing nuclear staining, participants were asked to also use their own standard permeabilization protocols. Participants analyzed their own individual dataset using their internal procedures and provided results as the percentage of cells positive for each marker tested.

QAR 2023

QAR 2023 flow cytometry Quality Test 1: Participants were required to test the supplied cell samples (1X, 1Y, and 1Z and 2X, 2Y, and 2Z) with a minimum of two markers from the standard undifferentiated panel (OCT3/4, TRA-1-60, TRA-1-81, SSEA3, SSEA4, SOX2, or NANOG) as they normally would as part of their in-house iPSC QC testing programs. A combination of at least one intracellular (e.g., OCT3/4, SOX2, or NANOG) and one extracellular (e.g., SSEA4, TRA-1-60, or TRA-1-81) marker was also required. Analysis for additional markers, which comprise participant's routine iPSC quality testing, were also to be shared. When performing nuclear staining, participants were asked to also use their own standard permeabilization protocols. Participants analyzed their own individual dataset using their internal procedures and provided results as the percentage of cells positive for each marker tested.

QAR 2023 flow cytometry Quality Test 2: Participants were required to test samples (3X, 3Y, and 3Z and 4X, 4Y, and 4Z) using the provided single staining protocol and reagents. Cells were thawed, added to 5 mL of buffer, and divided into five individual tubes. Cells were centrifuged at 200 $\times$ g for 5 min and resuspended in 30 μL of Ab-A, Ab-B, Ab-C, Ab-D, or Ab-U (buffer only). Cells were incubated in the dark at room temperature for 15 min, mixed, and then incubated for a further 15 min. 1 mL of buffer was added to each tube, and cells were centrifuged at 200 $\times$ g for 5 min. Cells were resuspended in 250 μL of buffer, filtered through a nylon mesh strainer, and stored at 4°C in the dark until analysis. Acquisition instructions included the removal of doublet populations and analysis of cells positive for FITC using the unstained sample as the negative control. Results were provided as the percentage of cells positive for each marker tested.

Data analysis

Participants submitted their individual analyzed datasets to GAiT. These datasets were collated by quality round, and the results were compared across all participants. Data were managed and shared in accordance with FAIR and CARE principles to ensure accessibility, interoperability, and ethical use. In QAR 2019, participants were assigned codenames based on tree species, and in QAR 2023, bird species. These distinctive labels enabled each organization to recognize its own results without compromising anonymity and reduced confusion compared with random alphanumeric identifiers, particularly during follow-up discussions and comparative analyses. For clarity and continuity, these codenames are retained in this manuscript.

RESOURCE AVAILABILITY

Lead contact

Requests for further information and resources should be directed to and will be fulfilled by the lead contact, Anna Falk (anna.falk@med.lu.se).

Materials availability

This study did not generate new unique reagents. All iPSC lines used in QAR 2019 and QAR 2023 were pre-existing lines established at CiRA_F. Experimental samples, including spontaneously differentiated and fixed cell preparations, were generated solely

for inter-laboratory assessment and are not maintained as distributable reagents.

For Validation Round 2024, five participating institutions submitted pre-existing iPSC lines to CiRA_F for centralized analysis. In accordance with institutional agreements and ownership by the submitting institutions, these Validation Round 2024 cell lines are not available for redistribution.

All instructions, workflows and survey materials used in the QARs are provided in Document S1.

Data and code availability

All flow cytometry data, qPCR data, and survey-derived metadata reported in this study will be shared by the lead contact upon request. This study does not include any data types requiring deposition in a specialized repository, and it does not report original code. Any additional information required to reanalyze the data reported in this paper is available from the lead contact upon request.

ACKNOWLEDGMENTS

Funding for the QAR 2023 and Validation Round 2024 was provided by the Circulation Foundation through a grant from the Japanese Ministry of Health, Labour and Welfare (MHLW). This work was supported by Korea National Institute of Health project 2020-NG-016-00 (H.Y.C.), Vinnova Innovation Milieus (IndiCell 2021-02695 [A.F.] and StartCell 2024-01124 [A.F.]) funded by the Swedish Agency for Innovation, the Lund Stem Cell Center (A.F.), LU ATMP Centrum (A.F.), SFO StemTherapy (A.F.), and the National ATMP Research School, funded by the Swedish Research Council (A.H.). The authors thank Marie Jönsson for support with the graphical abstract and acknowledge the Cell and Gene Technology Core and the FACS Core at the Lund Stem Cell Center.

AUTHOR CONTRIBUTIONS

Conceptualization, A.L.M., J.B., J.M., S. Sullivan, and M.T.; methodology, A.L.M., J.B., A.G., J.M., A.J.H., S. Sullivan, and M.T.; formal analysis, D.M. and A.J.H.; investigation, S. Sakamoto, A.H., K.A., E.A., B.A., R.B., R.A.Q.B., A.B.C., D.C., H.Y.C., M.C., B.A.C., S.C., S.J.D., N.E., X.F., M. Feyeux, M. Forrester, S.M.G., A.J.H., A.L.H., L.G.J., R.N.J., Y.K., T.H.K.-B., J.-H.K., M.K., S.K., B.K., C.Y.L., W.L., M.R.M., M.M., L.M.-A., O.M., S.L.M., H. Munir, S.H.S.N., H. Ogawa, S.O., N.P., L.P.V., I.P.d.S.B., A.R.P., S.Q., J.R., A.R., S. Sullivan, R.S., K.S., S. Selvitella., G. Singh, M.J.K.S., M.S., C.T., P.T., E.U., J.M.A.V., A.V., J.W.V.W., K.W.-C., J.W., A.Y., and G.Z.; resources: A.F., A.H., A.L.M., H.C., S.C., N.E., A.G., H.-Y.H., T.H., J.-H.K., K.O., J.M., and P.T.; writing – original draft, A.H., R.W., A.J.H., and S. Sullivan; writing – review & editing: A.F., A.H., A.L.M., E.A., R.B., J.B., D.C., S.J.D., N.E., M. Forrester, J.-H.K., M.M., L.M., S.H.S.N., J.S., G. Stacey, S. Sullivan, M.T., and P.T.; supervision, A.L.M., A.B.G., A.C.C.d.C., A.F., H.C., H.Y.C., B.A.C., R.N.J., H.K., A.M., J.M., H. Ohara, A.R., M.J.K.S., and G. Stacey; project administration, H. Mamiya, R.W., and S. Sullivan; funding acquisition, A.L.M. and T.H.

DECLARATION OF INTERESTS

H.C., M.R.M., O.M., and X.F. are employed by the Center for Commercialization of Regenerative Medicine (CCRM). G.Z. was formerly employed by CCRM. A.F. is the CSO of CCRM Nordic. R.B. is employed by SmartCella. A.R.P., D.M., J.B., M.J.K.S., S.K., and W.L. are employed by Cell and Gene Therapy Catapult. E.A. is the CSO of ViSync. S.J.D. is employed by FUJIFILM Cellular Dynamics Inc. K.S., L.M.-A., and S.M.G. are employed by TreeFrog Therapeutics. M. Feyeux is a co-founder, shareholder, member of the Strategic Committee, and employee of TreeFrog Therapeutics. A.G., A.J.H., A.L.H., J.W.V.W., M. Forrester, and R.N.J. are employed by STEMCELL Technologies. J.R. is employed by BlueRock Therapeutics. L.G.J. was employed by BlueRock Therapeutics (2019) and Notch Therapeutics (2023/2024). C.Y.L. is employed by Century Therapeutics. A.M. is employed by Dark Horse Consulting Group. M.M. is and S.L.M. was employed by Cellino, S.L.M. is listed on patents 12195765 B2, US 11931737 B2, and US11708563 B2. S.H.S.N. was employed by Notch Therapeutics and is currently an independent consultant. H. Ohara. and S. Sakamoto are employed by Sumitomo Pharma Co., Ltd. S. Sullivan is employed by Lindville Bio. E.U. is employed by BioLamnia. J.S. is a founder of iPS Bio, Inc.

SUPPLEMENTAL INFORMATION

Supplemental information can be found online at https://doi.org/10.1016/j.stemcr.2026.102857.

Received: November 27, 2025
Revised: February 17, 2026
Accepted: February 18, 2026
Published: March 19, 2026

REFERENCES

Abranches, E., Spyrou, S., and Ludwig, T. (2020). GMP Banking of Human Pluripotent Stem Cells: A US and UK perspective. Stem Cell Res. *45*, 101805. https://doi.org/10.1016/j.scr.2020.101805.

Andrews, P.W., Baker, D., Benvinisty, N., Miranda, B., Bruce, K., Brüstle, O., Choi, M., Choi, Y.M., Crook, J.M., de Sousa, P.A., et al. (2015). Points to consider in the development of seed stocks of pluripotent stem cells for clinical applications: International Stem Cell Banking Initiative (ISCBI). Regen. Med. *10*, 1–44. https://doi.org/10.2217/rme.14.93.

Andrews, P.W., Ben-David, U., Benvenisty, N., Coffey, P., Eggan, K., Knowles, B.B., Nagy, A., Pera, M., Reubinoff, B., Rugg-Gunn, P.J., and Stacey, G.N. (2017). Assessing the Safety of Human Pluripotent Stem Cells and Their Derivatives for Clinical Applications. Stem Cell Rep. *9*, 1–4. https://doi.org/10.1016/j.stemcr.2017.05.029.

Avery, S., Hirst, A.J., Baker, D., Lim, C.Y., Alagaratnam, S., Skotheim, R.I., Lothe, R.A., Pera, M.F., Colman, A., Robson, P., et al. (2013). BCL-XL mediates the strong selective advantage of a 20q11.21 amplification commonly found in human embryonic stem cell cultures. Stem Cell Rep. *1*, 379–386. https://doi.org/10.1016/j.stemcr.2013.10.005.

Baker, D., Hirst, A.J., Gokhale, P.J., Juarez, M.A., Williams, S., Wheeler, M., Bean, K., Allison, T.F., Moore, H.D., Andrews, P.W., and Barbaric, I. (2016). Detecting Genetic Mosaicism in Cultures of Human Pluripotent Stem Cells. Stem Cell Rep. *7*, 998–1012. https://doi.org/10.1016/j.stemcr.2016.10.003.

Barry, J., Hyllner, J., Stacey, G., Taylor, C.J., and Turner, M. (2015). Setting Up a Haplobank: Issues and Solutions. Curr. Stem Cell Rep. *1*, 110–117. https://doi.org/10.1007/s40778-015-0011-7.

Escribá, R., Beksac, M., Bennaceur-Griscelli, A., Glover, J.C., Koskela, S., Latsoudis, H., Querol, S., and Alvarez-Palomo, B. (2024). Current Landscape of iPSC Haplobanks. Stem Cell Rev. Rep. *20*, 2155–2164. https://doi.org/10.1007/s12015-024-10783-7.

Halliwell, J., Barbaric, I., and Andrews, P.W. (2020). Acquired genetic changes in human pluripotent stem cells: origins and consequences. Nat. Rev. Mol. Cell Biol. *21*, 715–728. https://doi.org/10.1038/s41580-020-00292-z.

International Society for Stem Cell Research (2025). Best practices for the development of pluripotent stem cell-derived therapies. International Society for Stem Cell Research. October 2025. https://www.isscr.org/translational-best-practices.

International Stem Cell Initiative, Amps, K., Andrews, P.W., Anyfantis, G., Armstrong, L., Avery, S., Baharvand, H., Baker, J., Baker, D., Munoz, M.B., et al. (2011). Screening ethnically diverse human embryonic stem cells identifies a chromosome 20 minimal amplicon conferring growth advantage. Nat. Biotechnol. *29*, 1132–1144. https://doi.org/10.1038/nbt.2051.

International Stem Cell Initiative (2018). Assessment of established techniques to determine developmental and malignant potential of human pluripotent stem cells. Nat. Commun. *9*, 1925. https://doi.org/10.1038/s41467-018-04011-3.

Jo, H.Y., Han, H.W., Jung, I., Ju, J.H., Park, S.J., Moon, S., Geum, D., Kim, H., Park, H.J., Kim, S., et al. (2020). Development of genetic quality tests for good manufacturing practice-compliant induced pluripotent stem cells and their derivatives. Sci. Rep. *10*, 3939. https://doi.org/10.1038/s41598-020-60466-9.

Kim, J.H., Alderton, A., Crook, J.M., Benvenisty, N., Brandsten, C., Firpo, M., Harrison, P.W., Kawamata, S., Kawase, E., Kurtz, A., et al. (2019). A Report from a Workshop of the International Stem Cell Banking Initiative, Held in Collaboration of Global Alliance for iPSC Therapies and the Harvard Stem Cell Institute, Boston, 2017. Stem Cell. *37*, 1130–1135. https://doi.org/10.1002/stem.3003.

Kirkeby, A., Main, H., and Carpenter, M. (2025). Pluripotent stem-cell-derived therapies in clinical trial: A 2025 update. Cell Stem Cell *32*, 10–37. https://doi.org/10.1016/j.stem.2024.12.005.

Lovell-Badge, R., Anthony, E., Barker, R.A., Bubela, T., Brivanlou, A.H., Carpenter, M., Charo, R.A., Clark, A., Clayton, E., Cong, Y., et al. (2021). ISSCR Guidelines for Stem Cell Research and Clinical Translation: The 2021 update. Stem Cell Rep. *16*, 1398–1408. https://doi.org/10.1016/j.stemcr.2021.05.012.

Mahalatchimy, A., Roby, V., Véran, J., Chabannon, C., and Sabatier, F. (2025). Mapping the European landscape and specificity of ATMPs guidance. Cytotherapy *27*, 1262–1269. https://doi.org/10.1016/j.jcyt.2025.06.008.

Martins, F., and Ribeiro, M.H.L. (2025). Quality and Regulatory Requirements for the Manufacture of Master Cell Banks of Clinical Grade iPSCs: The EU and USA Perspectives. Stem Cell Rev. Rep. *21*, 645–679. https://doi.org/10.1007/s12015-024-10838-9.

Merkle, F.T., Ghosh, S., Kamitaki, N., Mitchell, J., Avior, Y., Mello, C., Kashin, S., Mekhoubad, S., Ilic, D., Charlton, M., et al. (2017). Human pluripotent stem cells recurrently acquire and expand dominant negative P53 mutations. Nature *545*, 229–233. https://doi.org/10.1038/nature22312.

O'Shea, O., Steeg, R., Chapman, C., Mackintosh, P., and Stacey, G.N. (2020). Development and implementation of large-scale quality control for the European bank for induced Pluripotent Stem Cells. Stem Cell Res. *45*, 101773. https://doi.org/10.1016/j.scr.2020.101773.

Peterson, S.E., and Loring, J.F. (2014). Genomic instability in pluripotent stem cells: implications for clinical applications. J. Biol. Chem. *289*, 4578–4584. https://doi.org/10.1074/jbc.R113.516419.

Sullivan, S., Stacey, G.N., Akazawa, C., Aoyama, N., Baptista, R., Bedford, P., Bennaceur Griscelli, A., Chandra, A., Elwood, N., Girard, M., et al. (2018). Quality control guidelines for clinical-grade human induced pluripotent stem cell lines. Regen. Med. *13*, 859–866. https://doi.org/10.2217/rme-2018-0095.

Weissbein, U., Peretz, M., Plotnik, O., Yanuka, O., Sagi, I., Golan-Lev, T., and Benvenisty, N. (2019). Genome-wide Screen for Culture Adaptation and Tumorigenicity-Related Genes in Human Pluripotent Stem Cells. iScience *11*, 398–408. https://doi.org/10.1016/j.isci.2018.12.031.

World Health Organization (2018). WHO Expert Committee on Biological Standardization: sixty-ninth report. https://www.who.int/publications/i/item/9789241210256.

Wuputra, K., Ku, C.C., Wu, D.C., Lin, Y.C., Saito, S., and Yokoyama, K.K. (2020). Prevention of tumor risk associated with the reprogramming of human pluripotent stem cells. J. Exp. Clin. Cancer Res. *39*, 100. https://doi.org/10.1186/s13046-020-01584-0.

Stem Cell Reports, Volume *21*

Supplemental Information

Toward standardized iPSC testing: Insights from a multi-year international Quality Assessment Round

Alice Hägg, Rachel Wood, Ayako L. Mochizuki, Keren Abberton, Elsa Abranches, Belén Alvarez-Palomo, Ricardo Baptista, Raiana Andrade Quintanilha Barbosa, Jacqueline Barry, Adriana Bastos Carvalho, Annelise Bennaceur Griscelli, Antonio Carlos Campos de Carvalho, Diana Chaker, Hong Chang, Hye Young Choi, Margarita Codinach, Begoña Arán Corbella, Scott Cowan, Sarah Jane Dickerson, Ngaire Elwood, Xueling Fan, Maxime Feyeux, Maddy Forrester, Andrew Gaffney, Solenn M. Guilbert, Hye-Yeong Ha, Adam J. Hirst, Arwen L. Hunter, Leanne G. Jamieson, Robert N. Judson, Yonehiro Kanemura, Tais Hanae Kasai-Brunswick, Jung-Hyun Kim, Howard Kim, Manisha Kintali, Siddharth Krishnan, Bernd Kuebler, Chui Yu Lau, Wilson Li, Amanda Mack, Michael R. MacLeod, Marinna Madrid, Hiroaki Mamiya, Lucie Manache-Alberici, Dragoş Mărginean, Olivier Mentre, Stefanie L. Morgan, Joanne Mountford, Humayun Munir, Siemon H.S. Ng, Haruna Ogawa, Steve Oh, Hidetaka Ohara, Keiko Oono, Niall Park, Lygia V. Pereira, Izabella Pereira da Silva Bezerra, Alexandru Robert Podovei, Sergio Querol, Jainy Raje, Angel Raya, Satoko Sakamoto, Raquel Sarafian, Kathleen Schmit, Silvia Selvitella, Gurbind Singh, Matthew J.K. Smart, Jihwan Song, Glyn Stacey, Stephen Sullivan, Miho Sumida, Cecile Terrenoire, Pei Tian, Elias Uhlin, José M.A. Vaquero, Anna Veiga, Jar Wei Vicky Wang, Katherine Warre-Cornish, Jamie Wood, Atsuyo Yamamoto, Gaojun Zhang, Takafusa Hikichi, Marc Turner, and Anna Falk

Supplemental Information

Supplemental Figures

Figure S1. QAR 2019 flow cytometry analysis of marker expression across sample types.

Figure S2. QAR 2023 Quality Test 1 flow cytometry analysis of marker expression across sample types.

Figure S3. QAR 2023 Quality Test 2 flow cytometry analysis of marker expression across sample types.

Figure S4. Distribution of marker-specific data in QAR 2023 Quality Test 2 across different sample types, related to Figure 4.

Figure S5. Validation of cell type-specific marker expression in Validation Round 2024, related to Figure 5.

Supplemental Tables

Table S1. Number of replicate measurements performed by each participant for each sample in QAR 2023 Quality Test 1.

Table S2. Number of participants using each marker in QAR 2023 Quality Test 1.

Table S3. Flow cytometers used by each participant in QAR 2023.

Table S4. Consistency of marker expression between sample types in QAR 2023 Quality Test 1.

Table S5. Percentage recovery of mixed samples based on expected values in QAR 2023 Quality Test 2, related to Figure 3.

Table S6. Duration of cell line and reagent shipment (in days) for each participant in QAR 2023.

Table S7. The mean percentage of cells positive for TRA-1-60, SSEA4, OCT3/4 and PAX6 across the undifferentiated, mixed and differentiated sample types, related to Figure 4.

Supplemental Experimental Procedures

Supplemental Tables related to Supplemental Experimental Procedures

Table S8. Description of samples provided for QAR 2019 and QAR 2023.

Table S9. Description of cell lines and expression tests in Validation Round 2024.

Table S10. iPSC culture media used in the Validation Round 2024.

Table S11. Cell culture coatings used in the Validation Round 2024.

Table S12. Primary and secondary antibodies used in the Validation Round 2024.

Supplemental Methods

Methods S1. QAR 2019 participant survey

Methods S2. QAR 2023 participant survey

Methods S3. QAR 2019 instructions to participants

Methods S4. QAR 2023 Quality Test 1 and 2 instructions to participants

Supplemental Figures

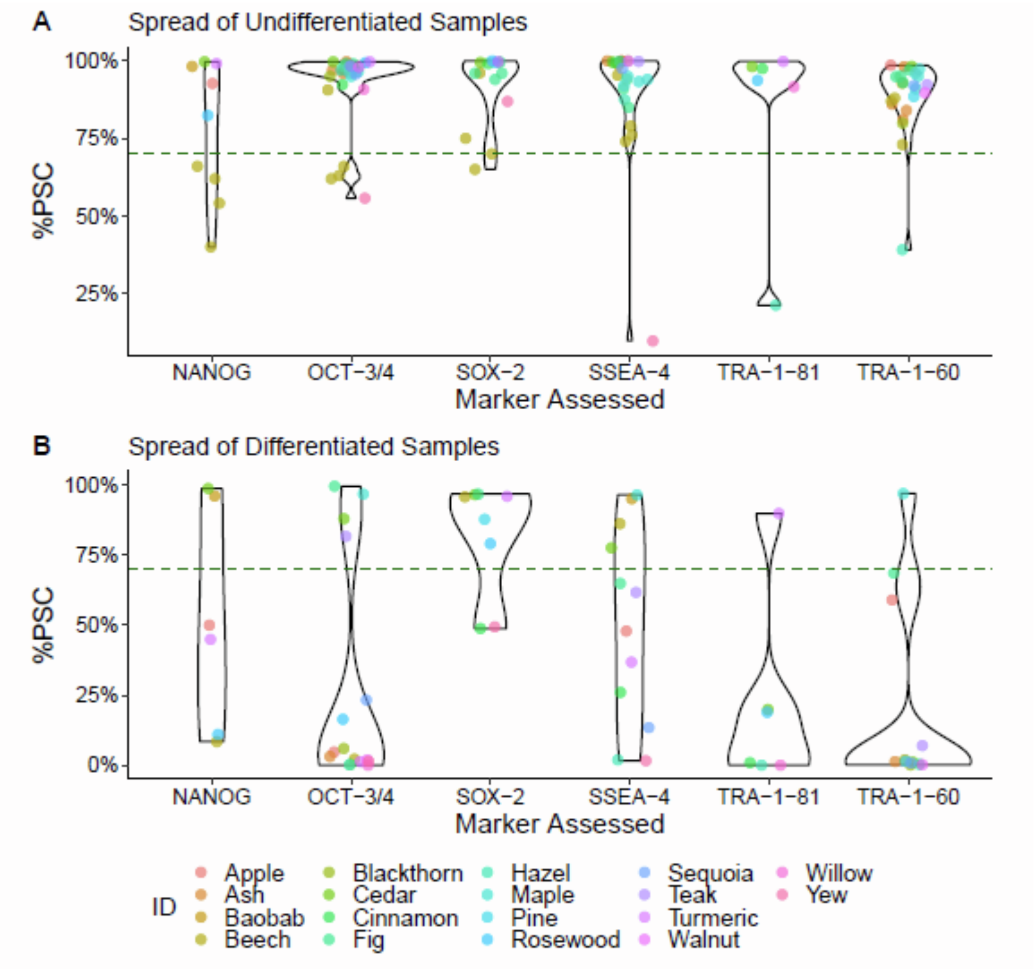

Figure S1. QAR 2019 flow cytometry analysis of marker expression across sample types. Violin plots of all samples that passed the QC criteria. A) shows the data from the undifferentiated CiRA and NIBSC samples, while B) presents data for the spontaneously differentiated CiRA F2 sample. The green dotted line indicates the 70% undifferentiated marker expression threshold, representing the minimum level established in the GAiT QC guidelines (Sullivan et al., 2018). Multiple data points from participants “Beech” and “Fig” reflect repeated analyses performed by these laboratories on the same samples.

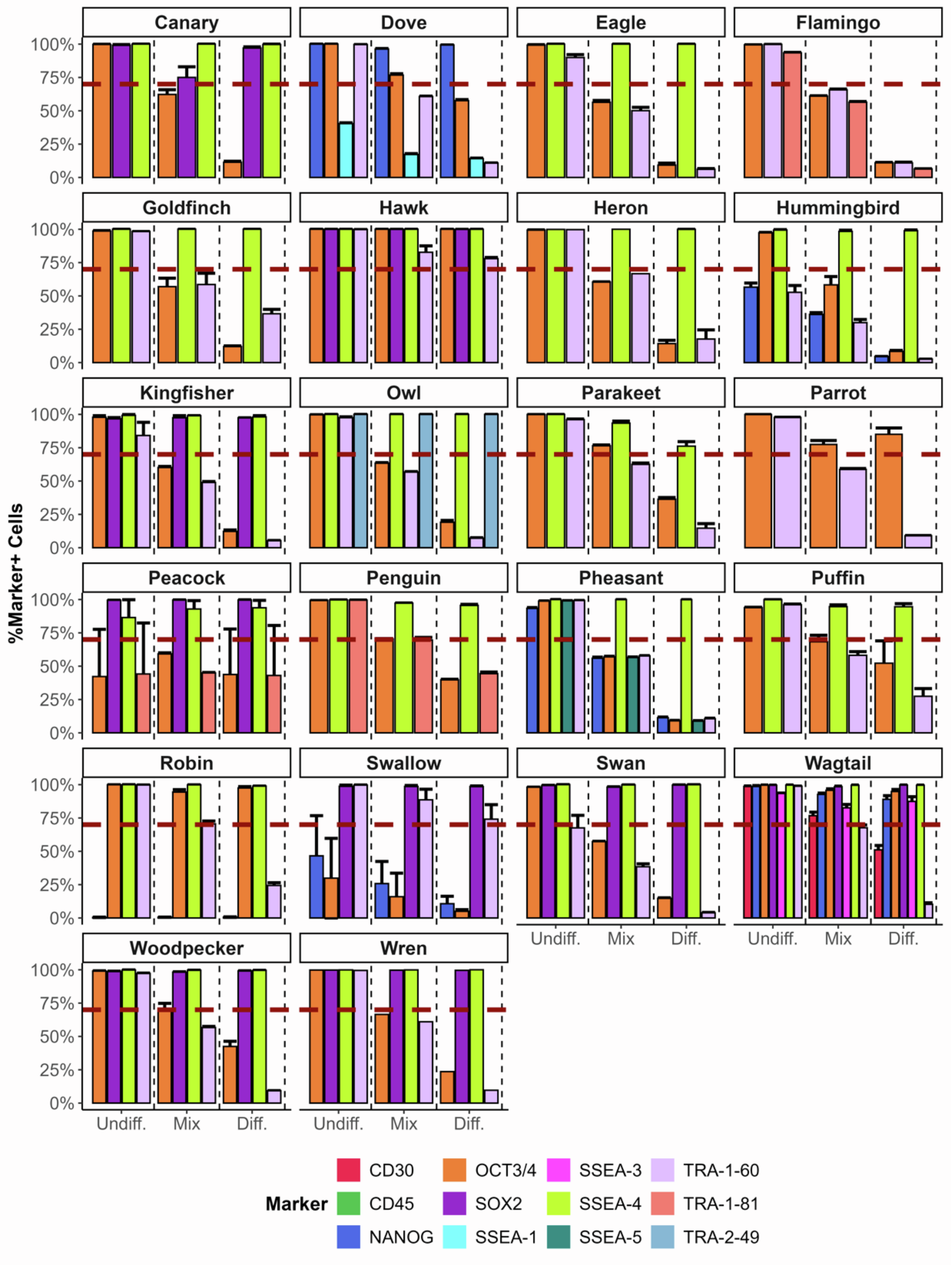

Figure S2. QAR 2023 Quality Test 1 flow cytometry analysis of marker expression across sample types. Percentage of marker-positive cells in each sample type (undifferentiated iPSCs, 1:1 mixed sample, and spontaneously differentiated iPSCs) analysed in Quality Test 1. Results are shown separately for each participant. Each marker is colour coded. Error bars indicate the standard deviation across replicate measurements within each participant's run.

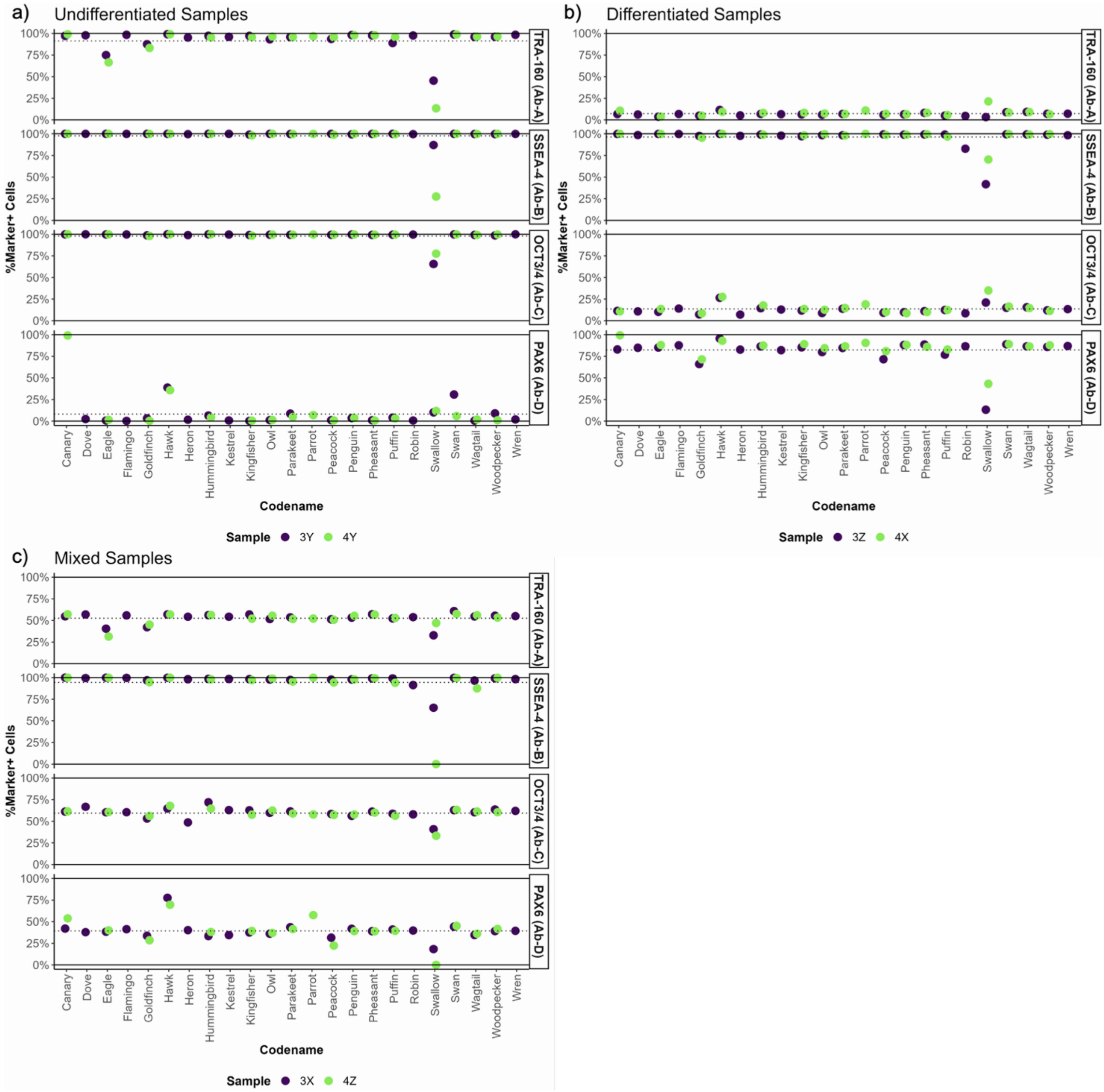

Figure S3. QAR 2023 Quality Test 2 flow cytometry analysis of marker expression across sample types. TRA-1-60, SSEA4, OCT3/4, and PAX6 expression in (a) undifferentiated iPSCs, (b) spontaneously differentiated iPSCs, and (c) a 1:1 mixture of undifferentiated and differentiated cells. Individual points represent each participant's results, and dotted lines indicate the global average for each marker.

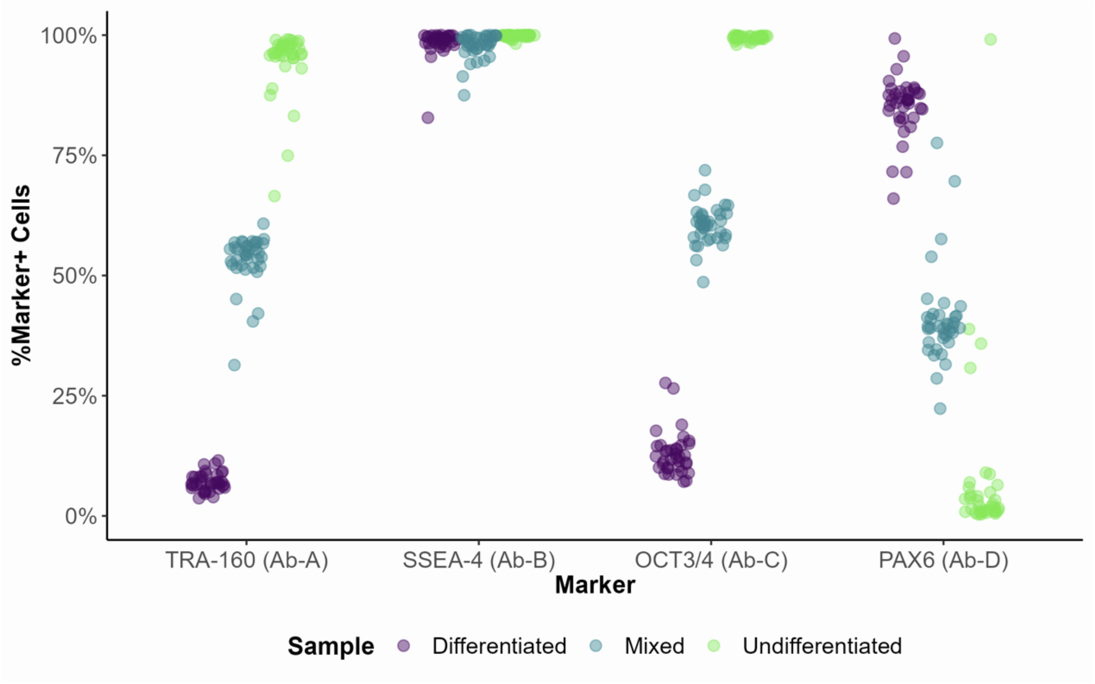

Figure S4. Distribution of marker-specific data in QAR 2023 Quality Test 2 across different sample types, related to Figure 4. Expression levels of TRA-1-60, SSEA4, OCT3/4, and PAX6 were analysed. The different sample types are denoted by following colours: purple for differentiated samples, blue for mixed samples (1:1 ratio), and green for undifferentiated samples. Note that data from the participant identified as "Swallow" have been excluded from this figure for clarity.

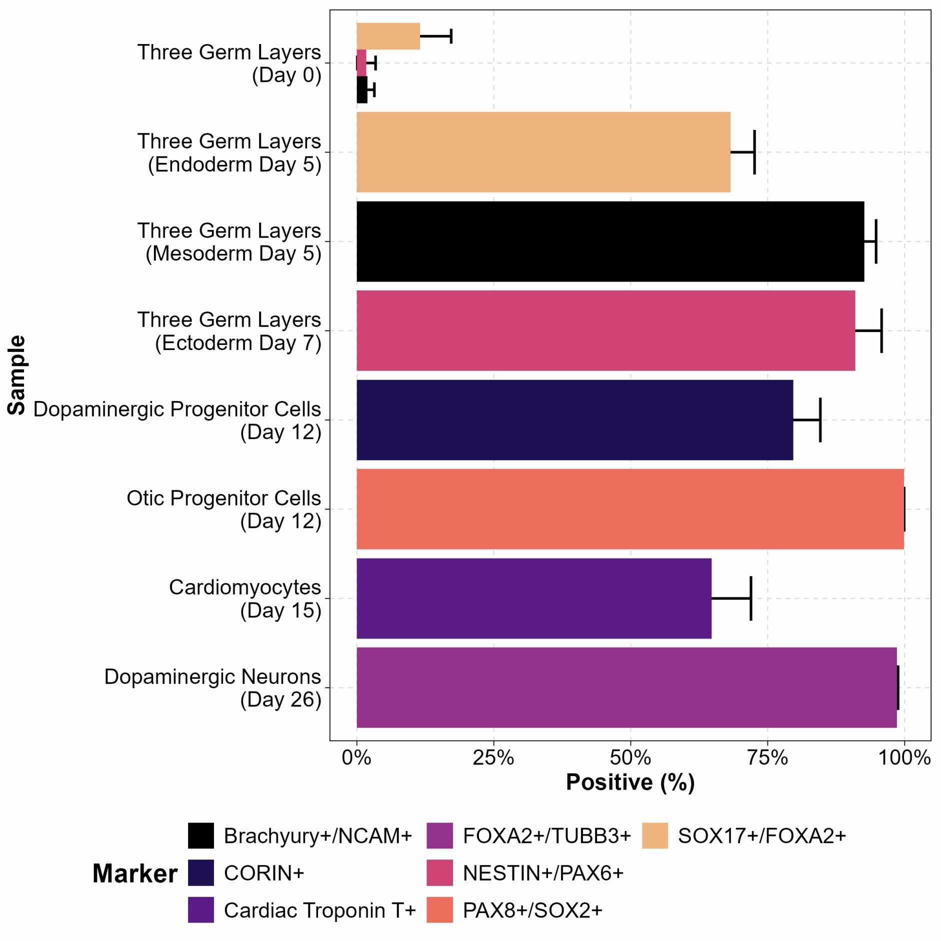

Figure S5. Validation of cell type-specific marker expression in Validation Round 2024, related to Figure 5. Overview of marker expression assessed by flow cytometry during directed differentiation of iPSCs. Samples were collected at key stages: undifferentiated iPSCs (Day 0), endoderm (Day 5), mesoderm (Day 5), ectoderm (Day 7), dopaminergic progenitor cells (Day 12), otic progenitor cells (Day 12), cardiomyocytes (Day 15), and dopaminergic neurons (Day 26).

Supplemental Tables

Table S1. Number of replicate measurements performed by each participant for each sample in QAR 2023 Quality Test 1. Two samples were provided for each condition: undifferentiated (1X, 2Z), differentiated (1Y, 2X), and a 1:1 mixed sample (1Z, 2Y).

	Undifferentiated		Differentiated		Mixed	
**Codename**	**1X**	**2Z**	**1Y**	**2X**	**1Z**	**2Y**
Canary	2	2	2	2	2	2
Dove	3	-	3	-	3	-
Eagle	2	2	2	2	2	2
Flamingo	3	-	3	-	3	-
Goldfinch	1	1	1	1	1	1
Hawk	3	3	3	3	3	3
Heron	1	1	1	1	1	1
Hummingbird	3	3	3	3	3	3
Kingfisher	1	1	1	1	1	1
Owl	2	2	2	2	2	2
Parakeet	2	2	2	2	2	2
Parrot	2	2	2	2	2	2
Peacock	1	1	1	1	1	1
Penguin	1	1	1	1	1	1
Pheasant	3	3	3	3	3	3
Puffin	1	1	1	1	1	1
Robin	3	3	3	3	3	3
Swallow	2	2	2	2	2	2
Swan	1	1	1	1	1	1
Wagtail	3	3	3	3	3	3
Woodpecker	3	3	3	3	3	3
Wren	1	-	1	-	1	-

Table S2. Number of participants using each marker in QAR 2023 Quality Test 1. The table lists all markers together with the participant codenames who reported including them in their in-house flow cytometry panels, as indicated by the green dots. The final row summarizes the total number of participants using each marker.

Codename	OCT3/4	TRA-1-60	SSEA4	SOX2	NANOG	TRA-1-81	SSEA5	TRA-2-49	CD45	CD30	SSEA3	SSEA1
Swan	●	●	●	●								
Woodpecker	●	●	●	●								
Penguin	●		●			●						
Hummingbird	●	●	●		●							
Pheasant	●	●	●		●		●					
Wren	●	●	●	●								
Eagle	●	●	●									
Flamingo	●	●				●						
Owl	●	●	●					●				
Parrot	●	●										
Parakeet	●	●	●									
Peacock	●		●	●		●						
Swallow	●	●		●	●							
Puffin	●	●	●									
Canary	●		●	●								
Kingfisher	●	●	●	●								
Robin	●	●	●						●			
Goldfinch	●	●	●									
Wagtail	●	●	●	●	●					●	●	
Hawk	●	●	●	●								
Dove	●	●			●							●
Heron	●	●	●									
**Number of Participants (n)**	22	19	18	9	5	3	1	1	1	1	1	1

Table S3. Flow cytometers used by each participant in QAR 2023. The table lists the instrument name, manufacturer, and the participants that reported using each flow cytometer. The final column shows the percentage of all participants using each instrument.

Flow cytometer used	Manufacturer	Codename	Number of participants (n)
BD FACSAria III	BD Biosciences	Goldfinch	1
Attune NxT	Thermo Fisher	Penguin	1
BD Accuri C6	BD Biosciences	Hummingbird<br>Swallow<br>Canary	3
BD Accuri C6+	BD Biosciences	Kestrel	1
BD FACSCanto II	BD Biosciences	Owl<br>Puffin<br>Dove	3
BD FACSLyric	BD Biosciences	Swan	1
BD LSRFortessa	BD Biosciences	Wren<br>Woodpecker	2
CytoFLEX LX	Beckman Coulter	Kingfisher	1
BD FACSVerse	BD Biosciences	Eagle	1
GALLIOS	Beckman Coulter	Peacock	1
Guava easyCyte 12HT	Cytek Biosciences	Parrot	1
BD LSRII	BD Biosciences	Flamingo	1
MACSQuant Analyzer 10	Miltenyi Biotec	Pheasant<br>Parakeet<br>Wagtail	3
MACSQuant Analyzer 16	Miltenyi Biotec	Hawk<br>Robin	2
NAVIOS EX	Beckman Coulter	Heron	1

Table S4. Consistency of marker expression between sample types in QAR 2023 Quality Test 1. Within-sample consistency for NANOG, OCT3/4, TRA-1-60 and TRA-1-81 was calculated by the mean percentage of marker-positive cells and standard deviation.

**Marker**	**Sample type**	**Mean % marker positive cells**	**Standard deviation (%)**
NANOG	Undifferentiated	79.1	± 25.7
	Mixed	60.0	± 28.7
	Differentiated	38.8	± 41.4
OCT3/4	Undifferentiated	94.3	± 19.4
	Mixed	69.3	± 19.8
	Differentiated	41.8	± 36.4
TRA-1-60	Undifferentiated	93.0	± 14.0
	Mixed	60.7	± 14.8
	Differentiated	20.7	± 23.7
TRA-1-81	Undifferentiated	81.1	± 33.7
	Mixed	56.9	± 10.2
	Differentiated	27.7	± 29.5

Table S5. Percentage recovery of mixed samples based on expected values in QAR 2023 Quality Test 2, related to Figure 3. The expected values are calculated from the results of undifferentiated and differentiated samples. Results within the range of 80% to 125% recovery are highlighted in green, while those below 80 or above 125 are highlighted in red.

Codename	Sample set	PAX6 (Ab-D) (%)	OCT3/4 (Ab-C) (%)	TRA-1-60 (Ab-A) (%)	SSEA4 (Ab-B) (%)
Canary	3	0.00	110.10	105.30	100.10
	4	129.40	277.00	292.00	100.00
Dove	3	86.90	120.50	109.40	100.20
Eagle	3	89.50	109.80	102.90	100.00
	4	89.20	107.00	89.10	100.00
Flamingo	3	93.80	106.40	106.20	99.80
Goldfinch	3	97.00	100.40	91.10	98.00
	4	79.40	105.30	102.30	96.90
Hawk	3	115.40	102.30	102.90	99.90
	4	108.10	106.30	104.90	100.00
Heron	3	95.20	91.70	108.10	99.40
Hummingbird	3	72.00	125.80	107.90	98.90
	4	83.00	109.90	108.90	98.50
Kestrel	3	83.20	111.90	105.90	99.40
Kingfisher	3	87.50	113.10	110.10	100.00
	4	87.40	102.80	100.00	98.80
Owl	3	89.00	110.40	104.20	98.60
	4	86.30	111.30	107.00	99.00
Parakeet	3	93.50	108.60	104.50	98.10
	4	90.50	103.40	100.60	96.50
Parrot	4	118.20	97.50	96.90	100.00
Peacock	3	86.60	107.70	103.20	98.00
	4	54.50	104.70	99.10	95.40
Penguin	3	91.20	102.70	101.80	98.50
	4	85.00	106.60	106.20	98.40
Pheasant	3	86.90	111.00	107.90	99.50
	4	89.70	109.60	106.90	99.60
Puffin	3	101.40	105.00	111.40	99.50
	4	91.60	100.60	104.20	95.70
Robin	3	91.00	107.10	105.40	100.20
Swallow	3	156.70	94.20	134.70	101.20
	4	0.00	59.20	270.40	0.00
Swan	3	74.00	109.40	112.90	100.00
	4	95.10	108.80	107.30	99.70
Wagtail	3	79.40	105.20	103.80	96.90
	4	81.60	107.60	106.30	87.90
Woodpecker	3	82.50	115.00	107.70	99.80
	4	93.60	109.00	103.50	100.10
Wren	3	88.60	109.40	104.20	99.00

Table S6. Duration of cell line and reagent shipment (in days) for each participant in QAR 2023.

Codename	Shipment time (days)
Owl	0
Penguin	4
Parrot	3
Eagle	1
Parakeet	4
Hummingbird	5
Woodpecker	8
Wren	5
Peacock	8
Swallow	16
Swan	4
Wagtail	4
Puffin	4
Hawk	7
Canary	4
Dove	5
Flamingo	6
Goldfinch	7
Pheasant	5
Kestrel	1
Kingfisher	5
Heron	6
Robin	6

Table S7. The mean percentage of cells positive for TRA-1-60, SSEA4, OCT3/4 and PAX6 across the undifferentiated, mixed and differentiated sample types, related to Figure 4. Mean and standard deviations are shown in columns 3 and 4. Columns 5 and 6 represent the same calculations with the outlier data set from participant "Swallow" removed.

Sample type	Marker	Mean % marker positive cells	Standard deviation (%)	Mean % marker positive cells (outlier removed)	Standard deviation (%) (outlier removed)
Differentiated	TRA-1-60 (Ab-A)	7.30	3.00	7.10	1.90
	SSEA4 (Ab-B)	96.30	10.40	98.50	2.80
	OCT3/4 (Ab-C)	13.70	5.70	12.90	4.40
	PAX6 (Ab-D)	82.30	14.50	85.20	6.20
Mixed	TRA-1-60 (Ab-A)	52.50	6.30	53.20	5.50
	SSEA4 (Ab-B)	94.50	16.60	97.80	2.60
	OCT3/4 (Ab-C)	59.30	6.60	60.50	4.10
	PAX6 (Ab-D)	39.30	12.20	40.90	9.90
Undifferentiated	TRA-1-60 (Ab-A)	91.30	16.40	94.60	6.70
	SSEA4 (Ab-B)	97.60	11.70	99.80	0.40
	OCT3/4 (Ab-C)	98.00	6.40	99.40	0.50
	PAX6 (Ab-D)	8.20	17.70	8.10	18.20

Supplemental Experimental Procedures

All quality round samples were labelled in a format to remove bias from participant's testing and analysis (Table S8), sample information was released to participants at the completion of each Quality Round.

Table S8. Description of samples provided for QAR 2019 and QAR 2023.

**QAR 2019 sample label**	
CiRA G1	Genomic DNA from a Ff-I03 iPSC clone for which no common abnormality
CiRA G2	Genomic DNA extracted from Ff-I13 iPSC clone which tested positive for a Chr 1q abnormality
CiRA F1	Undifferentiated and self-renewing Ff-I01s04 iPSC line
CiRA F2	Spontaneously differentiated iPSC culture: differentiated and predominantly non-self-renewing cells Ff-I01s04DIFF (cells differentiated from Ff-I01s04 iPSC line by 10 days culture in the medium without FGF2)
NIBSC F1	Undifferentiated, self-renewing NIBSC8 iPSC line (1st technical replicate)
NIBSC F2	Undifferentiated, self-renewing NIBSC8 iPSC line (2nd technical replicate)
NIBSC F3	Undifferentiated, self-renewing NIBSC8 iPSC line (3rd technical replicate)
**QAR 2023 sample label**	
1X, 2Z, 3Y, 4Y	Fixed undifferentiated cells
1Y, 2X, 3Z, 4X	Fixed differentiated cells
1Z, 2Y, 3X, 4Z	Fixed 1:1 ratio mixture of undifferentiated and differentiated cells

QAR 2019 cell samples

iPSC lines (Ff-I03, Ff-I13 and Ff-I01s04) were established from healthy human peripheral blood mononuclear cells (PBMCs) with episomal vectors in CiRA_F. iPSCs were cultured on Laminin-511 E8 fragment coated plates in StemFit (Ajinomoto, AK03N） under 5% CO2 at 37°C. To introduce spontaneous cell differentiation, iPSCs were cultured in AK03N without reagent C for 10 days. All cell samples were fixed with fixation buffer (BD Biosciences, Cat. No. 554655). Fixed cells were cryopreserved in STEM-CELLBANKER® GMP grade (Zenogen Pharma, Cat. No. 11924) and stored in the vapor phase of a liquid nitrogen tank. Samples were distributed using a dry shipper with continuous temperature monitoring. Clonal chromosomal aberration of CiRA G2 (Ff-I13) was confirmed by Karyotyping.

QAR 2023 cell samples

The iPSC line QHJI was derived from healthy human PBMCs and established using episomal vectors in CiRA_F. iPSCs were maintained on Laminin-511 E8 fragment coated plates in StemFit® AK03N medium (Ajinomoto) under 5% CO_2 at 37 °C. For spontaneous cell differentiation, iPSCs were cultured in AK03N medium without reagent C for 10 days. All cell samples were fixed using Cytofix Fixation Buffer (BD Biosciences, Cat. No. 554655). Sample labeling was randomised for participant testing. Fixed cells were cryopreserved in STEM-CELLBANKER® GMP grade (Zenogen Pharma, Cat. No. 11924) and stored in the vapor phase of a liquid nitrogen tank. Samples were distributed using a dry shipper with continuous temperature monitoring.

QAR 2023 flow cytometry reagents supplied to participants

Participants were provided with a flow cytometry buffer (Perm/Wash™ Buffer, BD Biosciences, Cat. No. 554723) and four antibodies: Ab-A, Alexa Fluor® 488 Mouse anti-Human TRA-1-60 (BD Biosciences, Cat. No. 560173); Ab-B, SSEA4 Antibody, anti-Human, FITC, REAfinity® (Miltenyi Biotec, Cat. No. 130-122-918); Ab-C, Alexa Fluor® 488 Mouse anti-Oct3/4 (BD Biosciences, Cat. No. 560253); and Ab-D, Alexa Fluor® 488 Mouse anti-Human PAX6 (BD Biosciences, Cat. No. 561664).

QAR 2019 participant survey and instructions

The complete QAR 2019 participant survey and the experimental instructions are provided in Methods S1 and Methods S3, respectively.

QAR 2023 participant survey and instructions

The complete QAR 2023 participant survey and the experimental instructions are provided in Methods S2 and Methods S4, respectively.

Validation Round 2024 cells and reagents

Table S9. Description of cell lines and expression tests in Validation Round 2024.

Cell line name	Reference	Expression tests
KSCBi002-A-2 (hFSiPS3-1)	Stem Cell Research, Volume 21, 2017, pages 13-15, https://doi.org/10.1016/j.scr.2017.03.009	Three germ layer differentiation<br>Cardiomyocyte differentiation<br>Dopaminergic neuron differentiation<br>Dopaminergic progenitor cell differentiation<br>Otic progenitor cell differentiation
KICRi002-A (CTRL-10-I)	Stem Cell Research, Volume 18, 2017, Pages 22-25,ISSN 1873-5061, https://doi.org/10.1016/j.scr.2016.12.006.	Three germ layer differentiation
QC iPSC internal bank	ATCC #:ACS-1019, Lot #:70030238 expanded in house.	Three germ layer differentiation<br>Cardiomyocyte differentiation<br>Dopaminergic neuron differentiation<br>Dopaminergic progenitor cell differentiation<br>Otic progenitor cell differentiation
SCiPSR1	Episomally reprogrammed CD34 cell. Characterised against GAIT recommendations.	Three germ layer differentiation<br>Cardiomyocyte differentiation<br>Dopaminergic neuron differentiation<br>Dopaminergic progenitor cell differentiation<br>Otic progenitor cell differentiation
MCRIBi001-A (LO2 Cell 17 2.5d HiTemp)	Front. Cell Dev. Biol, Volume 10, 2022, Article 835321, https://doi.org/10.3389/fcell.2022.835321 Research grade iPSC derived from cord blood. Reprogrammed with Sendai Virus.	Three germ layer differentiation
YZWJ	YZWJ was derived from human cord blood and established with episomal vectors in CiRA_F. Culture methods are the same as those for QHJI, in QAR 2023 Cell samples.	Three germ layer differentiation<br>Otic progenitor cell differentiation
CFiS-S06	Research grade iPSC derived from peripheral blood. Reprogrammed with Sendai Virus.	Three germ layer differentiation<br>Cardiomyocyte differentiation<br>Dopaminergic neuron differentiation<br>Dopaminergic progenitor cell differentiation<br>Otic progenitor cell differentiation
CFiS-S04	Research grade iPSC derived from peripheral blood. Reprogrammed with Sendai Virus.	Cardiomyocyte differentiation<br>Dopaminergic neuron differentiation
CFiS-E03	Research grade iPSC derived from peripheral blood. Reprogrammed with episomal vectors.	Dopaminergic neuron differentiation

Three germ layer differentiation

Three germ layer differentiation was performed by using the STEMdiff™ Trilineage Differentiation Kit (STEMCELL Technologies, Cat No. 05230). The procedure followed the manufacturer's protocol. Briefly, iPSCs were plated and cultured for 5 days for endoderm and mesoderm differentiation, and 7 days for ectoderm differentiation, in the appropriate kit medium for inducing germ layer differentiation.

Cardiomyocyte differentiation

iPSCs were transferred to low adherent 6-well plates with aggregation medium (StemPro34 medium supplemented with 2 mM L-glutamine, 50 µg/ml ascorbic acid, 0.4 mM monothioglycerol, 150 µg/ml transferrin, 10 µM Y-27632 and 2 ng/ml BMP4). 24 hours later, EBs were formed and 2× differentiation medium 1 (StemPro34 medium with 2 mM L-glutamine, 50 µg/ml ascorbic acid, 0.4 mM monothioglycerol, 150 µg/ml transferrin, 18 ng/ml BMP4, 12 ng/ml Activin A and 10 ng/ml bFGF) was added to the plate. At day 3, the medium was replaced with Differentiation Medium 2 (StemPro34 medium supplemented with 2 mM L-glutamine, 50 µg/ml ascorbic acid, 0.4 mM monothioglycerol, 150 µg/ml transferrin, 10 ng/ml VEGF, 1 µM IWP-3, SB431542 and Dorsomorphin) and cells were maintained for 4 days. At day 7, the medium was changed to Differentiation Medium 3 (StemPro 34 medium supplemented with 2 mM L-glutamine, 50 µg/ml ascorbic acid, 0.4 mM monothioglycerol, 150 µg/ml transferrin and 10 ng/ml VEGF) and cells were cultured for 8 days. Culture medium was changed every 2-3 days.

Dopaminergic progenitor and neuron differentiation

iPSCs were plated onto iMatrix-coated 24-well plates at 1.0×10^6 cells/well in differentiation media containing Glasgow's minimum essential medium supplemented with 8% KSR, 0.1 mM MEM, 1 mM sodium pyruvate and 0.1 mM 2-mercaptoethanol. The differentiation medium was changed daily. The following were added to the differentiation medium: 100 nM LDN193189 on days 0-12; 500 nM A83-01 on days 0-6; 100 ng mL-1 fibroblast growth factor 8 and 2 µM purmorphamine on days 1-6; and 3 µM CHIR99021 on days 3-12. On day 12, cells were collected and seeded at 2.0×10^4 cells/well in 96-well round-bottom plates in the neural differentiation media containing NB/B27 medium supplemented with GDNF (10 ng/mL), 200 µM ascorbic acid, BDNF (20 ng/mL), dbcAMP (400 µM). Y-27632 (30 µM) was added at the time of seeding to form aggregate spheres. Every 2-3 days, the neural differentiation medium was changed until Day 26.

Otic progenitor cell differentiation

Otic progenitors were induced by plating iPSCs onto 6 well plates and cultured for 12 days in Dulbecco's Modified Eagle Medium: Ham's F12 (DMEM/F12) supplemented with 1× N2 and 1× B27, FGF3 and FGF10 (both growth factors 50 ng/ml).

Table S10. iPSC culture media used in the Validation Round 2024.

iPSC culture medium	Manufacturer
Essential 8™ Medium	Thermo Fisher Scientific
TeSR-E8 medium	STEMCELL technologies
iPS-Brew	Miltenyi Biotec
SFM XF/FF	ATCC
AK03N	Ajinomoto

Table S11. Cell culture coatings used in the Validation Round 2024.

Coating	Manufacturer
Laminin-521	BioLamina
Vitronectin	STEMCELL technologies
CellMatrix Basement Membrane Gel	ATCC
iMatrix-511	Nippi

Validation Round 2024 flow cytometry characterisation

Cells were fixed using Cytofix Fixation Buffer (BD Biosciences, Cat. No. 554655) and permeabilised with Perm/Wash™ Buffer (BD Biosciences, Cat. No. 554723) for intracellular staining. For extracellular antigens, unfixed cells were used. Antibodies targeting pluripotency-associated markers included Alexa Fluor® 488 Mouse anti-Human TRA-1-60 (BD Biosciences, Cat. No. 560173), SSEA4 Antibody, anti-Human, FITC, REAfinity® (Miltenyi Biotec, Cat. No. 130-122-918), Alexa Fluor® 488 Mouse anti-Oct3/4 (BD Biosciences, Cat. No. 560253), and SSEA5 (Miltenyi Biotec, Cat. No. 130-124-907). Primary and secondary antibodies used to assess cell type-specific differentiations are listed in Table S12. Staining was performed in PBS supplemented with 2% FBS for 30 min in the dark. Unstained cells served as negative controls. Samples were analysed using a FACSCanto II flow cytometer and FACS Diva software (BD Biosciences).

Table S12. Primary and secondary antibodies used in the Validation Round 2024. Markers and corresponding antibodies are listed for each differentiation assay, including three germ layer, cardiomyocyte, dopaminergic progenitor, dopaminergic neuron, and otic progenitor differentiation.

Expression tests	Marker	Antibody	Manufacturer	Cat. No.
Three germ layer differentiation	SOX17	Sox17, anti-human, PE	Miltenyi Biotec	130-111-032
	FOXA2	FoxA2, anti-human, APC	Miltenyi Biotec	130-123-850
	Brachyury	Brachyury (T), anti-human, Alexa Fluor 488	Cell Signaling Technology	94663S
	NCAM	CD56 (NCAM), anti-human, APC	STEMCELL Technologies	60021AZ
	NESTIN	NESTIN, anti-human, PE	BioLegend	656806
	PAX6	PAX-6 Antibody, anti-human, APC	Miltenyi Biotec	130-123-267
Cardiomyocyte differentiation	Cardiac Troponin T	Troponin T (Cardiac), monoclonal mouse, anti-human	R&D	MAB1874
	2nd Ab	Goat anti-mouse IgG (H+L) cross-adsorbed secondary antibody, Alexa Fluor 488	Thermo Fisher Scientific	A-11001
Dopaminergic progenitor cell differentiation	CORIN	CORIN, monoclonal mouse, anti-human	SIGMA	WH0010699M1
	2nd Ab	Goat anti-mouse IgG (H+L) cross-adsorbed secondary antibody, Alexa Fluor 488	Thermo Fisher Scientific	A-11001
Dopaminergic neuron differentiation	TUBB3	Tubulin β 3 (TUBB3), anti-human, Alexa Fluor 488	BioLegend	801203
	FOXA	Human HNF-3 beta /FoxA2, polyclonal goat, anti-human	R&D	AF2400
	2nd Ab	Donkey anti-goat IgG (H+L) cross-adsorbed secondary antibody, Alexa Fluor 647	Thermo Fisher Scientific	A-21447
Otic progenitor cell differentiation	SOX2	SOX2, monoclonal, anti-human, CoraLite® Plus 488	Proteintech	CL488-66411
	PAX8	PAX8 polyclonal, anti-human, CoraLite® Plus 647	Proteintech	CL647-10336

Supplemental Methods

Methods S1. QAR 2019 participant survey

iPSC Quality Assessment Round 2019 Participant Survey

iPSC Quality Round 2019 Technical Survey (including 'secondary testing' and 'sample reorder requests)

iPSC Quality Round 2019 Technical Survey (Estimated Time Burden 20-40 Minutes)

The survey covers two topics: (i) technical detail relating to your quality testing undertaken for the Quality Round and (ii) and repeat (secondary) testing including sample reissues.

Optional: Please note long answers (>300 characters) or images of gates, plots or other data can be uploaded to your data folder labelled as '[Institutional Codeword] - Technical Survey Answers'. Thank you.

1. What is your Iinstitutional code word?

[ ]

Section 1 (a): Immunostaining & Flow Cytometry Technical Questions for Participants

To help us better understand your data and its significance, please answer the following technical questions regarding how you carried out the flow cytometry analysis of your Quality Round Samples.

In order to process your answers efficiently and that the data is standardized for analysis and publication, we request that your answers are in a format similar to the example answers given.

2. Cell Count

Did you perform a cell count on each of the flow cytometry samples?

- ◯ no, cells were not counted prior to analysis
- ◯ yes, cells were counted before staining and washing
- ◯ yes, cells were counted after washing and staining

If you answered yes, please provide detail in the comment box below, otherwise leave it empty

[ ]

3. Antibodies

Have you checked specificity using positive/negative controls or with an isotype control antibody?

(Isotype controls are a type of negative control designed to measure the level of non-specific background signal caused by primary antibodies, based upon the tissue type of the sample. Usually, the background signal is the result of immunoglobulins binding non-specifically to Fc receptors present on the cell surface.)

- ◯ no, neither a positive/negative control or isotype control was included
- ◯ yes, a positive/negative control was included
- ◯ yes, an isotype control antibody was used

If you answered yes, please give details in the comment box below.

Sample answer:

Unstained controls were used as controls.

TRA-1-60 (negative control: unstained cells)

OCT3/4 (negative control: unstained cells)

SSEA4 (negative control: unstained cells)

4. Have you (or your tester) previously used the antibodies used in the Quality Round for evaluation of PSCs?

- ◯ no, I have not used these antibodies before
- ◯ yes, I have used some of the antibodies before (please specific which)
- ◯ yes, I have used all of the antibodies before

If you answered yes, please let us know for which antibodies.

Sample answer:

TRA-1-60: YES

OCT3/4: YES

SSEA4: YES

5. Immunostaining protocol

Primary and secondary antibodies are two groups of antibodies that are classified based on whether they bind to antigens or proteins directly or target another (primary) antibody that, in turn, is bound to an antigen or protein.

Please briefly describe your staining protocol, including permeabilization method for intracellular antigens, and whether single (conjugated) vs. primary/secondary antibody was used.

Sample answer:

For cell surface antigens (SSEA-3,SSEA-4, TRA-1-60):

Cells fixed with Cytofix™ Fixation Buffer (BD 554655) were stained with the single labeled antibody for 30 min in flow cytometry buffer (2% FBS in PBS).

Intracellular antigens (OCT4, NANOG):

Cells fixed with Cytofix™ Fixation Buffer (BD 554655) were permeabilized with Perm/Wash™ Buffer (BD 554723) for 15 min and stained with the single labeled antibody for 30 min in flow cytometry buffer (2% FBS in PBS).

6. Flow cytometry results analysis

Please describe how your gating procedure, including the information about the negative control; upload a representative image to your folder and reference it here if possible.

Sample Answer:

See Survey Sample Answer Figure 1 (www.gait.global/quality-round-primary-data-set).

After gating of main populations by FSC/SSC plotting and gating out abnormal events (like doublets), negative control (unstained sample) was used for establishing the positive signal threshold: the gate boundary was set as to exclude all events of negative control sample (figure). Then, the same gate was used for the stained sample.

Sample answer:

CELL SURFACE ANTIGEN: TRA-1-60, SAMPLE F1

UNSTAINED SAMPLE
TRA-1-60-STAINED SAMPLE
/INTRACELLULAR ANTIGEN: OCT3/4, SAMPLE F1

UNSTAINED SAMPLE
OCT3/4-STAINED SAMPLE

7. Did you perform any mathematical correction on the data (e.g. compansation for signal overlap between the channels of the emission spectra calculations or equalization of some sort?

- ◯ NO, no such mathematical corrections were made
- ◯ YES, mathematical corrections were made. Two flours with overlapping emission spectra had to be reconciled
- ◯ Details of mathematical corecction.

Sample answer:

Cells were stained with FITC SSEA-4 and with a PE isotype control, and collected at different compensation values to correct for the FITC spillover into the PE channel.

Represenative images of uncompensated and compensated cells have been uploaded to the Participant's data folder and labelled [Institutional Codename] - Compensation Data.

8. qPCR Operator

Please indicate how the genetic analysis of samples was performed:

- ◯ All runs were performed by the same operator
- ◯ Individual runs were performed by more than one operator

9. Please indicate the level of qPCR experience of operators:

- ◯ Experienced qPCR user (> 1 year experience prior to Quality Round)
- ◯ Moderate qPCR user (3 months - 1 year experience prior to Quality Round)
- ◯ Novice qPCR user (0 - 3 months experience prior to test)
- ◯ Multiple Operator Option: In cases where multiple operators performed qPCR for the Quality Round please indicate the corresponding run number next to the options in the comment box below.

  If only one operator performed the run, you can leave this comment box empty.

  Sample answer:

  Experienced qPCR user (1 run)
  Novice qPCR user (2 runs)

Section 2: OPTIONAL SECONDARY TESTING AND/OR SAMPLE REORDER REQUESTS

10. After you have assessed your data in comparison with other Participants' data, do you wish to undertake additional (secondary) testing?

- ◯ No, I do not wish to undertake secondary testing and will not be uploading further data
- ◯ Yes, I would like to retest samples and upload further Quality Testing data

11. If you wish to undertake additional testing and do you have sufficient samples for retesting?

Please note:

If you require additional NIBSC or CIRA flow cytometry samples these will be dispatched from NIBSC (at no extra charge for the sample and shipping).

If you require additional CiRA genetic analysis samples, these can be dispatched from CiRA at no extra charge for the sample – but dry ice shipping will be at Participant's expense - CiRA will organize on your behalf and bill you for the shipping.

- ◯ No, I do not wish to undertake additional testing and do not require additional samples
- ◯ Yes, I do wish to undertake additional testing but have sufficient sample matters for the retest. I need no additional samples sent to me
- ◯ Yes, I do wish to undertake additional testing but I require additional CiRA flow cytometry samples. I do not require additional CiRA flow cytometry samples or NIBSC flow cytometry samples
- ◯ Yes, I do wish to undertake additional testing but I require additional NIBSC flow cytometry samples. I do not require additional CiRA flow cytometry samples or CiRA genetic analysis samples
- ◯ Yes, I do wish to undertake additional testing but I require additional CiRA genetic analysis samples. I do not require additional CiRA nor NIBSC flow cytometry samples
- ◯ Yes, I do wish to I do wish to undertake additional testing but I require both CiRA and NIBSC flow cytometry samples. I do not require additional CiRA genetic analysis samples
- ◯ Yes, I do wish to I do wish to undertake additional testing but I require all CiRA and NIBSC flow cytometry samples and CiRA genetic testing samples

Thank you for completing the iPSC Quality Round Technical Survey (including 'sample testing' and 'sample reorder' requests).

Methods S2. QAR 2023 participant survey

iPSC Quality Assessment Round 2023 Participant Survey

The evaluator for your centre is asked to provide concise summary responses describing how their banks´ own activity meets the best practice described in the consensus guidance document ISCBI 2015.

https://www.futuremedicine.com/doi/full/10.2217/rme.14.93

The response can describe work under way to establish a system but it should be clear whether this is in process and not complete or yet to start.

If your centre is not yet actively generating iPSCs but are collecting cell and tissue sample you should only complete the relevant questions of the survey.

There are 5 parts to the survey:
(1) Donor Information and Donor Samples
(2) Preparation of induced Pluripotent Stem Cells
(3) Quality Control
(4) Safety Testing, and
(5) Characterisation

* Required

* This form will record your name, please fill your name.

Welcome

1. Institute Codename *

Donor screening & donor samples

ISCBI Guidance Section 3 Provenance and selection of donor tissue Pages 3-5

3.1 Donor Selection, Screening & Medical Records Page 3
3.2 Allogenic Cell Transplantation Page 4
3.3 Ongoing Donor Traceability Page 4
3.4 Advantageous Capture of Biological Specimens Page 4
3.5 Donor Medical Histories Page 5
Appendix 1(b) Informed Consent & Donor Disclosures Page 31

2. Do you have an informed consent process?

- ☐ Yes
- ☐ No
- ☐ Other

3. Do you use donor health and behaviour assessments and/or testing of donors for infectious agents eg. HIV, Hepatitis?

- ○ Yes
- ○ No
- ○ Other

4. If you answered 'yes' to the last question, please provide details below:

5. Is there a Standard Operating Procedure (SOP) for this infectious agent testing?

- ○ Yes
- ○ No
- ○ Unknown
- ○ Not Applicable (NA)
- ○ Other

6. Infectious agent testing is done:

- ◯ In house
- ◯ By an external contractor
- ◯ Both
- ◯ Not Applicable (NA)
- ◯ Other

7. Do you have a set of donor samples that you wish to be considered for inclusion in the GAiT haplobank system?

- ◯ Yes
- ◯ No
- ◯ Other

8. If you answered 'yes' to the last question, please provide details of this resource / these resources below:

9. If you are able to say, please state what reprogramming method(s) and starting materials you are routinely using below:

Induced Pluripotent Stem Cells Preparation

10. Have you prepared iPSC lines that you believe to be suitable for inclusion in the GAiT haplobank system?

- [ ] Yes
- [ ] No
- [ ] Other

11. If you answered 'yes' to the last question, please provide details of the cell viability tests used and the points at which cells are tested below:

12. Have you adopted any formal national or international quality standards in preparing the iPSC lines for inclusion into the GAiT haplobank system?

- ○ Yes
- ○ No
- ○ I have not made iPSC lines yet
- ○ Other

13. If you answered 'yes' to the last question, please provide details below:

Quality Control - Cell Viability & Identification Testing

14. Do you perform a test for Cell Viability?

- ☐ Yes
- ☐ No
- ☐ Other

15. If you answered 'yes' to the last question, please provide details of the cell viability tests used and the points at which cells are tested below:

16. Is there a Standard Operating Procedure (SOP) for this cell viability test?

- ○ Yes
- ○ No
- ○ Unknown
- ○ Not Applicable (NA)
- ○ Other

17. The cell viability test used is done:

- ○ In house
- ○ By an external contractor
- ○ Both
- ○ Not Applicable (NA)
- ○ Other

18. Do you perform a test for Cell Identity (e.g. DNA fingerprinting)?

- [ ] Yes
- [ ] No
- [ ] Other

19. If you answered 'yes' to the last question, please provide details of the cell identification tests used below:

20. Is there a Standard Operating Procedure (SOP) for this cell identification test?

- ○ Yes
- ○ No
- ○ Unknown
- ○ Not Applicable (NA)
- ○ Other

21. The cell identification test used is done:

- ○ In house
- ○ By an external contractor
- ○ Both
- ○ Not Applicable (NA)
- ○ Other

Safety Testing - Sterility testing of banked iPSC lines for viruses, bacteria & fungi

22. Do you perform a virus detection test on the banked iPSC lines?

- [ ] Yes
- [ ] No
- [ ] Other

23. If you answered 'yes' to the last question, please provide details of the viral detection tests used below:

24. Is there a Standard Operating Procedure (SOP) for this viral detection test?

- ◯ Yes
- ◯ No
- ◯ Unknown
- ◯ Not Applicable (NA)
- ◯ Other

25. The viral detection test? used is done:

- ◯ In house
- ◯ By an external contractor
- ◯ Both
- ◯ Not Applicable (NA)
- ◯ Other

26. Do you perform a a sterility test to detect bacteria, fungi and/or mycoplasma?

- ☐ Yes
- ☐ No
- ☐ Other

27. If you answered 'yes' to the last question, please provide details of the bacteria/fungi detection tests used below:

28. Is there a Standard Operating Procedure (SOP) for this bacteria/fungi detection test?

- ○ Yes
- ○ No
- ○ Unknown
- ○ Not Applicable (NA)
- ○ Other

29. The bacteria/fungi detection test used is done:

- ○ In house
- ○ By an external contractor
- ○ Both
- ○ Not Applicable (NA)
- ○ Other

Safety Testing - Tumorigenicity and Other Safety Tests

30. Do you perform a test for tumorigenicity?

- ☐ Yes
- ☐ No
- ☐ Other

31. If you answered 'yes' to the last question, please provide details of the tumorigenicity test used below:

32. Is there a Standard Operating Procedure (SOP) for this tumorigenicity test?

- ◯ Yes
- ◯ No
- ◯ Unknown
- ◯ Not Applicable (NA)
- ◯ Other

33. The tumorigenicity test used is done:

- ◯ In house
- ◯ By an external contractor
- ◯ Both
- ◯ Not Applicable (NA)
- ◯ Other

34. Do you use any tests to demonstrate the clearance of exogenous reprogramming materials from the banked iPSC cell lines eg. vector clearance?

- [ ] Yes
- [ ] No
- [ ] Other

35. If you answered 'yes' to the last question, please provide details of the additional safety tests used below:

36. Is there a Standard Operating Procedure (SOP) for this clearance test?

- ○ Yes
- ○ No
- ○ Unknown
- ○ Not Applicable (NA)
- ○ Other

37. The clearance test used is done :

- ○ In house
- ○ By an external contractor
- ○ Both
- ○ Not Applicable (NA)
- ○ Other

38. Do you perform any other safety test not already described?

- ○ Yes
- ○ No

39. If you answered 'yes' to the last question, please provide details of the additional safety tests used below:

40. Is there a Standard Operating Procedure (SOP) for this additional safety test?

- Yes
- No
- Unknown
- Not Applicable (NA)
- Other

41. The additional safety test used is done:

- In house
- By an external contractor
- Both
- Not Applicable (NA)
- Other

Characterisation - Genotyping & Pluripotency

42. Do you perform a genotyping test?

- [ ] Yes
- [ ] No
- [ ] Other

43. If you answered 'yes' to the last question, please provide details of the genotyping test used below:

44. Is there a Standard Operating Procedure (SOP) for this genotyping test?

- ◯ Yes
- ◯ No
- ◯ Unknown
- ◯ Not Applicable (NA)
- ◯ Other

45. The genotyping test used is done:

- ◯ In house
- ◯ By an external contractor
- ◯ Both
- ◯ Not Applicable (NA)
- ◯ Other

46. Do you perform a test for pluripotency?

- [ ] Yes
- [ ] No
- [ ] Other

47. If you answered 'yes' to the last question, please provide details of the pluripotency test used below:

48. Is there a Standard Operating Procedure (SOP) for this pluripotency test?

- ◯ Yes
- ◯ No
- ◯ Unknown
- ◯ Not Applicable (NA)
- ◯ Other

49. The pluripotency test used is done :

- ◯ In house
- ◯ By an external contractor
- ◯ Both
- ◯ Not Applicable (NA)
- ◯ Other

Characterisation - Phenotyping

50. Do you perform a phenotyping test?

- ☐ Yes
- ☐ No
- ☐ Other

51. If you answered 'yes' to the last question, please provide details of the phenotyping test used below:

52. Is there a Standard Operating Procedure (SOP) for this phenotyping test?

- ◯ Yes
- ◯ No
- ◯ Unknown
- ◯ Not Applicable (NA)
- ◯ Other

53. The phenotyping test used is done:

- ◯ In house
- ◯ By an external contractor
- ◯ Both
- ◯ Not Applicable (NA)
- ◯ Other

Characterisation - Cell stability/Epigenetics & Other Characterisation Test

54. Do you perform a cell stability/epigenetic characterisation test?

- ☐ Yes
- ☐ No
- ☐ Other

55. If you answered 'yes' to the last question, please provide details of the cell stability/epigenetic characterisation test used below:

56. Is there a Standard Operating Procedure (SOP) for this cell stability/epigenetic characterisation test?

- ◯ Yes
- ◯ No
- ◯ Unknown
- ◯ Not Applicable (NA)
- ◯ Other

57. The cell stability/epigenetic characterisation test used is done:

- ◯ In house
- ◯ By an external contractor
- ◯ Both
- ◯ Not Applicable (NA)
- ◯ Other

58. Do you perform any other characterisation test not already described?

- ☐ Yes
- ☐ No
- ☐ Other

59. If you answered 'yes' to the last question, please provide details of the additional characterisation tests used below:

60. Is there a Standard Operating Procedure (SOP) for this additional characterisation test?

- ◯ Yes
- ◯ No
- ◯ Unknown
- ◯ Not Applicable (NA)
- ◯ Other

61. The additional characterisation test used is done:

- ◯ In house
- ◯ By an external contractor
- ◯ Both
- ◯ Not Applicable (NA)
- ◯ Other

Technical Survey

* Required

* This form will record your name, please fill your name.

1. What is your Institute Codename? *

Quality Test 1

2. Did you perform a cell count on each of the flow cytometry samples?

- ◯ no, cells were not counted prior to analysis
- ◯ yes, cells were counted **before** staining and washing
- ◯ yes, cells were counted **after** staining and washing
- ◯ Other

3. If you answered yes to Question 2, please provide detail in the comment box below e.g method of counting/equipment type, otherwise leave it empty

4. Were there enough cells for you to run your procedure as you would routinely do?

- ◯ Yes
- ◯ No
- ◯ Other

5. How many technical replicates did you analyse per run?

6. How many runs did you complete?

- ◯ 2 complete runs
- ◯ 1 complete run only
- ◯ 1 complete run as first run failed.
- ◯ Other

7. What type of controls did you use?

- [ ] None
- [ ] Positive Controls
- [ ] Negative Controls - e.g unstained Cells
- [ ] Isotype Controls
- [ ] FMOs
- [ ] Other

8. If you answered yes, please give details in the comment box below.
Sample answer:
Unstained controls were used as controls.
TRA-1-60 (negative control: unstained cells)
OCT3/4 (negative control: unstained cells)
SSEA-4 (negative control: unstained cells)

9. Please briefly describe your staining protocol.

Optional: Please note long answers or supplemental data related to this survey can be uploaded to your data folder labelled as 'Codename_TechnicalSurvey9-1' (additional files can be numbered -2, -3 and so on)..
Thank you.

Sample answer:
- For cell surface antigens (SSEA-3, SSEA-4, TRA-1-60):
- Cells fixed with Cytofix™ Fixation Buffer (BD 554655) were stained with the single labelled antibody for 30 min in flow cytometry buffer (2% FBS in PBS).
- Intracellular antigens (OCT3/4, NANOG):
- Cells fixed with Cytofix™ Fixation Buffer (BD 554655) were permeabilized with Perm/Wash™ Buffer (BD 554723) for 15 min and stained with the single labelled antibody for 30 min in flow cytometry buffer (2% FBS in PBS).

10. Please describe how your gating procedure, including the information about the negative control; upload a representative image to your folder and reference it here.

Name file: Codename_TechnicalSurveyQ10-1 (additional files can be numbered -2, -3 and so on).

Sample Answer:

See file Codename_TechnicalSurveyQ10-1

Sample answer:
1. All cells by FSC/SSC
2. Doublet exclusion
3. Negative control e.g. unstained sample to set gates

Please note supplemental images of gates, plots or other data related to this survey can be uploaded to your data folder labelled as 'Codename_TechnicalSurveyQ10-1' (additional files can be numbered -2, -3 and so on).

11. Did you perform any mathematical correction on the data (e.g. compensation for signal overlap between the channels of the emission spectra calculations or equalization of some sort?

Sample answer:
Cells were stained with FITC SSEA-4 and with a PE isotype control, and collected at different compensation values to correct for the FITC spill over into the PE channel.

Representative images of uncompensated and compensated cells have been uploaded to the Participant's data folder and labelled 'Codename_TechnicalSurveyQ11-1' (additional files can be numbered -2, -3 and so on).

- ◯ NO, no such mathematical corrections were made
- ◯ YES, mathematical corrections were made. Two fluorophores with overlapping emission spectra had to be reconciled
- ◯ Other

12. If you answered YES to Question 11 please add details of mathematical correction.

Quality Test 2

13. How many assay runs did you complete?

- ◯ 2 complete runs
- ◯ 1 complete run only
- ◯ 1 complete run as first run failed.
- ◯ Other

Methods S3. QAR 2019 instructions to participants

Quality Round 2019

International External Quality Assessment Scheme (IEQAS) for human iPSC lines

Quality Assessment Round Instructions

Suppliers Instructions

Directions for iPSC Biomarker Expression Analysis by Flow-cytometry

UK Stem Cell Bank

Information for users for hPSC standards for flow cytometry.

Each vial contains ~ 1million PFA fixed hPSC cells. Each vial has 1 million cells, so participants can run up to 3-5 technical replicates as required.

They should be kept in the fridge at 4°C until ready to use.

The cells are fixed and stored in PBS without Ca++/Mg++; if nuclear staining is required a permeabilization step is necessary first.

If not, then the cells are ready to use.

Please used your local staining procedure. The standard can be used with any flow cytometer. Use your local gating strategies.

Please send back the data as % positive for each antibody tested, and the raw data as an FCS file if possible.

Images of representative plots are requested for upload to help Organizers with data amalgamation and interpretation.

NIBSC

Confidence in Biological Medicines

Supplied Instructions for Genomic Stability Analysis using StemCell Tech hPSC Genetic Analysis Kit

The kit supplied product and technical bulletin are included here for Participants convenience by STEMCELL Technologies.

qPCR analysis kit for detecting the majority of karyotypic abnormalities reported in human ES and iPS cells
Catalog #07550 60 Reactions

Product Description
hPSC Genetic Analysis Kit contains nine primer-probe mixes to detect the majority of recurrent karyotypic abnormalities reported in human embryonic stem (ES) cells and induced pluripotent stem (iPS) cells. This qPCR-based kit enables the genetic screening of multiple human ES and iPS cell lines in a rapid and cost-effective manner. It uses double-quenched probes with a 5-carboxyfluorescein (5-FAM) dye to give superior performance over other single-quenched probes.
hPSC Genetic Analysis Kit contains a Genomic DNA Control sample that has been validated as a diploid control for the regions analysed using this kit, as well as a separate ROX Reference Dye. The kit contains sufficient material to analyse 20 individual samples in triplicate (60 reactions).

Product Information
All components listed below are stable until expiry date (EXP) on label.

COMPONENT NAME	COMPONENT #	SIZE	STORAGE
qPCR Master Mix (2X)	07551	3 mL	Store at -20°C.
ROX Reference Dye	07552	0.2 mL	Store at -20°C.
Chr 1q Genetic Assay	07553	60 Rxn	Store at -20°C.
Chr 4p Genetic Assay	07554	60 Rxn	Store at -20°C.
Chr 8q Genetic Assay	07555	60 Rxn	Store at -20°C.
Chr 10p Genetic Assay	07556	60 Rxn	Store at -20°C.
Chr 12p Genetic Assay	07557	60 Rxn	Store at -20°C.
Chr 17q Genetic Assay	07558	60 Rxn	Store at -20°C.
Chr 18q Genetic Assay	07559	60 Rxn	Store at -20°C.
Chr 20q Genetic Assay	07560	60 Rxn	Store at -20°C.
Chr Xp Genetic Assay	07561	60 Rxn	Store at -20°C.
Genomic DNA Control	07562	15 µL	Store at -20°C.
TE Buffer	07563	1 mL	Store at -20°C.

Materials Required but Not Included

PRODUCT NAME	CATALOG #
Genomic DNA extraction kit	e.g. QIAGEN 69504
Costar® Microcentrifuge Tubes, 0.65 mL	38037
384-well qPCR plate	e.g. Sigma Z374911
Nuclease-free water	e.g. Sigma W4502
Optical adhesive film	e.g. Sigma Z707465

Preparation of Reagents and Materials
A. Harvesting Genomic DNA
Harvest genomic DNA from the cell line to be analysed using an appropriate genomic DNA extraction kit. If not used immediately, store harvested DNA at -20°C.
B. Master Mix + Dye
1. Thaw qPCR Master Mix (2X) and ROX Reference Dye on ice. Protect from light.
2. Add ROX Reference Dye to qPCR Master Mix (2X) according to Tables 1 and 2. For instruments not listed, refer to the manufacturer's instructions.

PCR SYSTEM	REFERENCE DYE CONCENTRATION LEVEL		
	HIGH	LOW	NONE
**Applied Biosystems**<br>• 7900HT Fast and 7300 Real-Time PCR Systems<br>• StepOne™ and StepOnePlus™ Real-Time PCR Systems	X		
**Applied Biosystems**<br>• ViiA™ 7 and 7500 Real-Time PCR Systems<br>• QuantStudio™ Flex		X	
**Agilent Technologies**<br>• Mx3005P and Mx4000P		X	
**Bio-Rad**<br>• CFX, iQ™, and DNA Engine Opticon® Real Time PCR Systems			X
**Roche**<br>• LightCycler® Real-Time PCR System			X

Table 1. Recommended Reference Dye Concentration Levels for PCR Systems

VOLUME OF MASTER MIX	VOLUME OF ROX REFERENCE DYE	
	High Reference Dye System (see Table 1)	Low Reference Dye System (see Table 1)
3 mL	120 µL	12 µL

Table 2. Volume of ROX Reference Dye to Add to Master Mix

3. Pulse vortex Master Mix + Dye on high for 3 - 5 seconds. Place on ice and protect from light.
NOTE: If not used immediately, aliquot and store at -20°C. Do not exceed the expiry date on the label. After thawing the aliquots, use immediately. Do not re-freeze.

hPSC Genetic Analysis Kit

C. Genomic DNA Control and Samples
1. Thaw Genomic DNA Control and samples on ice.

2. Measure the concentration of the genomic DNA samples to be analysed using a NanoDrop™ spectrophotometer or other method.
NOTE: At least two samples (or one sample plus the control) are required to determine copy number.
3. In a 0.65 mL microcentrifuge tube, add 1.5 µL of Genomic DNA Control to 56.5 µL of nuclease-free water (final concentration 5 ng/µL).
Vortex on high for 3 - 5 seconds. Place on ice.
4. Add 290 ng of genomic DNA sample to separate 0.65 mL microcentrifuge tubes and adjust the volume to 58 µL with nuclease-free water (final concentration 5 ng/µL). Place tubes on ice.
NOTE: If the concentration of genomic DNA samples is > 290 ng/ µL, it is recommended to dilute the sample using nuclease-free water so that an appropriate volume of genomic DNA is added (> 1 µL).
5. Vortex Master Mix + Dye (prepared in section B) on high for 5 seconds. Add 145 µL to each genomic DNA sample and the control (prepared in steps 3 - 4). Pipette up and down 2 - 3 times to mix. Place the tubes on ice and protect from light.
NOTE: Master Mix + Dye is viscous; pipette with care to avoid loss of material in the pipette tip.
D. Primer-Probe Mixes
1. Prepare the primer-probe stock solutions as follows:
a. Centrifuge the primer-probe sequences (e.g. Chr 4p Genetic Assay) at 750 x g for 10 seconds to ensure the contents are at the bottom of the tube.
b. Add 33 µL of TE Buffer to each tube. Pipette the solution up and down the sides of the tube to ensure complete resuspension.
NOTE: Use a separate pipette tip between Genetic Assays to avoid cross-contamination.
c. Centrifuge tubes at 750 x g for 10 seconds.
NOTE: If not used immediately, aliquot and store at -20°C. Do not exceed the expiry date on the label. After thawing the aliquots, use immediately. Do not re-freeze.
2. Calculate the number of reactions required for each primer-probe using the equation below:
Number of reactions per primer-probe = (Number of genomic DNA samples [including control] to be analysed x 3) + n
Where:
• For 2 - 5 samples, n = 2 OR
• For 6 - 10 samples, n = 3 OR
• For > 10 samples, n = 10% of number of samples
3. Prepare the primer-probe mixes by combining each primer-probe stock solution (prepared in step 1) with nuclease-free water in individual 0.65 mL microcentrifuge tubes. Refer to Table 3 for example volumes or calculate the volumes required as follows:
Volume of nuclease-free water = Number of reactions per primer-probe x 2.5 µL
Volume of primer-probe stock solution = Number of reactions per primer-probe x 0.5 µL
Vortex each tube on high for 5 seconds.

NUMBER OF SAMPLES	2	3	4	5	6	7	8	9	10
NUMBER OF REACTIONS	8	11	14	17	21	24	27	30	33
VOLUME OF NUCLEASE-FREE WATER (µL)	20	27.5	35	42.5	52.5	60	67.5	75	82.5
VOLUME OF PRIMER-PROBE STOCK SOLUTION (µL)	4	5.5	7	8.5	10.5	12	13.5	15	16.5
**TOTAL VOLUME (µL)**	**24**	**33**	**42**	**51**	**63**	**72**	**81**	**90**	**99**

Table 3. Example Volumes for Preparing Primer-Probe Mixes

Directions for Use
Please read the entire protocol before proceeding.
A. qPCR
1. Using a felt pen, mark a 3 x 9-well boundary for each sample on a 384-well PCR plate according to Figure 1 or Figure 2, depending on the number of samples. Tape the top edge of the plate to a microcentrifuge tube rack (or similar) to create an ~30º angle.

Figure 1. PCR Plate Array for up to 8 Samples

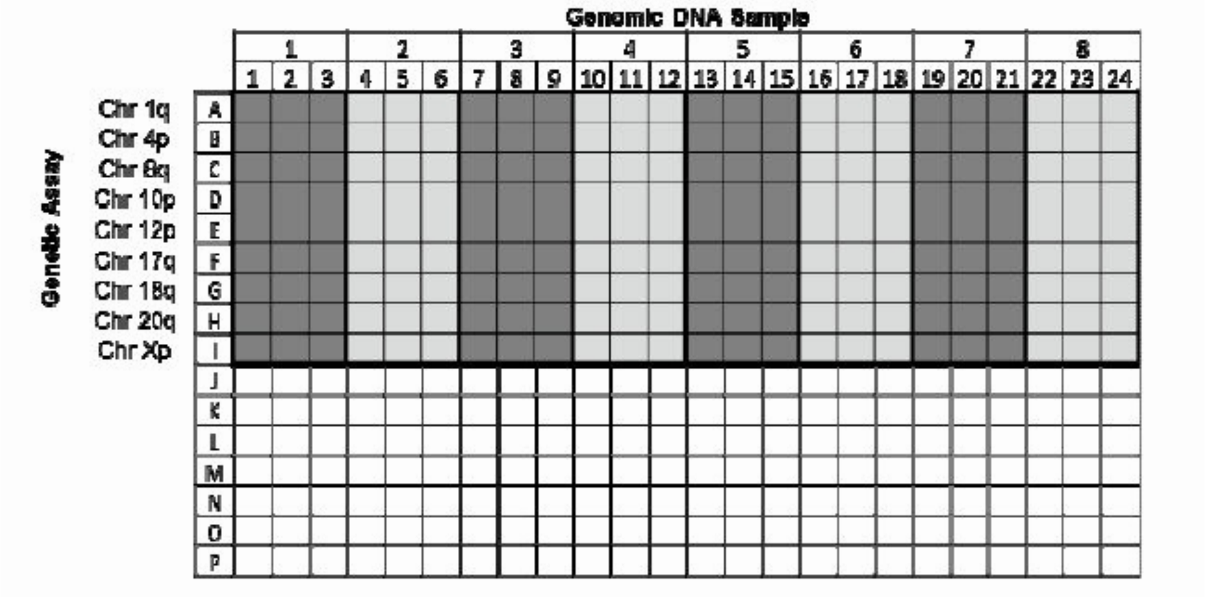

Figure 2. PCR Plate Array for 10 Samples

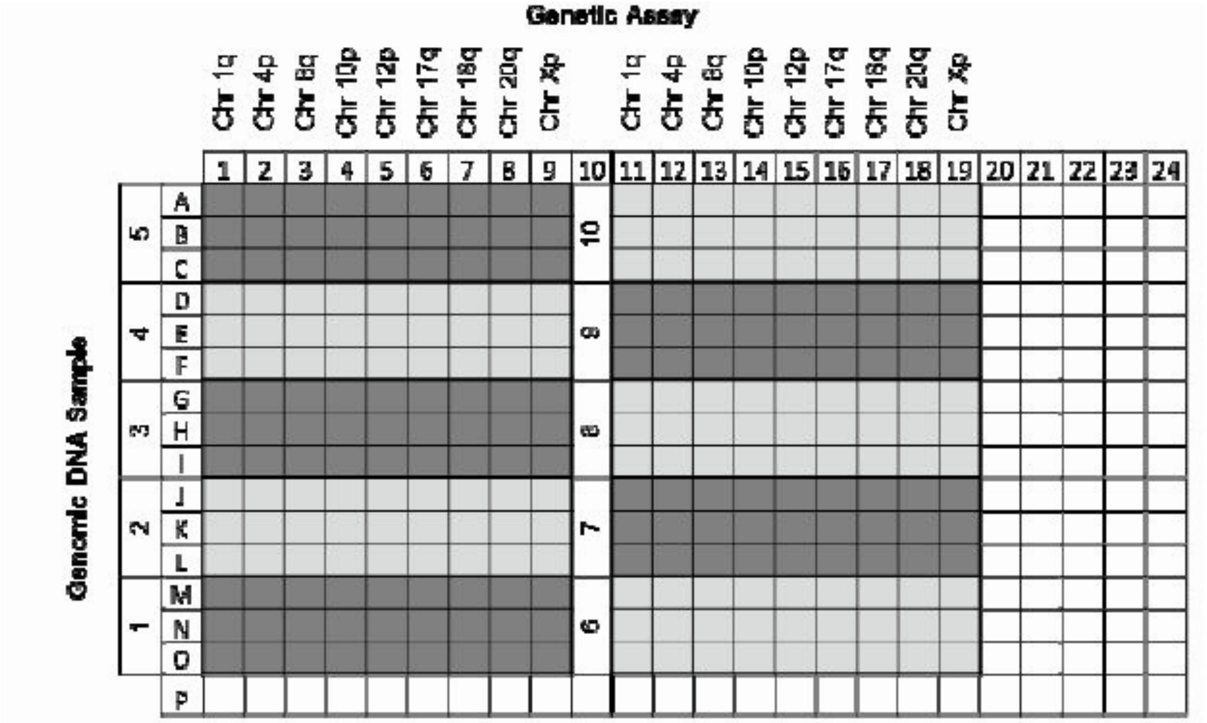

2. For the control and each genomic DNA sample prepared in section C (containing Master Mix + Dye), vortex on high for 5 seconds then centrifuge briefly to collect material at the bottom of the tube. Add 7 µL to the bottom edge of each well of the prepared plate, keeping within the marked 3 x 9-well boundary. Repeat for each sample until all samples have been loaded.
3. Working with one primer-probe mix at a time, vortex on high for 5 seconds, then centrifuge briefly to collect material at the bottom of the tube. Add 3 µL to the appropriate row of the plate, at the top edge of the well. Repeat until all primer-probe mixes have been loaded.
4. Cover the plate using optical adhesive film; use a scraper or roller to ensure that all edges and wells are sealed. Centrifuge the plate at 1000 x g for 1 - 2 minutes in a swinging bucket rotor fitted with plate holders to collect material at the bottom of the well.
5. Using a quantitative PCR system run the plate using the cycling conditions indicated in Table 4.

Table 4. PCR Cycling Conditions

STAGE	CYCLES	TEMPERATURE (°C)	FAST CYCLING TIME (min:sec)	STANDARD CYCLING TIME (min:sec)
Polymerase activation	1	95.0	03:00	03:00
Amplification:	40			
Denature		95.0	0:05	0:15
Anneal/extend		60.0	0:30	1:00

B. ANALYSIS OF RESULTS

Results obtained using hPSC Genetic Analysis Kit can be analysed using the application available at www.stemcell.com/geneticanalysisapp.

NOTE: Organize the Ct values (also referred to as Cq values) into the format shown in Figure 3; the data can then be pasted directly into the table in the application.

Figure 3. Data Organization for Analysis

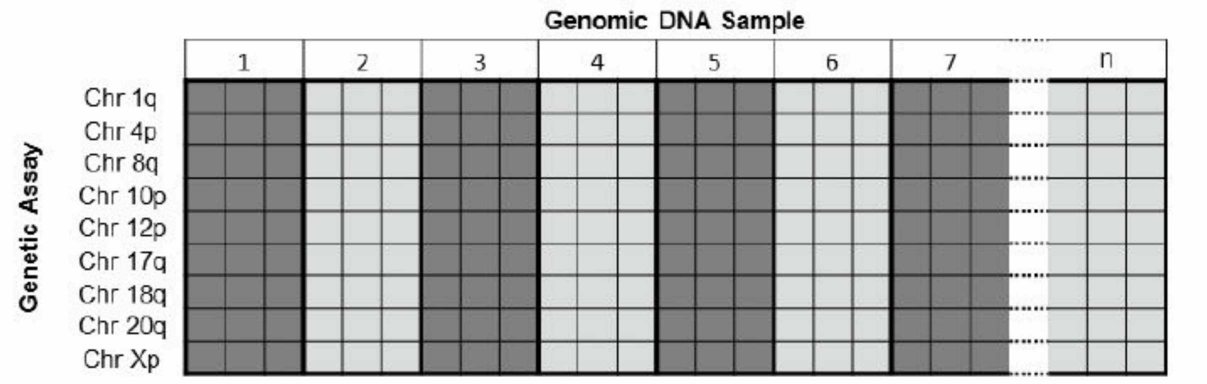

Alternatively, data can be analysed using the methods described below.

1. Calculate $\Delta\Delta$Ct values as follows:

Subtract the replicate Ct values of each Genetic Assay from the average Ct value of Chr 4p Genetic Assay.

NOTE: This calculation normalizes the data within each sample to account for any differences in DNA concentration between samples.

2. Calculate $\Delta\Delta$Ct values as follows:

Subtract the replicate Ct values of each test sample from the average Ct value of the Genomic DNA Control sample for each Genetic Assay.

NOTE: This calculation normalizes the data to a known control sample and will be the basis of determining copy number.

3. Calculate copy number as follows:

Copy number = $(2-\Delta\Delta Ct) \times 2$

NOTE: A copy number < 1.8 or > 2.2 with a p-value* < 0.05 may indicate the presence of an abnormality within the culture.

NOTE: High variability between technical replicates will significantly impact downstream analysis. This is particularly important in any Genetic Assay within the control sample and also for the Chr 4p Genetic Assay within test samples, as this data is used for normalization.

*p-values can be calculated across all loci using a one-way ANOVA with a tukey post-hoc test. Alternatively, an unpaired t-test can be performed between the Chr 4p Genetic Assay control region and the locus of interest.

TECHNICAL BULLETIN

hPSC GENETIC ANALYSIS KIT

TECHNICAL TIPS & Recommendations

This document contains tips and recommendations for optimal use of the hPSC Genetic Analysis Kit. This document should be used in conjunction with the Product Information Sheet (PIS; Document #DX22330).

Key Tips

• When using the hPSC Genetic Analysis Kit for the first time, run the Genomic DNA Control and only 1 - 2 samples to become familiar with the protocol before screening large numbers of samples.

• Vortex the mixtures thoroughly (5 seconds per sample) when indicated in the PIS.

• It is important to be precise and careful when pipetting. A digital single-channel repeating (multi-dispenser) pipette is recommended when analysing multiple samples.

General Tips

When using the kit for the first time

1. Read the PIS thoroughly before resuspending the Genetic Assays or preparing the qPCR Master Mix + Dye.

2. The first run should be carried out on the Genomic DNA Control plus 1 - 2 samples at most. Once you are familiar with the technique and have low replicate variability, the sample number can be increased.

3. Plan your plate layout in advance and have it in front of you for reference when pipetting the reactions.

Vortex, Vortex, Vortex!

1. When working with DNA there is often a hesitation about vortexing, since it may lead to shearing. Although we do not recommend vortexing the stock genomic DNA samples, it is important to thoroughly vortex the DNA and Master Mix + Dye solution (prepared in step C5) for 5 seconds. This will help to reduce the variability between technical replicates.

2. Likewise, the Genetic Assay and water solution prepared in step D3 should be sufficiently mixed by vortexing (5 seconds per sample). This helps to reduce the variability between technical replicates.

Genomic DNA Sample Preparation

1. The amount of genomic DNA per reaction can be increased; in some cases, this can improve reproducibility between replicates; e.g. instead of using 290 ng in 58 µL (step C.4), use 580 ng or 870 ng in 58 µL.

2. Mix the genomic DNA and Master Mix + Dye, prepared by the end of C5, by vortexing thoroughly (5 seconds per sample) immediately before pipetting into the plate.

3. Determine the concentration and quality of genomic DNA samples using an appropriate method. Genomic DNA samples should have absorbance ratios in the range of A260/280 ~1.8- 2.0 and A260/230 ~1.9 - 2.2.

4. If the concentration of the sample DNA is low (< 5 ng/µL), it is beneficial to concentrate using ethanol precipitation or other suitable method prior to analysis with the hPSC Genetic Analysis Kit. Running very low concentrations of DNA may introduce high variability between replicates and lead to difficult-to-interpret results.

Genetic Assays

1. The lyophilized primer-probe pellet may have become dislodged during shipping, so it is important to centrifuge the tube prior to reconstitution. If possible, try to locate the pellet and pipette the TE Resuspension Buffer directly onto the pellet for resuspension.

2. It is important to mix the Genetic Assay thoroughly before use, particularly if the suspension is going to be aliquoted. This can be done by flicking the tube followed by brief centrifugation.

3. Take care when pipetting the Genetic Assays; always use a clean pipette tip when moving between stocks or diluted assays to avoid cross-contamination of primer-probes.

Troubleshooting

qPCR Reaction Fails/No Amplification

All reactions fail to amplify:

• Incorrect amount or no ROX Reference Dye added to qPCR Master Mix (2X).

Note: Check that the correct amount of ROX Reference Dye was added to the qPCR Master Mix (2X) for the machine being used.

• Incorrect cycling conditions used.

Note: If you are unsure which cycling conditions are required for your machine, use the standard cycling times provided in the PIS.

Individual reactions fail to amplify:

• One or more components are missing from the reaction including genomic DNA, qPCR Master Mix (2X), ROX Reference Dye, or Genetic Assay.

Note: Take care when pipetting the reactions together; marking sample borders can help to keep track of wells that have been filled.

• Incorrect wells selected in the qPCR software.

Note: Some qPCR machines use software that require the pre-selection of wells and their contents; check that the correct wells are selected prior to performing qPCR.

High Variability Between Technical Replicates

• Inaccurate pipetting of samples/reagents.

Note: Properly calibrated repeating pipettes can be used to reduce variability.

• Insufficient mixing/vortexing of reagents.

Note: Where indicated in the PIS, mixtures should be sufficiently mixed by vortexing.

• Insufficient volume of mixture.

Note: If prepared correctly, the genomic DNA + qPCR Master

Mix solution created in step C and the Genetic Assay mixes created in step D should have sufficient excess material to allow for loss during pipetting.

• Inaccurate loading of reagents into qPCR plate.

Note: Take care when pipetting the reagents into the qPCR plate; loading variable amounts of DNA between wells will affect amplification dynamics.

Data Format Requirements

- Raw flow-cytometry data should be in FCS format and raw rtPCR data should be in the format below and should be uploaded to the GAiT.global Quality Round portal at www.gait.global/qualityrounddataportal. A GAiT username and password is required to access this section of the GAiT website.
- rtPCR should be uploaded as an Excel spreadsheet with the following headings: TE, HE, TE-HE, TC-HC, ΔCTE, & ΔCTC, & ΔΔCt.
- Cell line nomenclature in the format suggested by Kurtz et al. (2018) [1].

Quality Round Data Portal

NIBSC Standards Reporting Template

- Note: Report back in % positive
- Use local antibodies/flow machine/gating strategies
- Please also send back raw data file

Cell Line 1

Antibody	Vial 1	Vial 2	Vial 3
Oct 4			
Nanog			
Sox-2			
SSEA4			
SSEA1			
Tra-160			
Antibody XXX			
Antibody XXX			
Antibody XXX			

Cell Line 2

Antibody	Vial 1	Vial 2	Vial 3
Oct 4			
Nanog			
Sox-2			
SSEA4			
SSEA1			
Tra-160			
Antibody XXX			
Antibody XXX			
Antibody XXX			

Cell Line 3

Antibody	Vial 1	Vial 2	Vial 3
Oct 4			
Nanog			
Sox-2			

SSEA4			
SSEA1			
Tra-160			
Antibody XXX			
Antibody XXX			
Antibody XXX			

Flow Cytometer Used:XXX

Antibody	Supplier	Catalogue Number
Oct 4		
Nanog		
Sox-2		
SSEA4		
SSEA1		
Tra-160		
Antibody XXX		
Antibody XXX		
Antibody XXX		

References

1. Kurtz A, Seltmann S, Bairoch A *et al*. A Standard Nomenclature for Referencing and Authentication of Pluripotent Stem Cells. *Stem Cell Reports* 10(1), 1-6 (2018).

Methods S4. QAR 2023 Quality Test 1 and 2 instructions to participants

QUALITY ASSESSMENT ROUND 2023

A Quality Assessment Scheme

for human induced Pluripotent Stem Cell lines

Quality Test 1 Instructions

The samples can be used with any flow cytometer. Use your local gating strategies, reagents and protocol.

Quality Assessment Round (QAR) Participants are required to test the QAR samples with a minimum of two markers from the standard hPSC panel (positive for OCT4, TRA-1-60, TRA-1-81, SSEA-3, SSEA-4, SOX2, NANAOG) as they normally do as part of the iPSC quality testing. A combination of at least one intracellular (e.g., OCT4, SOX2 or NANOG) and one extracellular (e.g. SSEA-4 or TRA-1-60, TRA-1-81) is required.

Ensure **all events** are recorded and save data as **FCS files**.

Analysis for additional markers, that comprise QAR Participant's routine iPSC quality testing, should also be included and shared. When nuclear staining, Participants are asked to use their own standard protocol, as they normally would. It is important to fill out the data templates thoroughly so QAR Organisers can independently analyse and interpret the results appropriately.

Please upload to your GAiT portal folder:

- The data as **% positive** for each antibody tested using the template provided
- The raw data as **FCS files**
- The **plots or histograms (graphs)** of analysed data

Sample Information and Template Completion

Samples provided for Quality Test 1 are as follows:

Set 1: Cell 1-X、Cell 1-Y、Cell 1-Z

Set 2: Cell 2-X、Cell 2-Y、Cell 2-Z

Cell samples should be stored at or below -80°C until ready to use.

Set 1 and Set 2 cells **cannot** be mixed. Please use one complete set per run.

Each vial contains up to **4.3 x 10^6 PFA fixed cells** in 0.8 mL CellBanker. As a Quality Test run requires 1 complete set (3 different cell type; X,Y and Z), participants have enough samples to complete up to 2 runs.

The sample sets provided for Quality Test 1 can be used as follows:

- Individually for two separate runs (Run 1 and Run 2 on the template)
  OR
- The second set can be used as a backup if error occurs with the first set of cells.

PLEASE ENSURE YOU CLEARLY RECORD THE LABEL OF EACH SAMPLE ANALYSED ON THE TEMPLATE (1X, 1Y, 1Z, 2X, 2Y or 2Z)

Run definition: A run is defined as an individual assay set up, either performed by different operators or by the same operator on a different occasion.

Replicate definition: A replicate is a test of the same sample within a run.

The number of replicates within each run will be determined by each Institute's protocol, all results should be added to the template and the total mean % positive events for each antibody tested should also be calculated:

For example:

Run 1 Sample 1X (% Positive events for each antibody)

Antibody Name (e.g SSEA-4 FITC)	Replicate 1 (%)	Replicate 2 (%)	Replicate 3 (%)	Mean (%)
SSEA-4 FITC	97.2	92.4	94.6	94.7

Note: Please add additional columns or rows to the template to ensure all data is captured. For example, if more replicates or antibodies are included.

Naming of Data Files for Upload to GAiT Website Portal

Data template sheets should be labelled in the following format:

[INSTITUTIONAL CODENAME]_Quality Test (QT) [Quality Test number]. Examples:

Butterfly_QT 1 would be data template Quality Test 1 from Participant codenamed 'butterfly'

Raw data files should be labelled in the following format:

[INSTITUTIONAL CODENAME] Quality Test (QT) [Quality Test number]_[sample numbers]_[epitope qualifier if necessary]_[Run number]_[Replicate number]

Examples:

Glowworm_QT1_1X_OCT4_Run1_Rep2 would be raw data from Participant codenamed 'Glowworm' for Quality Test 1 for samples 1X, stained for OCT4 tested in Run 1 as sample replicate 2.

Spider_QT1_2Y_OCT4_SSEA-4_Run2_Rep3 would be raw data from Participant codenamed 'Spider' for Quality Test 1 for samples 2Y, stained for OCT4 and SSEA-4, tested in Run 2 as sample replicate 3.

Data Analysis plots/histograms should be labelled in the following format:

[INSTITUTIONAL CODENAME] Quality Test (QT) [Quality Test number]_[sample numbers]_[epitope qualifier if necessary]_[Run number]_[Replicate number]_GRAPH

Examples:

Glowworm_QT1_1X_OCT4_Run1_Rep2_GRAPH would be the graph from Participant codenamed 'Glowworm' for Quality Test 1 for samples 1X, stained for OCT4 tested in Run 1 as sample replicate 2.

Quality Test 2 Instructions

Protocol for undifferentiated marker expression analysis by flowcytometry for the Quality Assessment Round 2023.

This protocol is based on the CiRA_F validated quality test for undifferentiated marker expression of iPSCs and has been modified with reference to the methods of each Participant from the Quality Assessment Round kick off meeting and experts' comments. To reduce human error, the protocol is simplified compared to what is routinely performed. Although daily tests in CiRA_F are performed with live cell/fixed cells, to enable all participants to use the same cell samples, only fixed cells will be provided from CiRA_F. In this Quality Assessment Round, we will verify whether equivalent analysis can be carried out by 23 institutions.

Each vial contains up to **4.3 x 10^6 PFA fixed cells** in 0.8 mL CellBanker. As a single test needs 1 set (3 different cell type; X, Y and Z), participants have enough samples to complete up to 2 runs.

Equipment required:

PBS
FACS buffer (if you wish to use)
1.5 mL tube
15 mL tube
50 mL tube
Flow cytometry tubes
Nylon mesh strainer/ filter (≧35 µm)
Adjustable pipette
Pipette tips
Liquid nitrogen storage or deep freezer
Water bath
Centrifuge
Flow cytometer

Kit components

Antibodies:

Antibody-A (Ab-A): 2 tubes 35 µL each
Antibody-B (Ab-B): 2 tubes 10 µL each
Antibody-C (Ab-C): 2 tubes 70 µL each
Antibody-D (Ab-D): 2 tubes 35 µL each
Storage temperature: 4°C
All antibodies are conjugated with Alexa Fluor® 488 or FITC

Cryopreserved fixed cell:

Set 3: Cell 3-X、Cell 3-Y、Cell 3-Z

Set 4: Cell 4-X、Cell 4-Y、Cell 4-Z

Cell samples should be stored at or below -80°C until ready to use.

Set 3 and Set 4 cells **cannot** be mixed. Please use one complete set per run.

Buffer solution:

Buffer-K (10x): 4 tubes 9 mL each

Storage temperature: 4°C

Procedure

1. Tube and buffer preparation

1. Dilute 4 mL of Buffer-K (10x) with 36 mL of PBS in a 50 mL tube
2. Label 3 of 15 mL tubes as 3X, 3Y and 3Z for cell suspension (4X, 4Y and 4Z for set 4)
3. Label 5 of 1.5 mL tubes for Ab-A, Ab-B, Ab-C, Ab-D and Ab-U for staining buffer
4. Label 15 of 1.5 mL tubes for cell staining as on the list below

Tube labeling list:

Tube	Label
Cell Suspension 15ml tubes (3)	3X
	3Y
	3Z
Antibody Staining Buffer 1.5ml tubes (5)	Ab-A
	Ab-B
	Ab-C
	Ab-D
	Ab-U
Cell Staining 1.5ml tubes (15)	XA
	XB
	XC
	XD
	XU
	YA
	YB
	YC
	YD
	YU
	ZA
	ZB
	ZC
	ZD
	ZU

2. Staining buffer preparation

1. Add the following volumes of antibody and buffer-K (X1) to the labelled Staining buffer tubes
2. Mix well by tapping

**Staining buffer tube label**	**Ab-A**	**Ab-B**	**Ab-C**	**Ab-D**	**UAb**
Buffer-K (X1) (μL)	90	100	90	90	100
Antibody: volume (μL)	Ab-A: 9	Ab-B: 2	Ab-C: 18	Ab-D: 9	-
Total (μL)	99	102	108	99	100

Keep the diluted antibodies in a dark place at 4°C until ready to use.

3. Preparation of cell samples

1. Add 5 mL of Buffer-K (x1) into the 3 cell suspension tubes (15 mL) each
2. Thaw **one set** of cells X, Y and Z vials (one vial each) in a 37 °C water bath immediately after taking the vials from the storage.
3. Transfer the cells into the cell suspension tube.
4. Split the cell suspension into 5 cell staining tubes (1 mL / tube)

For Example:

Cell 3-X:		Cell 3-Y:		Cell 3-Z:	
	XA		YA		YA
	XB		YB		YB
	XC		YC		YC
	XD		YD		YD
	XU		YU		YU

(Total 15 tubes)

4. Staining procedure

1. Centrifuge at 200 x g for 5 min and remove the supernatant
2. Add the following:

**Antibody**	**Tubes**
30 μL of Ab-A	XA, YA, ZA
30 μL of Ab-B	XB, YB, ZB
30 μL of Ab-C	XC, YC, ZC
30 μL of Ab-D	XD, YD, ZD
30 μL of Ab-U	XU, YU, ZU

3. Incubate at RT for 15 min in a dark place.
4. Mix cell suspension by tapping and stand at for another 15 min in a dark place (30 min incubation in total)
5. Add 1mL of Buffer-K (x1) to each tube
6. Centrifuge at 200 x g for 5 min and remove the supernatant
7. Resuspend stained cells to 250 μL of PBS or FACS Buffer
8. Filter cell suspensions through a nylon mesh strainer to a flow cytometry tube
9. Store at 4°C in a dark place until the start of flow cytometric measurements

5. Acquisition, Analysis

Acquire the respective stained cells on the validated flow cytometer (at least 10,000 events at P3)

1. **P1 gating**: Create a forward scatter (FSC)-Area vs side scatter (SSC)-Area density plot and gate on the population of interest
2. **P2 gating**: Create an FSC-Height vs HSC-Width density plot, show only the P1 gated population, and gate on the single-cell population
3. **P3 gating**: Create an SSC-Height vs SSC-Width density plot, show only the P2 gated population, and gate on the single cell population
4. Create a FITC-A vs Counts histogram and show only the P3 gated cell population. Draw a gate to set the range of fluorescence values that will define FITC positive Ab stained cells

**Unstained Control**	**Samples**			
XU	XA	XB	XC	XD
YU	YA	YB	YC	YD
ZU	ZA	ZB	ZC	ZD

UAb stained cells show the background level of fluorescence as a negative control. Overlay of the UAb stained population onto the Ab stained population allows easy identification of the FITC positive cells

5. Obtain the mean fluorescence intensity (MFI) of FITC and the percentage of marker expression in the P3 gated population.
6. Save raw data and analysed data as FSC files

Please upload to your GAiT portal folder:

- The data as **% positive** for each antibody tested using the template provided
- The raw data as **FCS files**
- The **plots or histograms (graphs)** of analysed data

Template completion

The sample sets provided for Quality Test 2 can be used as follows:

- Individually for two separate assay runs (Run 1 and Run 2 on the template)

  OR
- The second set can be used as a backup if error occurs with the first set of cells

PLEASE ENSURE YOU CLEARLY RECORD THE LABEL OF EACH SAMPLE ANALYSED ON THE TEMPLATE (3X, 3Y, 3Z, 4X, 4Y or 4Z).

Run definition: A run is defined as an individual assay set up, either performed by different operators or by the same operator on a different occasion.

Naming of Data Files for Upload to GAiT Website Portal

Data template sheets should be labelled in the following format:

[INSTITUTIONAL CODENAME]_Quality Test (QT) [Quality Test number]. Examples:

Butterfly_QT 2 would be data template Quality Test 2 from Participant codenamed ‘butterfly’

Raw data files should be labelled in the following format:

[INSTITUTIONAL CODENAME] Quality Test (QT) [Quality Test number]_[sample numbers]_[Antibody]_[Run number]

Examples:

Glowworm_QT2_3X_A_Run1 would be raw data from Participant codenamed 'Glowworm' for Quality Test 2 for samples 3X, stained for Ab-A tested in Run 1.

Spider_QT2_4Y_B_Run2 would be raw data from Participant codenamed 'Spider' for Quality Test 2 for samples 4Y, stained for Ab-B, tested in Run 2.

Data Analysis plots/histograms should be labelled in the following format:

[INSTITUTIONAL CODENAME] Quality Test (QT) [Quality Test number]_[sample numbers]_[Antibody]_[Run number]_GRAPH

Examples:

Glowworm_QT2_3X_A_Run1_GRAPH would be graph from Participant codenamed 'Glowworm' for Quality Test 2 for samples 3X, stained for Ab-A tested in Run 1.
